# Coupling between adhesome assembly and 3D chromatin organization

**DOI:** 10.1371/journal.pone.0361202

**Published:** 2026-10-29

**Authors:** Louis Vincent Cammarata, Duhita Sengupta, Rajshikhar Gupta, Caroline Uhler, G. V. Shivashankar

**Affiliations:** 1 Department of Statistics, Harvard University, Cambridge, Massachusetts, United States of America; 2 Broad Institute of MIT and Harvard, Eric and Wendy Schmidt Center, Cambridge, Massachusetts, United States of America; 3 Paul Scherrer Institut, Villigen, Switzerland; 4 Department of Health Science and Technology, ETH Zürich, Zürich, Switzerland; 5 Massachusetts Institute of Technology, Laboratory for Information and Decision Systems, Cambridge, Massachusetts, United States of America; 6 Massachusetts Institute of Technology, Institute for Data, Systems, and Society, Cambridge, Massachusetts, United States of America; University of Stuttgart, GERMANY

## Abstract

Proteins on the cell membrane cluster to respond to extracellular signals; for example, adhesion proteins cluster to enhance extracellular matrix sensing. The maturation of such receptor clusters may require additional adhesome proteins to adapt and reinforce extracellular signal sensing. However, it has been unclear whether the genes encoding these proteins share a spatial organization in the nucleus. Using the adhesome as an example, we show that subsets of genes that code for adhesome proteins exhibit relative spatial clustering and co-regulation in distinct locations within the nucleus. We use Hi-C maps and RNA-seq data from adherent cells to map the correspondence between adhesome gene localization and their expression. Interestingly, we find that the genes encoding several transcription factors, cofactors, and chromatin regulators that regulate these genes are also co-localized with the adhesome gene loci, thereby potentially suggesting a link between the extracellular matrix sensing machinery and gene expression. Additionally, we validate the spatial sub-clustering of adhesome gene loci using MERFISH data and DNA-FISH experiments. Collectively, our results support an association between adhesome gene organization and regulatory state, with a possible role in cellular signal sensing.

## 1 Introduction

Cells must respond to various physical and biochemical signals in our tissues [1]. To achieve this, the plasma membrane of cells comprises several sensors including receptors, ion channels, and others [2]. Using such sensors, cells can rapidly adapt to varying signals in the microenvironment via, e.g., cell matrix adhesion through integrin receptors [3], cell-cell interactions through E-cadherin receptors [4], or antigen binding through T-cell receptors [5]. A common underlying mechanism in such signal sensing and adaptation requires the rapid clustering of the receptors and various protein adaptors to reinforce signal sensing and cellular response [6–8]. For example, in the context of integrin-mediated mechanotransduction pathways [9], adhesion-induced RhoA signaling results in nuclear translocation of various transcription factors, leading to the activation of genes coding for integrins and focal adhesion docking proteins [10,11]; adhesion-induced RhoA signaling also results in MRTF-A/B nuclear translocation, upregulating a set of genes necessary for stress fibers and mature adhesions [12,13]; FAK is another focal adhesion protein which shuttles between focal adhesions and the nucleus upon biomechanical stimulation, where it interacts with various transcription factors leading to changes in gene expression, including adhesion-related genes [14]. The feedback between focal adhesion maturation and transcriptional output has also been studied in the context of cell motility and migration; the transcriptional co-activators YAP and TAZ mediate this feedback by modulating cytoskeletal and focal adhesion maturation to enable persistent cell motility [15]. We hypothesized that genes encoding adhesome components are spatially clustered and have similar regulatory profiles, and that loci encoding their transcriptional regulators are spatially proximal to adhesome target genes. We tested these predictions by comparing active adhesome genes with active non-adhesome genes in fibroblasts.

Recent progress in genomic and proteomic technologies have allowed the characterization of various proteins involved in signal sensing and transduction [16–19] as well as measuring the spatial organization of genomes [20–23]. For example, many of the proteins involved in cell matrix sensing to assemble focal adhesions have been annotated. In addition, recent technological developments in genomics coupled with efforts in bioinformatics have resulted in the annotation of many of the transcription factors, cofactors and chromatin regulators that participate in regulating adhesome genes [24–26]. Furthermore, recent progress in chromosome conformation capture assays have allowed mapping the contact frequencies of the 3D organization of genomes in multiple cell types [21,27,28]. Additionally, fluorescence in situ hybridization (FISH) technologies have enabled the identification of the spatial location of particular genes and chromosomes in single cells [29,30], thereby validating the 3D organization of genomes obtained using chromosome conformation capture methods. Importantly, advances in sequencing methods have allowed to obtain not only high resolution maps of the spatial organization of the genome, but also the transcriptional state of the whole genome [31,32]. Given the vast datasets that exist in the context of focal adhesion, we used adherent fibroblast cells to test this hypothesis.

Chromosomal conformation capture technologies provided early evidence for the spatial clustering of functionally related genes (located on different chromosomes). A data-driven study of the human lymphoblastoid cell line GM06990 revealed that genes belonging to the same functional group (e.g., genes that code for interacting proteins, participate in the same complex, or pathway) tend to colocalize in space [33]. More recently, interchromosomal genomic clusters that are both spatially colocalized and coregulated were identified in the IMR-90 cell line [34]. These findings align with the condensate and transcription factory model according to which actively transcribed genes across multiple chromosomes cluster around sites of highly concentrated transcription machinery [35], possibly being held together through non-coding RNA [36] and transcription factor binding [37]. They are also consistent with experimental studies of the past two decades describing the spatial clustering of olfactory receptor genes, globin genes, immunoglobulin genes, Hox genes, heat shock protein genes, and Plasmodium virulence genes, among other functional gene groups [38]. Interchromosomal spatial clustering could be an efficient way to reinforce the co-regulation of these gene groups in a cell type-specific manner [39,40], as shown for KLF1-regulated genes [41] and NFκB-responsive genes [42].

In this work, we investigate the spatial organization of genes encoding adhesome proteins and their regulators in the nucleus of IMR-90 cells. Using transcriptomic data, we first confirm that most adhesome genes are active in adherent cells. Using chromosome conformation capture data, we then show that the active adhesome genes are spatially clustered in the cell nucleus as compared to other active non-adhesome genes. Interestingly, the adhesome genes also show strong transcriptional co-regulation. We then leverage transcription regulator binding data together with chromosome conformation capture data to show that the genes encoding transcriptional regulators of the adhesome genes are also strongly clustered with these genes. These different data modalities enable identifying tight clusters of adhesome genes and their transcriptional regulators, which we further validate using publicly available genome-wide FISH data, and DNA-FISH experiments on selected genes. These findings support an association between adhesome gene organization and regulatory state.

## 2 Materials and methods

### 2.1 Adhesome data

Adhesome data was retrieved from http://adhesome.org/, a literature-based protein-protein interaction network made of known interactions and cellular components constituting the focal adhesion complex in mammalian cells [43,44]. Study results for multiple mammalian cell types were used to construct this database, which is not specific to the IMR-90 cell line. The network includes 150 intrinsic components and 82 associated components, with a total of 6,542 interaction records (S1B Fig). The distinction between intrinsic and associated components is based on the location of the molecule: intrinsic components reside in the adhesion complex, while associated components transiently belong to the adhesion complex.

### 2.2 Regulatory data and processing

IMR-90 bulk mRNA-seq data [45] was retrieved from the Gene Expression Omnibus (GEO) under the accession number GSE16256 (sample GSM438363). Data from 47 cistromic marks were also obtained from ref [46]. who collected it from ENCODE [32], Roadmap Epigenomics [31], and the GEO, and released it on the TargetFinder GitHub page (link). In order to obtain the genomic profile for a gene, the number of peaks overlapping the gene’s location on the hg19 reference genome (obtained from ref [47].) was calculated for each feature using the Python pybedtools library. The profile vector of each gene was then divided by the gene length to account for the random incidence bias. For each feature, the feature matrix was then log(1 + 1000000x)-transformed, centered and standardized by computing the mean and standard deviation of the feature across all genes in the genome. To determine gene activity in GM12878, we also retrieved and processed as described above bulk RNA-seq data from the GEO under the accession number GSE90276 (hg19-aligned gene quantifications in log(1 + TPM) format from samples GSM2400247 and GSM2400248) as well as 100 regulatory marks from ref [46].. The likely category of histone ChIP-seq marks (active, repressive, unknown) was retrieved from ref [48]. and ref [49]., and the category of transcription factors and other protein ChIP-seq marks were inferred from protein function.

Gene activity was determined by hierarchical clustering with average linkage and Pearson correlation of the processed regulatory profiles; the two resulting clusters were annotated using RNA-seq expression and known active and repressive marks.

### 2.3 Hi-C data and processing

The Hi-C contact frequency matrices for IMR-90 and GM12878 from ref [28]. were obtained from the GEO (accession number GSE63525) at 250kb resolution and mapping quality MAPQ≥30. Intrachromosomal matrices were corrected for locus-specific bias using the Knight-Ruiz matrix balancing algorithm [50] using the Juicebox software (link). Interchromosomal matrices were also corrected for locus-specific bias using a matrix balancing approach (see ref [28]. for more details). Namely, we ran the Knight-Ruiz matrix-balancing algorithm on the genome-wide contact matrix at resolution 250kb (GWKR normalization). Following normalization, centromeric regions retrieved from ref [51]. were filtered out in each interchromosomal Hi-C matrices, along with peri-centromeric regions within 2Mb of the centromere and outliers based on row and column sums (outside of the 1.5xIQR, where IQR denotes the interquartile range). Repeat regions from ref [51]., as well as regions already masked in ref [28]. were also removed from the analysis. The final Hi-C matrix was log(1 + x)-transformed. When we run the LAS algorithm, we further centerdize the final Hi-C matrix using the mean and standard deviation of contact values across the entire genome.

### 2.4 Topologically associating domains

We followed the steps outlined in ref [52]. to call TADs with the Arrowhead software [28] at a resolution of 5 kb, using a Knight-Ruiz (KR) normalization of intrachromosomal Hi-C maps. TAD search was limited to a sliding window of size 2,000 along the Hi-C matrix diagonal. In a subsequent processing step, we removed nested TADs and merged overlapping TADs. Overall, this process identified 4,443 TADs located on different chromosomes (except for chromosome 9), covering ~35% of the genome. The resulting average TAD length is 225 kb, which is similar to the 250 kb Hi-C resolution used throughout this study. Illustrations and descriptive statistics of the TAD calling process can be found in S2 Fig. Diagnostic metrics [52] showed that the resulting TAD list outperformed TAD lists obtained using different resolutions (5 kb, 10 kb, 25 kb) or different Hi-C processing methods (KR normalization, no normalization). Computing the Measure of Concordance between these TAD lists revealed that normalization had a limited impact on TAD calling. Then, investigating the enrichment of CTCF, RAD21, and SMC3 (cohesin complex proteins) at the TAD boundaries showed that TAD partitions obtained at the 5 kb resolution exhibited the highest quality. Finally, enrichment of H3K36me3 (active mark) and H3K27me3 (repressive mark) in the TAD lists (measured as the log10-ratio between their ChIP-seq signals and compared with a null distribution derived from random permutations) confirmed the high quality of TAD lists obtained at the 5 kb resolution. These diagnostic metrics are shown in S3 Fig.

### 2.5 DNA-MERFISH data

We retrieved DNA-MERFISH data from ref [30], where 1,041 genomic loci were simultaneously imaged in 5,400 IMR-90 cells across 5 biological replicates with a detection efficiency of ~80% for each locus, yielding on average ~1,700 probe loci detected in each cell. The probe loci used for DNA-MERFISH are ~30 kb in size and cover the 22 autosomes and chromosome X approximately uniformly, with each chromosome containing 30–80 loci. The data is freely available on Zenodo. Maximum probe–probe distances were calculated for 3,668 cells with valid coordinate and identifier data. Subsequent removal of records with missing values across all columns, together with chromosome-X exclusion, retained 3,533 cells and 990 loci for downstream analysis. For this analysis, we considered loci and gene locations with respect to the hg38 reference genome; the gene location data was retrieved from the UCSC Genome Browser [47,51].

Probe loci were associated with active adhesome or adhesome TR genes within 500 kb upstream or downstream; sensitivity analyses used a 100-kb window or required direct gene overlap. To account for different cell sizes, probe-probe Euclidean distances in each cell were normalized by the maximum probe-probe distance in that cell (S4B Fig). The mean distance for each pair was computed over the cells in which both probes were observed (S4C Fig). For the heatmap comparisons, active non-adhesome probe loci were sampled to match the number of adhesome-associated probes per chromosome. An active probe locus was defined as lying within the corresponding mapping window of an active gene in IMR-90. The histogram comparison included all other active non-adhesome probe loci.

### 2.6 Transcription regulators-target data

Transcription regulators target data was retrieved from ref [24]. containing a curated TR-target dataset from large-scale ChIP-seq data of human TRs (7,190 samples of 659 TRs) in 569 conditions (399 cell lines, 129 classes of tissues or cells, and 141 kinds of treatment). The data also includes inferred TR-target links obtained from TR binding site scanning. We scraped this data for several tissues, including the lung, from the online repository of hTFtarget (https://guolab.wchscu.cn/hTFtarget/#!/) using the Python package Requests.

### 2.7 Protein-protein interaction data

Protein-protein interaction data used in S5E Fig was retrieved from the STRING v11 interactome [53], restricted to physical interactions only. The interaction strength is a measure of confidence combining probabilities from different evidence channels (experiments, text mining, data bases).

2.8 Statistics

- Correlation test: In Fig 2C and S4E Fig, we used a two-sided correlation test described in ref [54]. to assess the significance of the Pearson correlation between scaled Hi-C contact values and the regulatory correlation based on 48 regulatory features for two groups of genes: active adhesome genes (blue) and a randomly selected set of active non-adhesome genes (red).
- Multivariate two-sample test: In Fig 2C, we also tested for the difference between the joint distribution of scaled Hi-C contact values and Pearson regulatory correlation for the two gene groups of interest: active adhesome genes (blue) and the randomly selected set of active non-adhesome genes (red). We used a two-sample test based on the number of nearest neighbor type co-incidences, as described in ref [55].. We created a 1-Nearest Neighbor graph among genes from both groups using the Euclidean distance to determine neighboring relationships. Then, we computed the number of edges between genes of the same group. We compared this statistic to a simulated null distribution where we computed the same statistic under random permutation of gene labels, for 100 different permutations (see S6E Fig). We reported the one-sided *p*-value for that null distribution.
- Hypergeometric test: In Fig 3B, we plotted each TR of interest based on the percentage of genes it targets among all active genes (x-axis) and the percentage of genes it targets among all active adhesome genes (y-axis). To identify important TRs, we adopted a null hypergeometric model for each TR, conditionally on the number of genes it targets among all active genes. Under that null model, the number of active adhesome genes targeted by the TR follows a Hypergeometric(*M*,*n*,*N*) distribution where *M* is the total number of active genes, *n* is the total number of active adhesome genes and *N* is the number of active genes targeted by the TRs. For each TR, we computed the corresponding one-sided *p*-value. In Fig 3B, we also shaded in grey the area corresponding to non-significant TRs at the 0.05 significance level, after applying a Bonferroni correction to control the Family-Wise Error Rate (FWER).
- Cosine similarity: For hierarchical clustering, we can measure affinity between two genes using the cosine similarity between their feature vectors. The cosine similarity between two vectors *u* and *v* is equal to <u,v>/(‖u‖·‖v‖)</u,v>. It is a scoring metric to measure the similarity between *u* and *v*, ranging from −1 (vectors in opposite directions) to 1 (vectors aligned in the same direction); a value of 0 corresponds to orthogonal vectors.

### 2.8 Large average submatrices (LAS) algorithm

The LAS algorithm [56] takes a real-valued data matrix *X* (of size *m*-by-*n*) as input and outputs contiguous submatrices *U* (of size *k*-by-ℓ) that have a high average, τ, when compared to independent standard Gaussian entries. Therefore, the Hi-C contact frequencies were log-transformed and standardized using the mean and standard deviation of the non-zero contact frequencies across the entire genome. The resulting distribution of non-zero transformed Hi-C contact values in the entire genome is reported in S7A Fig; we note that the LAS algorithm is robust to slight violations of the normality assumption.

We used a version of the LAS algorithm implemented in ref [34]., which specifically detects contiguous submatrices rather than arbitrary submatrices. A detailed description of the algorithm is provided in the Supplementary Information of ref [34].. In our work, the LAS algorithm search space was limited to contiguous submatrices of at most 2Mb-by-2Mb in size, i.e., 8-by-8 submatrices (at 250kb resolution). For each chromosome pair, each iteration of the search procedure was initialized at a random contiguous *k*-by-ℓ submatrix in the log-transformed and standardized interchromosomal Hi-C map, such that the initial submatrix contained at least one active adhesome gene or adhesome TR gene. There are 3,990,387,344 submatrices of size at most 8-by-8 in the collection of interchromosomal Hi-C maps corresponding to all pairs of autosomes. To control the Family-Wise Error Rate (FWER) of the procedure at 0.0001, we used a Bonferroni correction and identified submatrices whose LAS *p*-value was below 0.0001/3,990,387,344 = $2.5\cdot 10^{-14}$. To be even more conservative, we applied a *p*-value cutoff of 10^–15^, as shown in S7B Fig.

The resulting distribution of average Hi-C contacts of the submatrices selected by the LAS algorithm is displayed in S7C Fig, where we also show a Hi-C cutoff at 2 (dashed red vertical line). We used this threshold to discard submatrices detected by LAS whose average Hi-C contact value was low. For example, the LAS submatrices identified for the chromosome pair 19–20 before and after applying the Hi-C threshold are reported in S7D–S7E Fig. Thresholding based on the average Hi-C value alone is slightly different from thresholding based on the *p*-value, because the *p*-value depends on both the average Hi-C value and the dimension of the submatrix.

### 2.9 Gene ontology (GO) enrichment analysis

GO enrichment analyses were performed using the GSEApy Enrichr [57–59] module with the GO_Biological_Process_2018 gene set in humans.

### 2.10 Cell culture

Human fibroblast cells GM09503 were grown in DMEM (Gibco; Life Technologies), supplemented with 10% (vol/vol) FBS (Gibco; Thermo Fisher Scientific), NEAA (Gibco; Thermo Fisher Scientific), Glutamax (Gibco; Thermo Fisher Scientific) and 1% penicillin–streptomycin (Gibco; Thermo Fisher Scientific). Cells were maintained in 5% CO2 at 37 °C and cultured overnight on fibronectin-coated cleaned glass slides.

### 2.11 DNA FISH experiments

Cells were incubated in ice cold pure methanol for 20 minutes at −20°C followed by an incubation in 2×SSCT (here 2×SSC is 0.3M sodium chloride and 30mM trisodium citrate; and therefore, 2×SSCT is 2×SSC with 0.1% Tween-20) for 5 minutes. The slides were incubated in prewarmed (at 92°C) 2×SSCT + 50% (v/v) formamide (Sigma for 2.5 minutes followed by another incubation in prewarmed (at 60°C) 2×SSCT + 50% (v/v) formamide (Sigma, Cat no. F7503) for 2.5 minutes. 50 μl of hybridization solution (Sigma, Cat no H7782) with 40 pmol of each myTags probe (Atto 594nm adhesome Core), (Atto 488nm neg control) (Arbor Biosciences, Cat no. 418002) was added to a 25×25 mm coverslip. The slide was slowly inverted onto the coverslip and sealed. The probes were then incubated with cells on the slide in a humid environment at 92° for 2.5 minutes and transferred to a humid environment at 42°C for hybridization overnight. Following hybridization slides were rinsed in prewarmed (at 60°C) 2×SSCT for 15 minutes followed by two more rinses in 2×SSCT at RT for 10 minutes and in 2×SSC at RT for 10 minutes.

### 2.12 Immunostaining and confocal imaging

The slides were blocked and immustained with RNA POLII CTD repeat YSPTSPS (ab5408: Anti-RNA polymerase II CTD repeat YSPTSPS (phospho S5) antibody [4H8] – ChIP Grade, Mouse Monoclonal). After the final washing step, samples were mounted with Prolong Gold Antifade mounting medium (Life Technologies, USA) according to the manufacturer’s protocol. Slides were allowed to cure completely before imaging. The coverslips (0.17 ± 0.005 mm, Deckglasser 1.5H high precision) were properly sealed. Slides were scanned using a Nikon A1 Confocal microscope (Nikon, USA) with a Plan apochromatic λ 60X oil immersion objective (Camera: Nikon A1 LFOV, Microscope: Ti2 Microscope, Numerical Aperture: 1.4, Refractive Index: 1.515, Scanner Zoom: 2.899, Scanner: Galvano, Pinhole Size: 26.82, Scan speed: 0.25).Confocal z-stack images were captured with a voxel size of 0.07×0.07×0.5 $\mu m^{3}$ with z slices ranging across 8 μm. The nuclei were selected through random field selections scanning the whole slide. More than 100 nuclei were analyzed per sample.

### 2.13 Image analysis

The nuclear mask was obtained via nuclear segmentation using multi-thresholding. The adhesome gene loci were segmented using the intensity and volume-based threshold. For intensity based threshold, pixels with intensity more than *Th* were first binarized; here *Th* is given by the expression: Th=μ(I)+σ(I), where μ(I) is the voxel-wise mean and σ(I) is the voxel-wise standard deviation of the intensity distribution of $I_{AdhsomeFISH}$, $I_{ControlFISH}$, and $I_{RNAPOL2}$ in the segmented nuclei regions. For volume based threshold, gene loci were identified to be the distinct segmented regions with volume greater than 0.5 $\mu m^{3}$. The pairwise distance was calculated on the centroid of gene loci. The dilated mask of adhesome gene loci was obtained by using slice-wise dilation using a disk element of 8 pixels. The pRNA Pol2 intensity was evaluated on the dilated mask of adhesome gene loci. All the image analysis was done using the open-source Python scikit-image [60] library. We computed a centric radial distribution function (RDF) by using the geometric center of the nuclear binary mask bounding box as the reference point. For each segmented object, the distance from its centroid to this center was calculated and binned into concentric annuli of equal radial width. The number of objects in each annulus was normalized by the annular area and the mean particle density, such that centric radial distribution function = 1 corresponds to a uniform spatial distribution. Similarly, we evaluated the particle radial distribution function (PRDF) using computed all pairwise distances between their centroids. Distances were grouped into radial bins of equal width, and the counts in each bin were normalized by the annular area and the expected number of neighbors based on the global object density. Under a spatially uniform (Poisson) distribution, this normalization yields *PRDF* = 1, whereas deviations from 1 indicate spatial clustering (*PRDF* > 1) or depletion (*PRDF* < 1) at the corresponding distance scales.

## 3 Results

### 3.1 Genes coding for focal adhesion proteins that are clustered on the cell membrane show strong spatial clustering in the cell nucleus

We sought to analyze the proximity of adhesome genes in an adherent cell type, using IMR-90, a human female fetal lung fibroblast cell line as a model system. As a first step, we obtained a curated list of 232 proteins that reside directly or indirectly in adhesion sites from different cell types [43]. These proteins include integrins, actin regulators, adaptor proteins that link the cytoskeletal structures to the cytoplasmic tails of integrins, as well as a variety of signaling molecules (S8 Fig, S1 File). Gene set enrichment analysis confirmed the key role of these genes in regulating cell-matrix adhesion, migration, and cytoskeletal organization, among others (S1A Fig). A number of studies have shown that adhesome proteins are densely linked via binding or signaling interactions, suggesting that these proteins have to work together in the adhesome complex [44] (S1B Fig, S2 File, S9 Fig). In our work, we focused on adhesome genes located on the autosomes 1–22, thus leaving out from the analysis five adhesome genes located on chromosome X (*ARHGEF6*, *FLNA*, *MSN*, *SH3KBP1* and *SMPX*).

Next, we analyzed expression data of adherent cells to verify that the adhesome genes are actively being transcribed. For this, we retrieved from previous studies bulk RNA-seq data [45], as well as preprocessed data on 47 cistromic marks [46] for IMR-90 cells. The regulatory marks included chromatin accessibility data (DNase-seq) and a selection of histone and transcription proteins ChIP-seq data, among others (S10A Fig). This data was collectively used to determine the activity status of genes in IMR-90. In particular, we identified regulatory signals corresponding to 25,959 genes, whose genomic locations were obtained from a previous study [47]. We applied hierarchical clustering with average linkage to this data to identify two clusters of genes, cluster 1 containing 14,122 genes and cluster 2 containing 11,837 genes (S10B Fig and Methods). These two clusters are depicted in the t-distributed Stochastic Neighborhood Embedding (t-SNE) plot of Fig 1A. By comparing the activity status of each of the two gene clusters based on the distribution of RNA-seq expression, we identified cluster 1 to correspond to active genes and cluster 2 to correspond to inactive genes (Fig 1B); the difference in expression is highly significant (Wilcoxon rank-sums test, *p*-value≤ $3\cdot 10^{-308}$) and consistent with peaks from known active marks (e.g., POLR2A, H3K36me3) and repressive marks (e.g., H3K9me3, H3K27me3).

**Fig 1:**
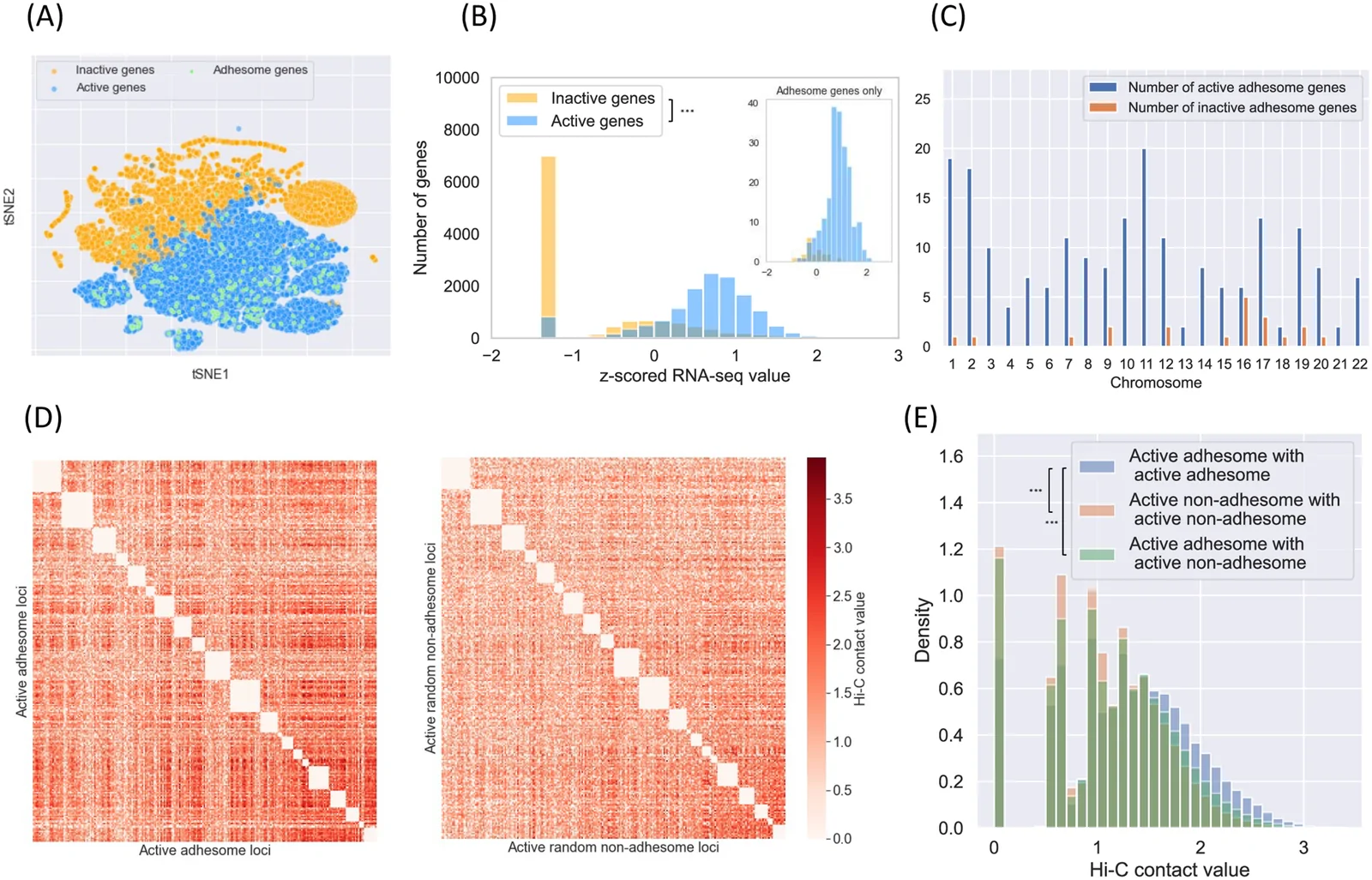
Adhesome genes are active and spatially clustered in the nucleus of IMR-90 cells. (A) tSNE representation of 25,959 genes based on 48 IMR-90 regulatory features. Orange dots correspond to inactive genes (as determined by the corresponding expression of regulatory marks), and blue dots correspond to active genes. Out of the 222 adhesome genes studied (green dots), 202 are active. (B) Histogram of RNA-seq expression in IMR-90 for all active genes (blue) and all inactive genes (orange). Active genes have a significantly higher expression than inactive genes (*p*-value≤ $3\cdot 10^{-308}$, Wilcoxon rank-sums test). The embedded graph in the upper right corner corresponds to the histogram of RNA-seq expression in IMR-90 for all active adhesome genes and all inactive adhesome genes. Active adhesome genes have a significantly higher expression than inactive adhesome genes (*p*-value≤ $2\cdot 10^{-9}$, Wilcoxon Rank-Sums test). (C) Bar plot of the number of active (blue) and inactive (orange) adhesome genes per chromosome. (D) Interchromosomal Hi-C contact matrices for active adhesome loci (left) and a randomly selected set of active non-adhesome loci chosen on the same chromosomes (right). For robustness, an active locus is defined to be a locus that contains at least one active gene and that was labeled as active in the locus clustering. (E) Histograms of Hi-C contact frequencies across all active adhesome loci (blue), all active non-adhesome loci (orange), and between active adhesome loci and active non-adhesome loci (green). Hi-C contacts are significantly higher among active adhesome loci compared to active non-adhesome loci (*p*-value≤ $3\cdot 10^{-308}$, Wilcoxon rank-sum test) and active adhesome-active non-adhesome loci (*p*-value≤ $3\cdot 10^{-308}$, Wilcoxon rank-sum test).

Of the 232 curated adhesome components, 222 genes could be matched to the autosomal hg19 gene annotation used for downstream analysis [47,51]. Of these, 202 genes were found to be active in IMR-90 cells. These active adhesome genes are represented as light green dots in the t-SNE plot of Fig 1A. This representation was obtained using available transcriptomic data as well as data on 1D genomic marks (due to limited public data availability, noncoding RNAs, which are known to play a role in chromatin tethering [38,61], could not be considered in this analysis). For comparison, conducting a similar analysis in GM12878, a human lymphoblastoid cell line, showed that only 172 adhesome genes were identified as active in this non-adherent cell line (S11 Fig). While, as expected, this number is lower compared to IMR-90, a good proportion of adhesome genes remain active in GM12878 as they participate in other signaling pathways in this cell type. For example, the B cell receptor (BCR) signaling pathway is mostly active in GM12878, which is a B-lymphoblastoid cell line (63 BCR genes are active out of 81 in our data); this pathway is related to other signaling pathways [62–64] including actin cytoskeleton organization, MAPK signaling, NF-κB signaling and calcium-mediated signaling, which contain genes that overlap with active adhesome genes in GM12878 (S12 Fig).

The identified adhesome genes are distributed on all 22 autosomes (Fig 1C), suggesting that interchromosomal interactions may be essential for their co-regulation. To test if active adhesome genes are spatially co-clustered in the cell nucleus, we assessed the proximity of the 202 active adhesome genes in IMR-90 across chromosomes. This was carried out using publicly available genome-wide chromosomal conformation capture data assayed with the in situ Hi-C protocol [28]. While the Hi-C data set allows resolution up to 5 kb, we chose to study interactions on a 250 kb contact map, since this resolution allows to locate genes of interest while reasonably controlling the sparsity of interchromosomal Hi-C data. Hi-C preprocessing is described in Methods.

We compared the distribution of interchromosomal Hi-C contact frequencies among active adhesome loci (defined as loci containing active adhesome genes) and among all active non-adhesome loci (defined as loci containing active non-adhesome genes, but no active adhesome genes). Throughout our work, interchromosomal interactions refer to non-homologous chromosomal contacts (NHCCs) [65]. Fig 1D shows heatmaps of interchromosomal Hi-C contacts between all active adhesome loci (left) as compared to interchromosomal Hi-C contacts between a set of randomly chosen active non-adhesome loci (right). To obtain an unbiased comparison, we used the same number of active adhesome and non-adhesome loci on each chromosome. It is apparent from these heatmaps that Hi-C contacts are higher among active adhesome loci. As expected, contacts tend to be higher for the small, gene-rich autosomes [27] (chromosomes 16–22, lower right corner of Fig 1D’s left sub-panel), whose chromosomal territories are preferentially located in the center of the nucleus, while the chromosome territories of larger chromosomes are usually found at the nuclear periphery [66].

Fig 1E shows the distribution of Hi-C contacts among all active adhesome loci (blue histogram) and among all active non-adhesome loci (orange histogram). Adhesome loci were found to be significantly closer in the cell nucleus compared to non-adhesome loci (*p*-value≤ $3\cdot 10^{-308}$, two-sided Wilcoxon rank-sums test). Additionally, by comparing the distribution of Hi-C contacts among active adhesome loci to the distribution of Hi-C contacts between active adhesome loci and active non-adhesome loci (green histogram), we ruled out the possibility that active adhesome loci interact at a high frequency with other loci in general, and confirmed that our findings are specific to the adhesome. The active adhesome genes that exhibit the highest proximity in Hi-C (top percentile) are shown in the network of S13 Fig, containing 74 genes distributed on 18 chromosomes connected by 321 edges. These genes participate in many adhesome-related pathways, in particular the transmembrane receptor protein tyrosine kinase signaling pathway.

Because Topologically Associating Domains (TADs) are a well-known unit of chromosomal organization, we sought to reproduce this finding at the level of TADs instead of 250 kb genomic loci. 4,443 TADs were called at 5 kb resolution, including 134 TADs containing 132 active adhesome genes (see Methods). These TADs are referred to as adhesome TADs in the remainder of this paper, and their Hi-C contacts are shown in S14B Fig (top). For comparison, we identified random non-adhesome TADs that are similar in size, similarly distributed over the chromosomes, and have a similar transcriptional expression level. S14E Fig confirms that the distribution of interchromosomal contacts between active adhesome TADs significantly dominates the distribution of interchromosomal contacts between random TADs (Wilcoxon Rank-Sum test, *p*-value≤ $5\cdot 10^{-124}$).

Interestingly, spatial clustering of active adhesome loci was not found at the intrachromosomal level. These loci are not significantly closer on the linear genomic sequence than randomly chosen active genes on the same chromosomes, and they do not exhibit increased intrachromosomal Hi-C contact values; see S15 Fig. Consistent with this observation, active adhesome genes rarely belong to the same TADs. Overall, this finding positions interchromosomal contacts as an important level of organization for adhesome genes.

### 3.2 The adhesome genes that are co-clustered in the cell nucleus are transcriptionally co-regulated

In the previous section we showed that the adhesome genes in adherent cells are mostly active as well as spatially co-clustered in the cell nucleus. To test if the co-clustered adhesome genes are coregulated, possibly to enable them to function together, we next quantified the association between co-clustering and co-regulation. To evaluate this, we quantified the co-regulation of active adhesome genes using Pearson correlation of gene regulatory profiles, containing bulk RNA-seq expression and the 47 cistromic marks described in the previous section. The resulting correlation heatmaps are shown in Fig 2A for all 202 active adhesome genes (left) and a random selection of 202 active non-adhesome genes with equal per-chromosome distribution (right). Rows and columns are arranged using hierarchical clustering with average linkage to better visualize the gene co-regulation. The heatmaps suggest that active adhesome genes are highly co-regulated, with an apparent community structure: in addition to a strongly coregulated core set of adhesome genes (center diagonal block of Fig 2A’s left sub-panel), other smaller adhesome gene sets are tightly coregulated (upper left diagonal blocks).

**Fig 2:**
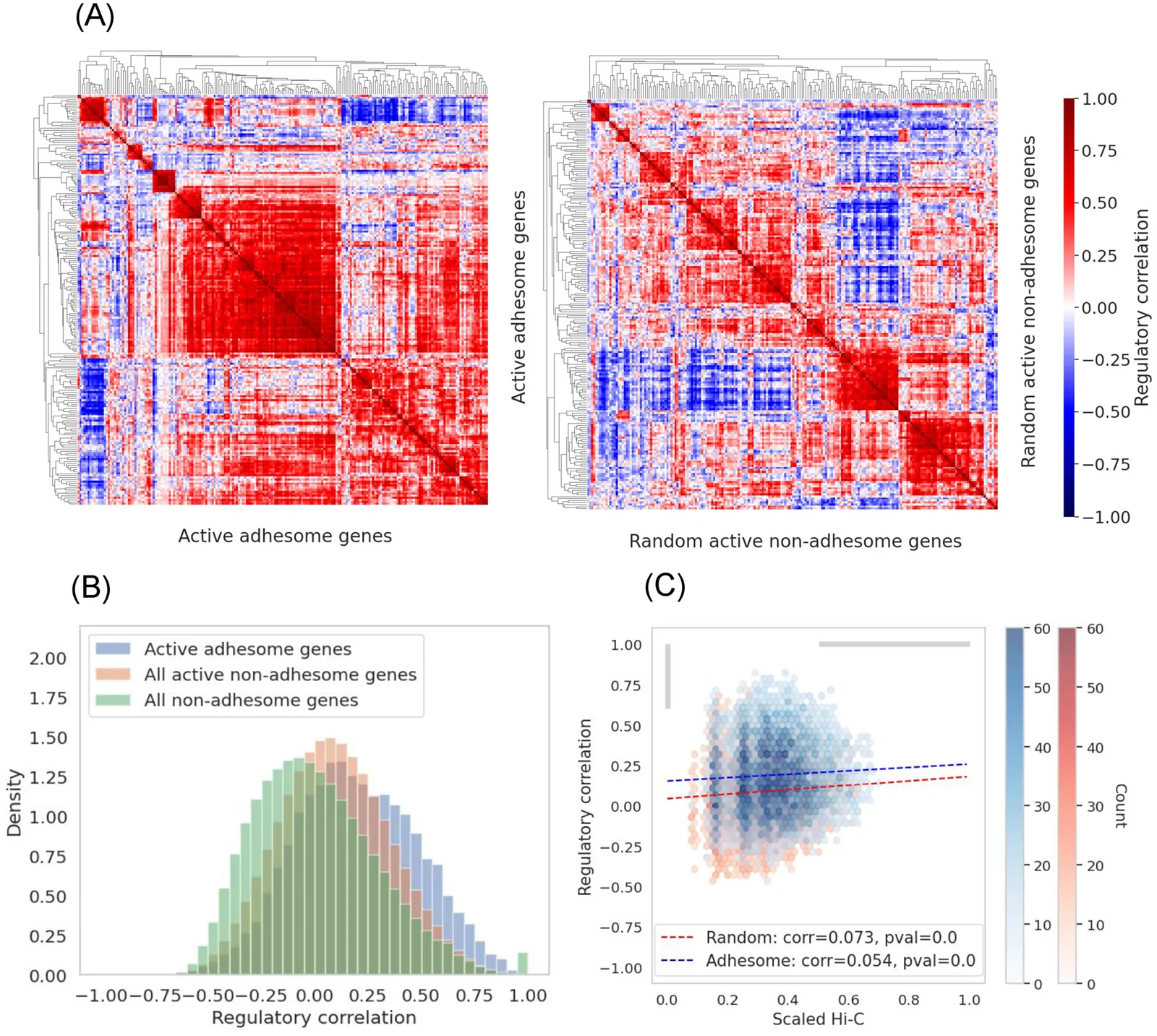
Active adhesome genes are co-regulated in IMR-90 cells. (A) Heatmaps of co-regulation for all active adhesome genes (left) and a set of random active non-adhesome genes (right). Co-regulation is measured using Pearson correlation between a set of 48 regulatory features. Hierarchical clustering based on average linkage was applied to rows and columns for visualization. Overall, active adhesome genes have more similar regulatory profiles compared to other active non-adhesome genes. (B) Histograms of regulatory Pearson correlation for three groups of genes: active adhesome genes (blue), all active non-adhesome genes (orange) and all non-adhesome genes (green). Co-regulation is significantly stronger for active adhesome genes compared to active non-adhesome genes (Wilcoxon Rank-Sums test, *p*-value≤ $3\cdot 10^{-308}$), and similarly for active non-adhesome genes compared to all non-adhesome genes (Wilcoxon Rank-Sums test, *p*-value≤ $3\cdot 10^{-308}$). (C) 2D histogram of scaled Hi-C and regulatory Pearson correlation for active adhesome genes (blue) and a randomly selected set of active non-adhesome genes (red). The two multivariate distributions are significantly different (permutation test *p*-value≤ 0.01, see Methods). For both gene sets, there is a mild, yet significant association between scaled Hi-C and regulatory Pearson correlation (the *p*-values are two-sided with respect to the exact distribution of the empirical Pearson correlation of a bivariate normal sample, see Methods).

In Fig 2B, comparing the distributions of Pearson correlations among active adhesome genes (blue histogram), active non-adhesome genes (orange histogram) and all non-adhesome genes (green histogram) confirmed that active adhesome genes are significantly more co-regulated in IMR-90, when compared to all active non-adhesome genes (*p*-value≤ $3\cdot 10^{-308}$) and all non-adhesome genes (*p*-value≤ $3\cdot 10^{-308}$) under a two-sided Wilcoxon rank-sums test.

This regulatory difference is partially reflected in the proximity of adhesome genes compared to other genes. Fig 2C shows the joint distribution of pairwise max-scaled Hi-C contact frequencies and Pearson regulatory correlations among active adhesome genes (blue 2D histogram) and the random non-adhesome genes (red 2D histogram). Complementary 1D histograms of the marginal distributions and 2D histograms of the joint distributions are reported in S6A–S6D Fig. Hi-C values include both interchromosomal and intrachromosomal contacts, separately max-scaled in order to reduce the prominence of intrachromosomal contacts in the analysis. Indeed, it is known that Hi-C data is biased towards the detection of intrachromosomal contacts, which typically occur at substantially closer spatial distances than interchromosomal contacts [67]. The histograms reveal a weak positive association between scaled Hi-C contacts and regulatory correlation for both gene groups (Pearson correlations 0.054 and 0.073 for adhesome and random non-adhesome genes, respectively; correlation test *p*-value≤ $3\cdot 10^{-308}$). More importantly, the joint distribution for active adhesome genes is significantly shifted upwards compared to random active non-adhesome genes (permutation test *p*-value≤ 0.01, see Methods and S6E Fig). A thresholded network illustrates the strongly contacting and co-regulated adhesome gene pairs (S16 Fig; SR1 Supplementary Results).

### 3.3 Transcription regulators targeting adhesome genes in IMR-90 cells are co-clustered with active adhesome genes

Because transcription factors, cofactors, and chromatin regulators (collectively abbreviated as TRs) play a key role in the regulation and homeostasis of gene expression programs, we next sought to identify the TRs involved in the expression of active adhesome genes in IMR-90 cells. We retrieved data on 5,896,536 TR-target relationships corresponding to 333 TRs and 55,714 target genes in several tissues from hTFtarget [24], a curated database constructed from large-scale ChIP-seq data of human TRs in many conditions, as well as predicted interactions via TR binding site scanning. Among this data, we selected TR-target links present in several normal human lung fibroblast cell lines or samples from tissues, including IMR-90, WI38, AG04450, HPF and NHLF. While the majority of the TR-target data stems from IMR-90 (S17A Fig), also considering the other human lung fibroblast cell lines provided additional TR-target relationships (S17B Fig), as well as four additional TRs (SUMO1, SUMO2, UBE2I and PIAS4) which are present in the WI38 TR-target data yet absent from the IMR-90 data, but active in IMR-90 cells (S17C–S17D Fig). All 31 TRs are shown as magenta dots on the tSNE plot of S18A Fig, along with the adhesome genes (light green dots). We observed that 28 out of the 31 TRs present in these cell lines in hTFtarget are active in IMR-90 according to our regulatory analysis (CHD1, SNAPC4 and SPI1 were discarded as inactive). Among these 28 TRs, 26 target at least one active adhesome gene (SNAPC1 and SNAPC5 only target inactive adhesome genes). In the remainder of this paper, we use “adhesome TR” to refer to a TR (or the gene that codes for this TR) that targets at least one active adhesome gene.

There is heterogeneity in the overall number of active genes targeted by these TRs, with some TRs targeting less than 50 active genes (UBE2I, SNAPC1, SNAPC2, SNAPC4) and other TRs targeting more than 10,000 active genes (LMNB1, MAZ, POLR2A); see Fig 3A. This heterogeneity may reflect both the specificity of a given TR as well as potential missing data from hTFtarget. Interestingly, active adhesome genes tend to be more targeted by the identified TRs than similar random active non-adhesome genes (Wilcoxon Rank-Sum test, *p*-value≤ $2\cdot 10^{-10}$; see S19A Fig), supporting the potential role of the identified TRs in adhesome regulation. Conditional on their total number of active gene targets, we identified 13 TRs whose target genes contained adhesome genes at a significantly higher proportion than expected under a hypergeometric test (family-wise error rate control, FWER, at 5% with Bonferroni correction). These 13 TRs lie outside of the shaded 5% Bonferroni-corrected hypergeometric confidence region in Fig 3B. These include TRs that have been identified to play a role in adhesion in previous research: LMNB1 plays an important role in cellular response to mechanical stress [68], RAD21 regulates the cadherin and cell adhesion pathway [69], and EP300 actively controls cell adhesion [70], among others. The regulators POLR2A, CTCF, MAZ, CEBPB and LMNB1 have the smallest FWER-adjusted *p*-values and target more than 80% of active adhesome genes, suggesting a high level of redundancy and co-regulation (Fig 3C).

**Fig 3:**
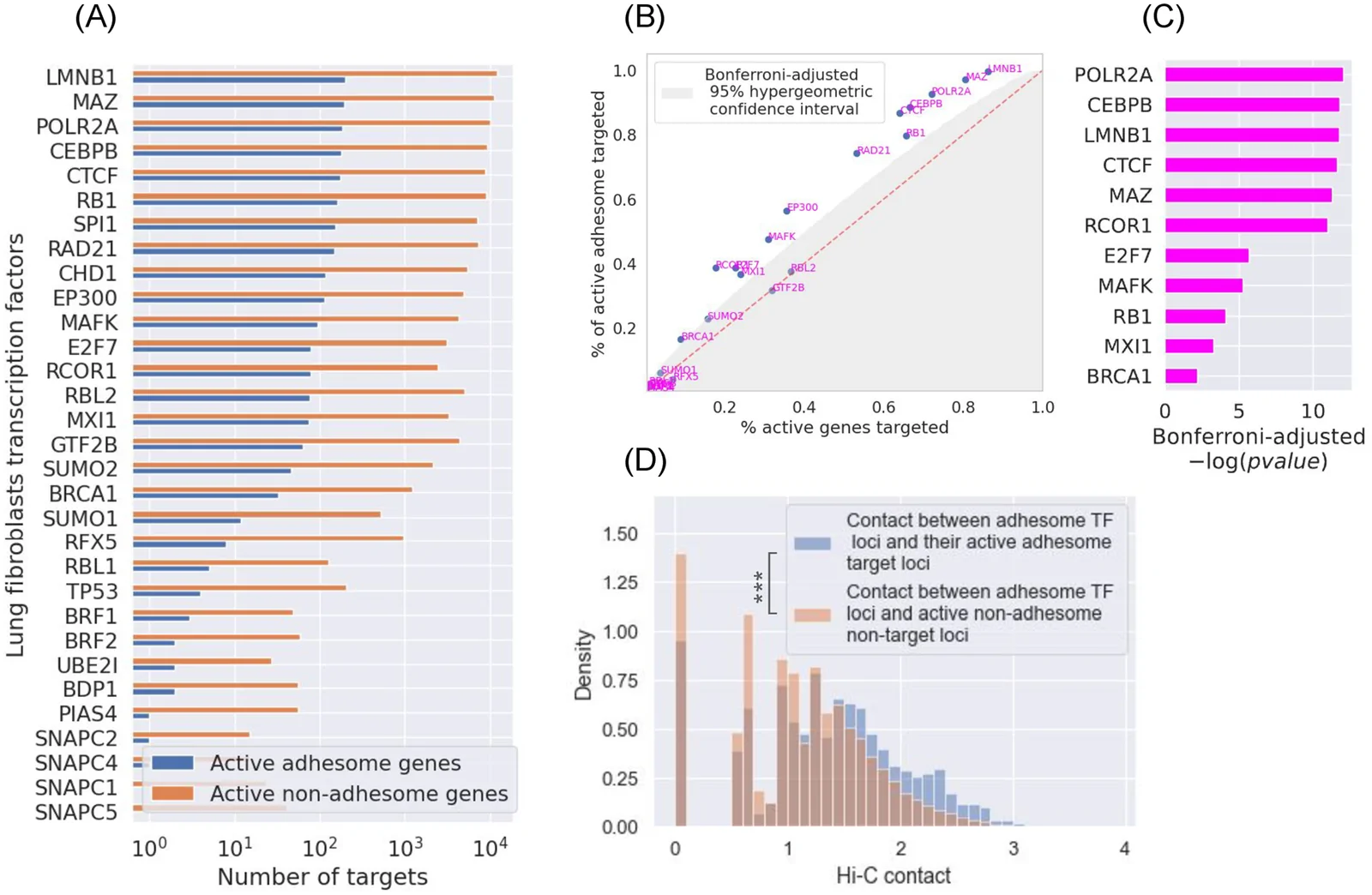
Transcription regulators targeting adhesome genes are co-regulated and spatially clustered in the nucleus of IMR-90 cells. (A) Bar plot of the number of active adhesome gene targets (blue) and active non-adhesome gene targets (orange) for all TRs present in normal human lung fibroblasts (namely the IMR-90, WI38, AG04450, HPF and NHLF cell lines). (B) Scatter plot of the active lung fibroblast TRs that target active adhesome genes showing the proportion of total active genes targeted (x-axis) and the proportion of active adhesome genes targeted (y-axis) for each TR. The shaded region corresponds to the Bonferroni-corrected 95% hypergeometric confidence interval conditional on the number of active genes targeted by each TR. The TRs in this figure correspond to all TRs in (A) except for inactive TRs (see activity classification in Results) and active TRs targeting inactive adhesome genes only (SNAPC1, SNAPC5). (C) Bar plot of Bonferroni-adjusted hypergeometric *p*-values for all significant TRs from (B) at the 95% level. (D) Histograms of interchromosomal Hi-C contact values between all active adhesome TR loci (including loci of both significant and non-significant TRs identified in (B)-(C)) and their active adhesome target loci (blue) as well as all other active non-adhesome, non-target loci (orange). Active adhesome TR loci are significantly closer to their active adhesome target loci compared to the other loci (*p*-value≤ $4\cdot 10^{-90}$, Wilcoxon Rank-Sums test).

The 13 significant adhesome TR genes correspond to 15 distinct 250 kb genomic loci, and the 13 remaining adhesome TR genes correspond to 14 distinct genomic loci. In aggregate, these 29 adhesome TR loci are shown in Fig 3D to be significantly closer to their respective active adhesome target loci than to other active non-adhesome, non-target loci (Wilcoxon Rank-Sums test, *p*-value≤ $4\cdot 10^{-90}$). This observation is also confirmed by an analysis of the 3D proximity of active adhesome target genes for each adhesome TR separately, as shown in S19B–S19C Fig. Most of these TRs exhibit a significant colocalization of their active adhesome gene targets compared to randomly selected active non-target genes on the same chromosomes. This suggests that active adhesome genes and the genes coding for their TRs are spatially close in the nucleus.

We also compared the proportion of adhesome genes targeted by the 13 significant TRs in other cell lines for which sufficient TR-target data was available in hTFtarget (S20A–S20B Fig), including cell lines with adherence properties (A549, HepG2, HEK293) and a non-adherent cell line (GM12878). Interestingly, some of the significant TRs we identified are quite specific to lung fibroblasts, such as LMNB1, RB1, RCOR1, BRCA1, and, to a lesser extent, MAZ and CEBPB. In other cell lines, these TRs tend to target a lower proportion of adhesome genes (S20C Fig). On the contrary, some TRs that do not target adhesome genes in IMR-90 may target a high number of adhesome genes in other cell lines (S20D Fig, bottom rows). Only CTCF targets a high proportion (≥86%) of adhesome genes in all the cell lines under consideration.

We also find that the adhesome TRs forms a densely connected regulatory network (details in Supplementary Results, S17E–S17F Fig, and S18B). This indicates extensive and hierarchical targeting relationships among adhesome TRs in IMR-90 cells.

### 3.4 Clustering analysis identifies interactions of closely localized and co-regulated adhesome genes and their transcription regulators

The above analysis suggests that adhesome genes and the genes coding for their transcription regulators, show strong spatial clustering in the cell nucleus of IMR-90 cells. In order to further dissect the spatial organization of adhesome genes as well as identify non-adhesome genes that are co-clustered and co-regulated with adhesome genes, we ran the Large Average Submatrix (LAS) algorithm [34,56] on the IMR-90 interchromosomal Hi-C matrices to identify specific groups of genes that are spatially clustered in the cell nucleus despite lying on different chromosomes (Methods). Seeded in active adhesome loci, the LAS algorithm greedily identifies contiguous submatrices in which the average contact frequency is significantly higher than expected by chance (see Methods). The output of the LAS algorithm is a list of interchromosomal interactions between genomic regions and is shown in Fig 4A for the example of the Hi-C map between chromosomes 19 and 20.

**Fig 4:**
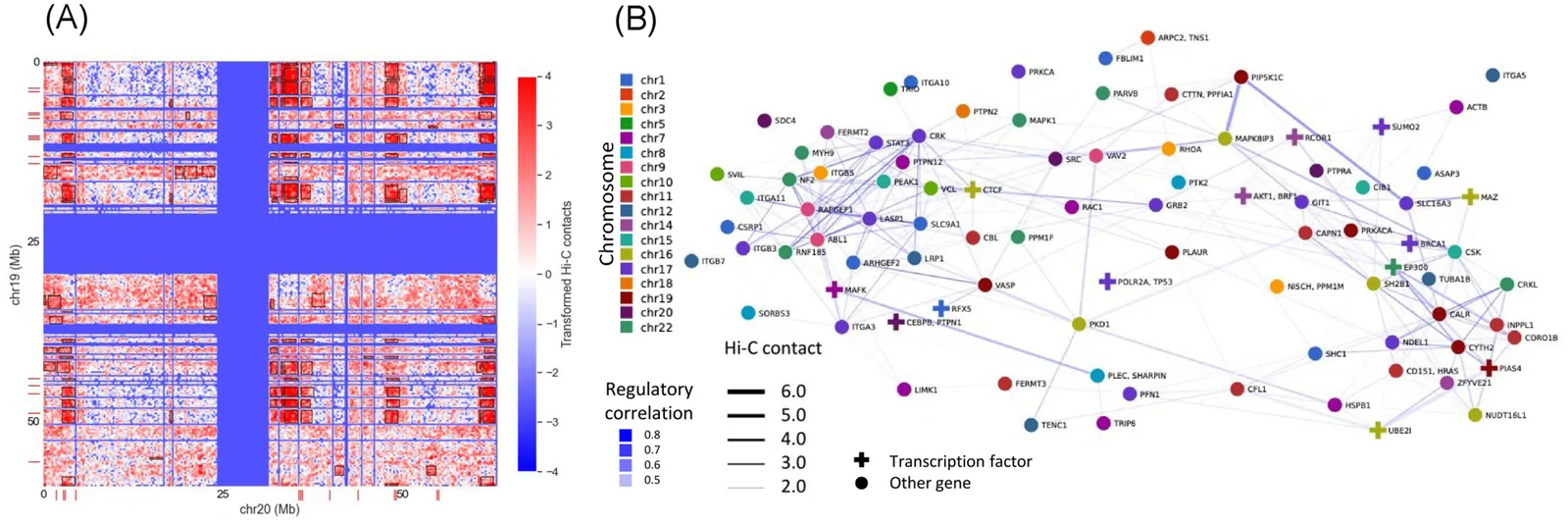
Analysis using the LAS algorithm identifies interacting regions in Hi-C of spatially clustered and co-regulated adhesome genes, their transcription regulators. (A) Heatmap of transformed Hi-C contact frequencies between genomic loci on chromosome 19 and chromosome 20 (frequencies were log-transformed and centerdized using the mean and standard deviation of contact values across the entire genome). Adhesome loci are indicated by red ticks on the x-axis (for chromosome 20) and the y-axis (for chromosome 19). Significant submatrices identified by the LAS algorithm with an average Hi-C contact value higher than 2 are shown in black boxes. (B) Network of adhesome LAS nodes obtained from keeping all edges that are detected by the LAS algorithm and that have a regulatory correlation in the top 15%-percentile. These edges are in the upper right quadrant of S23A Fig, as defined by the red dashed marginal thresholds. Adhesome LAS nodes are colored by chromosome, and a cross indicates that the node contains at least one TR targeting active adhesome genes. Edge width corresponds to the average Hi-C contact value and edge color corresponds to the regulatory correlation.

The LAS algorithm identified 24,809 large average submatrices, 5,540 of which contained active adhesome genes or adhesome TR genes. In the remainder of this work, a set of contiguous genomic loci corresponding to either the rows or the columns of a large average submatrix identified by LAS is called an interacting region; if an interacting region contains at least one adhesome gene or an adhesome TR, it is called an adhesome interacting region. The LAS submatrices projected onto the Hi-C map containing only the active adhesome loci and TR adhesome loci are shown as black boxes in S21A Fig. The adhesome interacting regions contain 1–3 adhesome genes (S21B–S21C Fig). We constructed an adhesome LAS network, where the nodes (which we call adhesome LAS nodes) are adhesome genes, and two adhesome LAS nodes are linked by an edge if they are linked by an LAS submatrix. Adhesome genes belonging to the same chromosome are merged into one adhesome LAS node if they always co-appear in the LAS submatrices. The resulting network contains 118 nodes with 2,250 edges, with an average degree of 38. S22 Fig compares various properties of the adhesome LAS network to a null distribution obtained by building 500 LAS networks based on a random active non-adhesome gene set of the same size (~200 genes), confirming that adhesome genes and adhesome TR genes interact more strongly spatially than expected by chance.

To mitigate some of the noise associated with using Hi-C contact frequencies alone, edges in the adhesome LAS network were weighted (and then pruned) by the regulatory correlation between the two adhesome LAS nodes as follows: for a given pair of adhesome LAS nodes, the regulatory correlation is equal to the average of Spearman correlations between the regulatory profiles of all tuples of adhesome genes such that one of the adhesome genes belongs to the first LAS node and the other gene belongs to the second LAS node. This allowed us to identify chromosome intermingling regions with coordinated activity, which might be controlled by the same set of TRs or epigenetic marks. The scatter plot of interactions between adhesome LAS nodes based on average Hi-C contact value (x-axis) and regulatory correlation (y-axis) is shown in S23A Fig, where red dots correspond to interactions involving at least one adhesome TR. By construction, all edges in the adhesome LAS network have an average Hi-C contact value higher than 2 (S23B Fig). We used a lower threshold on the regulatory correlation (85% quantile of its marginal distribution in the network, see S23C Fig) to prune edges of the network corresponding to weak or negative co-regulation. The resulting network has 92 nodes (100 adhesome genes) and 338 edges (average degree = 7.3) and is displayed in Fig 4B, where nodes are colored by chromosome, edge widths reflect the average Hi-C contact frequency between nodes, and edge colors reflect the regulatory correlation between regions. The network includes 14 adhesome TRs (displayed as crosses), 8 of which were found to be significant in our previous analysis (BRCA1, CEBPB, CTCF, EP300, MAFK, MAZ, POLR2A, RCOR1). Note that MAZ and POLR2A target 100% of genes in the network, while CTCF and CEBPB target more than 85% of genes, EP300, RCOR1 and MAFK target more than 35% of genes, and BRCA1 targets 12% of genes. Interestingly, the maximal cliques of this network involve at most 5 chromosomes (S23D Fig), which is consistent with the observation that the simultaneous interaction of genes from more than five chromosomes is physically unlikely [65,67].

Beyond the adhesome itself, the LAS analysis also identified 2,826 active non-adhesome genes that interact with adhesome regions and have strongly correlated regulatory profiles. These genes are functionally enriched for ubiquitin-proteasome and vesicle-transport functions know to regulate focal adhesions (see S24 Fig; S25 Fig; SR1 Supplementary Results for details). Together, these results indicate that the adhesome LAS clusters constitute interchromosomal neighborhoods containing adhesome genes, their transcriptional regulators, and functionally related machinery.

### 3.5 FISH experiments suggest the spatial proximity of adhesome gene sub-clusters and their transcription regulators

To assess our computational findings of adhesome gene proximity in IMR-90 cells, we analyzed published DNA-MERFISH data [30] using the processing described in Methods. We identified 82 adhesome-associated MERFISH probe loci using the 500-kb mapping window (Methods). These probe loci cover 95 active adhesome genes and adhesome TR genes (i.e., 42%) and are referred to as adhesome probe loci in the remainder of this paper.

The resulting histogram of average cell-normalized Euclidean distances between adhesome probe loci is shown in S4D Fig. Consistent with previous findings [67], proximity, defined as one minus the normalized distance, is positively correlated with the scaled Hi-C frequencies (Pearson correlation of 0.494 for interchromosomal contacts and 0.787 for intrachromosomal contacts; see S4E Fig). For comparison, we considered 82 chromosome-matched active non-adhesome probe loci (Methods). The resulting distance heatmaps are shown in S26 Fig confirming that adhesome probe loci are closer compared to non-adhesome probe loci; this is even more apparent if we restrict the heatmaps to probe loci that are less than 100 kb away from adhesome genes (Fig 5A). The histograms of average cell-normalized Euclidean distances in Fig 5B confirm that active adhesome probe loci are significantly closer compared to all other active non-adhesome probe loci (Wilcoxon rank-sums test, *p*-value≤ $3\cdot 10^{-308}$). These findings remained valid when we restricted the analysis to only MERFISH probe loci that overlap with active adhesome genes (19 probes covering 19 active adhesome genes), as shown in S27 Fig.

**Fig 5:**
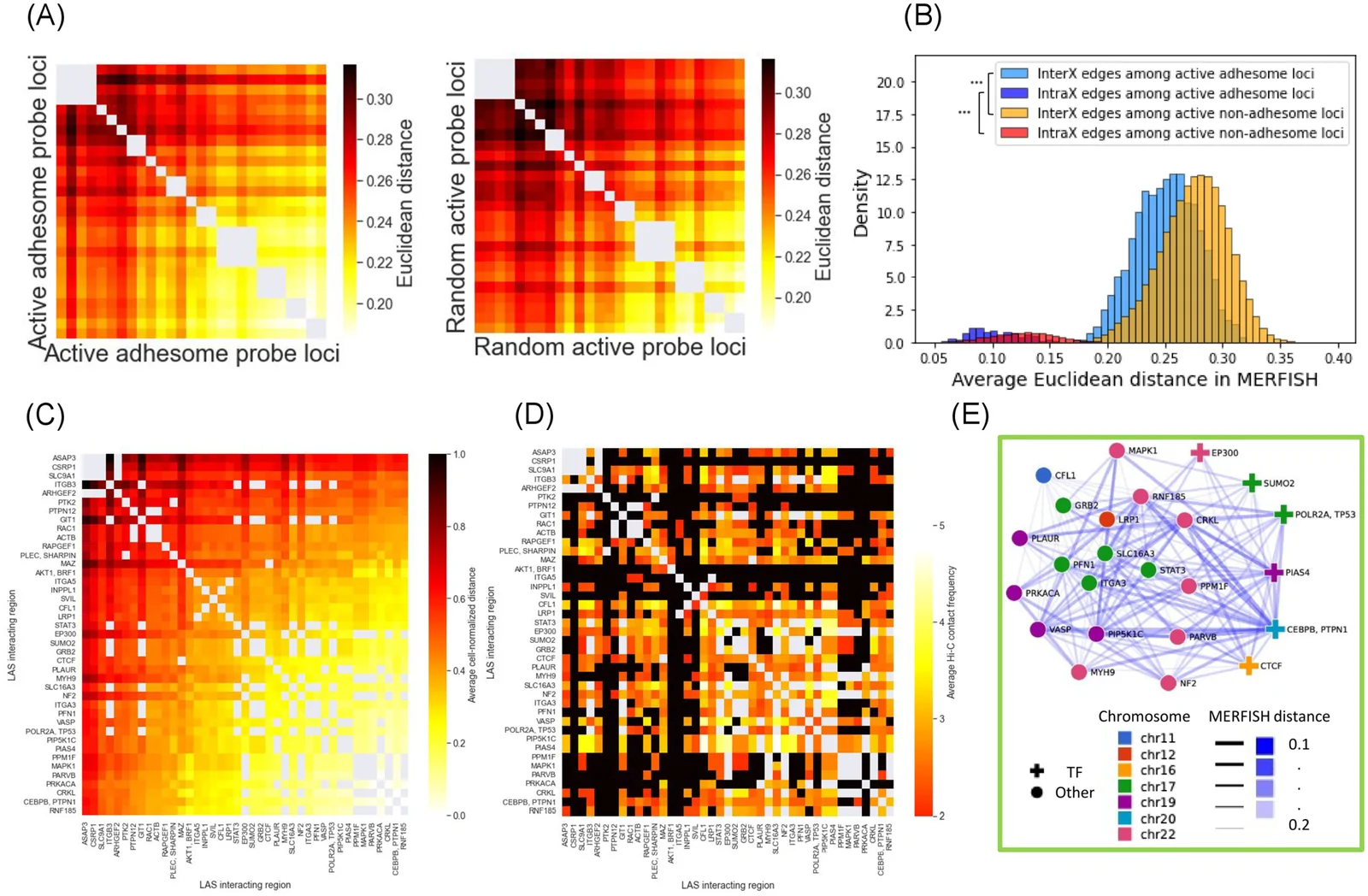
DNA-MERFISH data suggests the proximity of the identified co-regulated adhesome genes and their transcription regulators. (A) Top: Heatmap of mean cell-normalized Euclidean distances between probe loci within 100 kb of active adhesome or transcription regulator genes. Bottom: Corresponding heatmap for chromosome-matched active non-adhesome probe loci. Mapping, normalization, and control sampling are described in Methods; the 500-kb comparison is shown in S26 Fig. (B) Histograms of average Euclidean distance between active adhesome probe loci and between all active non-adhesome probe loci. (C) Heatmap of average Euclidean distance between nodes in the adhesome LAS network of Fig 4B. Only nodes containing at least one active adhesome probe locus are shown. Entries corresponding to nodes on the same chromosome are colored in grey. Rows and columns are ordered using hierarchical clustering with single linkage. (D) Heatmap of average Hi-C contact values between nodes in the adhesome LAS network of Fig 4B. Only nodes containing at least one active adhesome probe locus are shown. Entries corresponding to nodes on the same chromosome are colored in grey; entries corresponding to interactions that were not identified by LAS are set to 0 and colored in black. Rows and columns are ordered as in (C). (E) Subnetwork of adhesome LAS nodes corresponding to the lower right quadrant of (C) and (D) (highlighted in green). Nodes are colored by chromosome and represented with a cross if they contain at least one active adhesome transcription regulator.

The MERFISH data also allows us to validate some of the interchromosomal interactions between active non-adhesome genes and active adhesome genes. In the previous section, 2,826 active non-adhesome genes were found to cluster and to be co-regulated with 135 active adhesome genes. We mapped these 2,961 genes to the closest MERFISH probe within 100 kb upstream or downstream on the genomic sequence. This resulted in 135 active non-adhesome genes (associated with 61 probes) and 17 active adhesome genes (associated with 16 probes); all other genes have no associated probe locus.

The distribution of MERFISH distances between these gene pairs is shown in S28A–S28B Fig in red. Comparing this distribution to the distribution of distances between active adhesome MERFISH probes across LAS clusters (S28A Fig) and within the same LAS cluster (S28B Fig) confirms that the identified active non-adhesome genes are highly co-clustered with the active adhesome genes they are predicted to interact with (Wilcoxon Rank-Sums test, *p*-value≤ $2\cdot 10^{-29}$). We visualized this distance information by creating a bipartite network in which active adhesome genes are linked to active non-adhesome genes if the distance between these genes falls in the lower quartile of the distance distribution; after discarding isolated nodes, we obtained a bipartite network with 52 nodes (10 active adhesome genes and 42 active non-adhesome genes) and 50 edges (S28C Fig). Several of these non-adhesome genes have known adhesion-related functions (Supplementary Results). Finally, we used the processed MERFISH data to validate the interactions between nodes of the adhesome LAS network. The scaled Euclidean distances between nodes in the adhesome LAS network are shown in S29 Fig, where the distance between two adhesome LAS nodes is defined by averaging the distances between all DNA-MERFISH probes associated to the first LAS node and all DNA-MERFISH probes associated to the second LAS node. Nodes without an associated DNA-MERFISH probe are represented as grey rows/columns. Interactions with very low Euclidean distance (darkest entries of the heatmap) correspond to intrachromosomal interactions, which we excluded from the subsequent analysis because of the focus of this work on interchromosomal interactions. The resulting MERFISH Euclidean distance matrix among adhesome LAS nodes with associated MERFISH probes is shown in Fig 5C. Rows and columns are ordered using hierarchical clustering with single linkage (cophenetic correlation ~0.4, see S5A Fig). The corresponding matrix of average transformed Hi-C contact frequencies is shown in Fig 5D. Entries corresponding to edges that were not identified by the LAS algorithm are set to 0 and shown in black. It is apparent by considering the heatmaps in Fig 5C–5D that the Hi-C and MERFISH adhesome networks have a common core of 24 tightly interacting adhesome LAS nodes (in the lower right quadrant). S5B–S5D Fig confirm that these adhesome LAS nodes are also co-regulated. The network of these tightly interacting adhesome LAS nodes is displayed in Fig 5E; it contains 24 nodes (corresponding to 26 adhesome genes on 7 chromosomes) and 169 edges (average degree = 14.1). Nodes are colored by chromosome and edges are colored and sized according to MERFISH Euclidean distance. The genes in the network mostly code for adaptor proteins (CRKL, GRB2, NF2, PARVB), or are involved in actin regulation (CFL1, VASP, MYH9, PFN1) and adhesion receptors (LRP1, ITGA3, PLAUR). The network also contains 7 adhesome TRs, including 4 significant adhesome TRs: POLR2A, CTCF and CEBPB, which target more than 88% of genes in the subnetwork, and EP300, which targets 50% of genes in the subnetwork. Protein-protein interaction (PPI) data retrieved from the physical STRING v11 interactome [53] shows that some of these proteins also interact physically, such as ITGA3-PLAUR, CTCF-EP300, and MYH9-CTCF (S5E Fig).

To further strengthen our conclusions from the analysis of MERFISH data on genes close to (~100KB downstream or upstream) adhesome genes, we directly labeled the loci of 26 core adhesome genes and 29 negative controls (gene list provided in S3 and S4 Files in human dermal fibroblasts using DNA-FISH probes conjugated with fluorescent dyes. The 26 genes targeted by DNA-FISH correspond to the complete gene set represented in the Hi-C/MERFISH core network (Fig 5E; S3 File). The negative control genes listed in S4 File correspond to transcriptionally active non-adhesome genes, which were selected uniformly at random from all active loci that are not interacting with adhesome loci (i.e., active loci which are not connected to adhesome loci in the genome-wide LAS network). Note that each probe labels multiple copies of its target gene, and thus some degree of non-colocalization is expected due to allelic variation and stochastic chromatin organization. Despite this, we observed proximally distributed adhesome gene loci in contrast to the negative controls. The DNA-FISH gene sets were not constrained to be equally distributed across chromosomes. As shown in S3 and S4 File, and the karyogramm in S30F Fig, despite adhesome and negative control genes being distributed across 7 and 14 chromosomes respectively, we find that there is no significant difference between the distribution of the fraction of adhesome and negative control genes across different chromosomes (S30E Fig).

Representative z-projected confocal fluorescence images (Fig 6A) show cell nuclei hybridized with these DNA-FISH probes, highlighting both the adhesome genes and the selected negative control genes, as well as phosphorylated RNA Pol2 (pRNA POL2)-labeled regions. We then segmented the loci and quantified their association with pRNA Pol2 (see Methods and S31 Fig). Representative images overlayed with segmented loci masks in Fig 6B and S30A, S30B Fig show that the segmentation algorithm provides robust labels of gene loci. Using the intensity quantification of pRNA Pol2 in the vicinity (500 nm) of core adhesome gene loci, we find that the adhesome genes are significantly enriched with pRNA Pol2 (Fig 6C). By comparing the mean pairwise distances within the core adhesome gene loci and within the negative control genes, we find that the core adhesome genes are distributed closer together than the negative control genes (Fig 6D), which are more dispersed throughout the nucleus (S30C Fig). The radial distribution function calculated with respect to the nuclear geometric center and gene centroids, respectively, indicates stronger local clustering of adhesome gene loci (S30G, S30H Fig). We also observed that the number of distinct segmented loci of negative control genes is closer to the theoretical value of 2 × Gene count as compared to the adhesome genes. This indicates that there is increased clustering of adhesome gene loci at the length scale of the diffraction limit (S30D Fig). Taken together, the experimental results indicate that the adhesome genes show higher spatial proximity and transcriptional activity.

**Fig 6:**
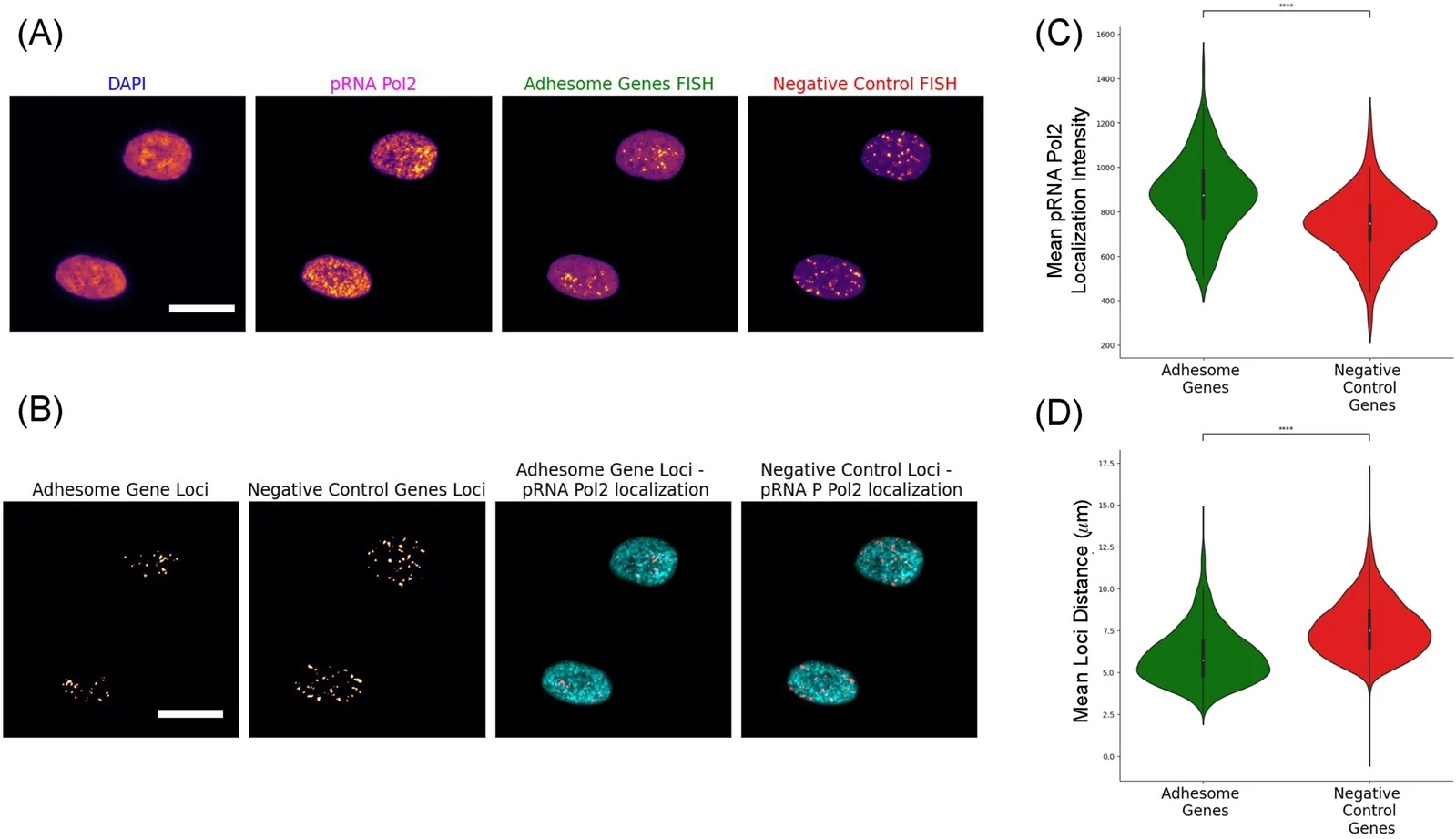
Experiments to suggest spatial sub-clustering and expression of adhesome genes. (A) Representative confocal images of human skin fibroblast (GM09503) with nuclear staining and pRNA POL2, and the adhesome gene and negative control gene loci stained with DNA FISH probes. (B) Representative images of the segmentation of adhesome gene loci, negative control gene loci, and pRNA Pol2 (cyan) localization with the adhesome (red) and negative control gene loci (red). (C) Violin plot showing mean pRNA Pol2 intensity of regions localized around the core adhesome and the negative control gene loci. (D) Violin plot showing mean inter loci distance for the core adhesome and the negative control gene sets; p-values (Mann-Whitney U test/Wilcoxon rank-sum test): **** ≤ 0.0001, *** ≤ 0.001, ** ≤ 0.01, * ≤ 0.05. Scale bars: (A) and (B) 20μm.

## 4 Discussion

Cells have evolved to organize their receptors on the plasma membrane for signal sensing [1–6]. In addition, the genetic material is packed within the cell nucleus to encode the various cellular proteins [20–23]. In recent years two aspects of spatial clustering have been identified, one at the level of receptors for optimized signal sensing, and the other at the level of genes for optimized transcriptional regulation. While these two layers of clustering may appear independent of each other, we hypothesized that there may exist a coupling between the two spaces. The main premise of this hypothesis is due to cells having to detect the extracellular signals through their receptors and needing to transcriptionally reinforce the sensing machinery for rapid adaptation [71]. Spatial clustering of the corresponding genes could contribute to their coordinated regulation; a role in rapid cellular response remains to be tested.

To test our hypothesis, we analyzed publicly available genomic datasets in IMR-90 cells to examine the spatial arrangement of genes encoding adhesome proteins and their regulators. When interrogating these Hi-C maps, we identified that the adhesome genes were spatially clustered, with transcriptionally active adhesome genes being co-clustered. We next tested whether genes encoding the transcriptional regulators of active adhesome genes were spatially adjacent to their target loci. Upon matrix sensing, various downstream signal processes are triggered including some of the adhesome proteins serving as transcription factors, cofactors, and chromatin regulators. We found that genes encoding many of these transcription regulators are co-localized within the adhesome gene clusters. Extending our analysis to identify non-adhesome genes that are co-localized within the adhesome gene clusters, we identified genes of the ubiquitin-proteasome system – a system known to be involved in the regulation of focal adhesions [72]. In order to test if the adhesome gene clusters identified using population-level Hi-C maps are physically close-by in single cells, we took advantage of recent DNA-MERFISH data in IMR-90 cells, which includes probes associated with adhesome genes [30]. To provide additional validation, we labelled a subset of adhesome genes identified in our analysis along with control genes using DNA-FISH. We indeed find that the adhesome gene loci are spatially sub-clustered and show higher local phosphorylated RNA Pol2 signal. Together with the analysis of the MERFISH dataset, these experiments validate at the single-cell level that many of the adhesome genes and their transcriptional regulators are spatially clustered. We note, however, that the clustered organization of adhesome genes could also arise as a secondary consequence of transcriptional activity, for example, through local condensation of RNA Pol2 and associated factors. Although our analyses show increased transcription and RNA Pol2 occupancy at adhesome loci, our current data do not allow us to disentangle whether spatial gene clustering is a cause or a consequence of elevated transcriptional activity. Collectively, our results highlight a link between protein clustering on the membrane and gene clustering in the cell nucleus. Typical matrix sensing involves the clustering of receptors in the first few minutes, followed by adhesome maturation and cellular responses in the next tens of minutes, which is then followed by cell behavioral responses in subsequent hours to days [73]. Whether similar spatial organization contributes to other receptor-mediated responses to microenvironmental signals remains an open question.

## Supporting Information

S1 Fig Function and structure of the adhesome. (A) Gene Set Enrichment Analysis of intrinsic and associated adhesome components using the 2018 Gene Ontology database for biological processes. (B) Network of interactions among adhesome proteins (236 nodes, 1501 edges, average degree = 12.7, density = 0.0541). Nodes are colored in blue if they are intrinsic adhesome proteins, and in red if they are associated adhesome proteins. Nodes are grouped by functional categories. Links between nodes are colored based on the type of interaction. An interactive version of the network in  .html format is provided as S2 File.
(PDF)

S2 Fig TAD calling. (A) TAD calling pipeline shown for chromosome 21 at resolution 25 kb with Knight-Ruiz normalization. The Arrowhead software (see Methods section) is applied to the normalized intrachromosomal Hi-C contact matrix of chromosome 21 at a given resolution (1st from top), resulting in a list of potentially overlapping and nested TADs (2nd from top). This TAD list is then processed to remove nested TADs and merge overlapping TADs (3rd from top). Most TADs are disjoint, yet a minority of TADs are nested and only very few TADs are overlapping (last from top). (B) Number of TADs called per chromosome before and after post-processing for TAD lists called by Arrowhead at different resolutions (5 kb, 10 kb, 25 kb), with and without Knight-Ruiz normalization. (C) Distribution of TAD length before and after post-processing for TAD lists called by Arrowhead at different resolutions (5 kb, 10 kb, 25 kb), with and without Knight-Ruiz normalization. (D) Number of base pairs covered per chromosome by processed TADs for TAD lists called by Arrowhead at different resolutions (5 kb, 10 kb, 25 kb), with and without Knight-Ruiz normalization.
(PDF)

S3 Fig TAD quality. (A) Measure of Concordance (MoC) heatmap for TAD lists called by Arrowhead at different resolutions (5 kb, 10 kb, 25 kb), with and without Knight-Ruiz normalization. An MoC of 1 indicates that 2 TAD lists are identical, while an MoC of 0 indicates that 2 TAD lists are non-overlapping (see Methods). (B) Negative log-*p*-value of the structural protein profiles (see Methods) of CTCF, RAD21, and SMC3 for TAD lists called by Arrowhead at different resolutions (5 kb, 10 kb, 25 kb), with and without Knight-Ruiz normalization. (C) Number of TADs per chromosome significantly enriched for either transcriptional activating (H3K36me3) or repressing (H3K27me3) histone marks for TAD lists called by Arrowhead at different resolutions (5 kb, 10 kb, 25 kb), with and without Knight-Ruiz normalization. Enrichment is estimated as the log10-ratio between the ChIP-seq signals of H3K36me3 and H3K27me3 and compared with a null distribution derived from random permutations (see Methods).
(PDF)

S4 FigNormalization of Euclidean distances between DNA-MERFISH probes.(A) 3D scatterplot of all 959 DNA-MERFISH probes in the nucleus of a given cell (cell ID 1788). The shape of the nucleus is approximately an ellipsoid with major axis length ~20 μm, which is consistent with the expected nucleus size of IMR-90 cells. (B) Histogram of the maximum distance between pairs of DNA-MERFISH probes for 3,668 cells before downstream filtering. The maximum distance for each cell is used to normalize distances between probes in that cell. (C) Histogram of the number of cells in which a pair of DNA-MERFISH probes is found. The distance metric we use in the main text, i.e., the average cell-normalized Euclidean distance between two probes, corresponds to normalizing the distance between two probes in a cell by the maximum probe-probe distance in the cell in which the two probes are found, then taking the average over all cells that contain these two probes. The histogram shows that this average is taken on at least 2,800 cells and should thus enjoy a high statistical accuracy. (D) Histogram of average cell-normalized Euclidean distances between adhesome DNA-MERFISH probes. Intrachromosomal probe-probe distances are shown in blue, while interchromosomal probe-probe distances are shown in orange. (E) Scatter plot of max-min scaled Hi-C contacts and cell-normalized DNA-MERFISH Euclidean proximity (defined as (1-Euclidean distance)) for interchromosomal adhesome gene pairs (blue, *p*-value $<3\times 10^{-308}$, see ref [54].) and intrachromosomal adhesome gene pairs (orange, *p*-value $<3\times 10^{-308}$).(PDF)

S5 Fig Joint analysis of DNA-MERFISH data, Hi-C data, regulatory data, and protein-protein interaction data identifies a cluster of highly co-localized and co-regulated adhesome genes. (A) Bar plot of cophenetic correlation for different linkage functions applied to the average cell-normalized DNA-MERFISH Euclidean distance matrix between adhesome LAS nodes having at least one associated DNA-MERFISH probe. (B) Heatmap of average cell-normalized DNA-MERFISH Euclidean distance between adhesome LAS nodes having at least one associated DNA-MERFISH probe. Rows and columns are ordered using hierarchical clustering with single linkage. Single linkage produces a dendrogram that is reasonably faithful to the original Euclidean distances (cophenetic correlation 0.4, see (A)) and yields an interpretable ordering of the heatmap. Entries of the heatmap that correspond to self-interactions and intrachromosomal interactions are shown in grey. (C) Heatmap of average transformed Hi-C contact frequency between adhesome LAS nodes having at least one associated DNA-MERFISH probe. Rows and columns are ordered as in (B). Entries of the heatmap that correspond to self-interactions and intrachromosomal interactions are shown in grey. Entries corresponding to interactions that were not identified by the LAS algorithm are shown in black. (D) Heatmap of average Spearman regulatory correlation between adhesome LAS nodes having at least one associated DNA-MERFISH probe. Rows and columns are ordered as in (B). (E) Heatmap of protein-protein physical interaction confidence score between genes in adhesome LAS nodes having at least one associated DNA-MERFISH probe. Rows and columns are ordered as in (B). Protein-protein physical interactions are retrieved from the physical STRING network. The confidence score for the interaction of two proteins is based on the number and type of sources reporting this interaction. A score of 0 indicates that two proteins are not known to physically interact.
(PDF)

S6 FigJoint distribution of scaled Hi-C contact values and regulatory correlation among active adhesome genes in IMR-90 cells.(A) Histogram of scaled Hi-C contact values between active adhesome genes (blue) and a random set of active non-adhesome genes (red). There are significantly more contacts between adhesome genes compared to random non-adhesome genes (Wilcoxon Rank-Sums test, *p*-value = 1e-85). (B) Histogram of regulatory Pearson correlation (based on the 48 regulatory features of S10A Fig) between active adhesome genes (blue) and a random set of active non-adhesome genes (red). There is significantly more coregulation among adhesome genes compared to random non-adhesome genes (Wilcoxon Rank-Sums test, *p*-value $<3\times 10^{-308}$). (C) 2D histogram of scaled Hi-C contact values and Pearson regulatory correlation for active adhesome genes. The dashed box shows the thresholds used to build the network in S16 Fig. (D) 2D histogram of scaled Hi-C contact values and Pearson regulatory correlation for random active non-adhesome genes. (E) Simulated null distribution for the permutation test used to assess the difference between the joint distribution of scaled Hi-C contact values and Pearson regulatory correlation for the two gene groups of interest: active adhesome genes (blue, see (C)) and the randomly selected set of active non-adhesome genes (red, see (D)). The test is a two-sample test based on the number of nearest neighbor type co-incidences 3. The red vertical dashed line corresponds to the actual value of the statistic. (F) Zoom into the region around the dashed box of (C).(PDF)

S7 FigClustering of transformed Hi-C contact frequencies using LAS algorithm.(A) Histogram of non-zero log-transformed and centerdized Hi-C contact values in the whole genome. Centerdization was performed using the mean and standard deviation of the non-zero contact values across the whole genome. (B) Histogram of negative log10-transformed *p*-values of submatrices identified by the LAS algorithm [34] in all interchromosomal Hi-C contact matrices. The *p*-value threshold of 10^–15^ used for signal detection is represented as a red dashed vertical line. This *p*-value threshold controls the FWER at≤0.0001. (C) Histogram of average transformed Hi-C contact values in all LAS submatrices. The Hi-C threshold of 2 used to discard low-average submatrices is represented as a red dashed vertical line. (D) Heatmap of transformed Hi-C contact frequencies between genomic loci on chromosome 19 and chromosome 20. Adhesome loci are indicated by red ticks on the x-axis (for chromosome 20) and the y-axis (for chromosome 19). Significant submatrices identified by the LAS algorithm are shown in black boxes. (E) Same as (D), except that only significant submatrices identified by the LAS algorithm with an average Hi-C contact value higher than 2 are shown in black boxes.(PDF)

S8 Fig List of adhesome genes and their activity. Intrinsic and associated adhesome genes from ref [43]. colored by activity level (obtained from hierarchical clustering of the combined set of 48 regulatory features as explained in the main text). A searchable version in XLSX format is provided as S1 File.
(PDF)

S9 Fig (A) Histogram of the functional categories for all nodes in the network of S1B Fig. (B) Histogram of the interaction types for all edges in the network of S1B Fig. (PDF)

S10 Fig Genome-wide regulatory features in IMR-90 cells. (A) Table describing the 48 regulatory features used in this study, including bulk RNA-seq and 47 cistromic marks (TR and histone ChIP-seq, DNAse-seq). The table also reports the source and accession ID of each data set. Likely annotations on the regulatory activity of each mark are provided when available in the literature (see ref [74]. and Methods). (B) Genome-wide heatmap in IMR-90 cells of the 48 regulatory features in (A). Genes (rows) and regulatory features (columns) were clustered using hierarchical clustering with average linkage and Pearson correlation metric. The feature matrix was normalized and scaled prior to clustering (Methods). The labels of two typical active marks (RNA-seq, POLR2A ChIP-seq) are colored in green and the labels of two typical repressive marks (H3K9me3, H3K27me3 ChIP-seq) are colored in red.
(PDF)

S11 FigActivity of adhesome genes in GM12878.(A) Heatmap of 100 regulatory marks used to determine the activity of genes in GM12878. Genes (rows) and regulatory marks (columns) were clustered using hierarchical clustering with average linkage and Pearson correlation metric. The feature matrix was normalized and scaled prior to clustering (Methods). (B) tSNE representation of all 25,959 genes based on the 100 GM12878 regulatory marks. Orange dots correspond to inactive genes (9,107 genes, as determined by the clustering in (A)), blue dots correspond to active genes (10,984 genes), and green dots correspond to adhesome genes. Out of the 222 adhesome genes studied (green dots), 172 are active (in contrast to 202 in IMR-90 cells). (C) Histogram of bulk RNA-seq expression in GM12878 for all active genes (blue) and all inactive genes (orange). Active genes have a significantly higher expression than inactive genes (*p*-value $<3\times 10^{-308}$, Wilcoxon rank-sum test). The embedded graph in the upper right corner corresponds to the histogram of RNA-seq expression in GM12878 for all active adhesome genes and all inactive adhesome genes.(PDF)

S12 Fig Active adhesome genes intersect with B Cell Receptor signaling pathways in GM12878. (A) Table of signaling pathways [62–64] related to the B Cell Receptor (BCR) signaling pathway. We intersect genes in each pathway with the active genes in GM12878 (as determined in S11 Fig) to obtain the corresponding number of active pathway genes. (B) Venn diagram of four gene lists: active adhesome genes in GM12878 (blue), active BCR signaling genes (yellow), active actin cytoskeleton regulation genes (green), and active PI3-AKT signaling pathway genes (red). The Venn diagram was drawn using Venny [75].
(PDF)

S13 FigActive adhesome genes with high Hi-C contacts.(A) Distribution of processed interchromosomal Hi-C contact values among active adhesome gene loci. A lower threshold materialized by the dashed red vertical line at 2.74 (99th percentile) is applied to select the strongest loci interactions, which are shown in the network in B. (B) Subnetwork of active adhesome gene loci, colored by chromosomal location and labeled with the active adhesome genes they harbor. Only non-isolated nodes engaging in high Hi-C contacts (Hi-C≥ 2.74, see A) are shown in the plot; edges are sized according to the corresponding Hi-C contact value. The network contains 82 active adhesome loci (74 adhesome genes) and 321 edges (average degree = 7.8). (C) Gene set enrichment analysis of active adhesome genes present in the subnetwork in B using the GO Biological Process 2018 gene sets. Only GO terms with FDR-corrected enrichment *p*-value smaller than 0.0001 are reported.(PDF)

S14 FigTAD-based interchromosomal proximity analysis.(A) 134 TADs out of 4,443 TADs called by Arrowhead (at resolution 5 kb with Knight-Ruiz normalization) contain at least one active adhesome gene (left). The number of active adhesome genes either in TADs or not in TADs per chromosome is shown in the right sub-panel. (B) Hi-C contact map between active adhesome TADs (top) and random active non-adhesome TADs (bottom). Each row/column corresponds to a TAD. The contact value between two TADs is obtained by averaging the Hi-C contact values associated with these TADs. TADs are grouped by chromosomal location, as shown by the side color bars. The selection of random active adhesome TADs is explained in D and E. (C) Distribution of TAD length for all 4,443 TADs called by Arrowhead at resolution 5 kb with Knight-Ruiz normalization. TADs are stratified into buckets of length 50 kb. These buckets are used to sample background TADs on a per-chromosome basis. (D) Distribution of length-normalized TAD gene expression for all 4,443 TADs called by Arrowhead at resolution 5 kb with Knight-Ruiz normalization. TADs are stratified into buckets of size 0.2. These buckets are used to sample background TADs on a per-chromosome basis. (E) Distribution of interchromosomal and intrachromosomal contacts between active adhesome TADs and between random TADs. Random TADs were selected such that they are similar in size to adhesome TADs, they are similarly distributed over the chromosomes, and they have a similar expression level. The distribution of interchromosomal contacts between active adhesome TADs significantly dominates the distribution of interchromosomal contacts between random TADs (Wilcoxon Rank-Sum test, *p*-value $<5\times 10^{-124}$).(PDF)

S15 Fig Intrachromosomal proximity analysis. (A) Localization of active and inactive adhesome genes on the chromosomes (using the hg19 reference genome). (B) Top: Distribution of 1D genomic distances (in bp) between active adhesome genes and between a set of random active genes located on the same chromosomes; the distance between 2 genes (located on the same chromosome) is defined as the minimum distance between |start2-end1| and |start1-end2|. Bottom: Number of active adhesome genes per active adhesome TAD, using TADs called by Arrowhead at resolution 5 kb with Knight-Ruiz normalization. (C) Top: Distribution of intrachromosomal Hi-C contacts among active adhesome gene loci and random active non-adhesome loci located on the same chromosomes. Bottom: Subnetwork of active adhesome loci whose intrachromosomal Hi-C contact values are above 7 in the histogram above; only non-degenerate connected components are shown. Adhesome gene loci are colored by their chromosomal location and labeled with the adhesome genes they harbor. Contiguous loci are linked by black edges, while non-contiguous loci are linked by cyan edges.
(PDF)

S16 Fig Co-regulatory network of active adhesome genes, where nodes have been grouped by chromosome (dashed ellipses). The network skeleton is obtained by lower-thresholding the pairwise Hi-C contact values (scaled between 0 and 1) at 0.5 and lower-thresholding the pairwise regulatory Pearson correlation at 0.6, which corresponds to the grey-shaded areas of the x- and y-axes in main Fig 2C. Network weights are proportional to the corresponding pairwise regulatory Pearson correlation. Genes are colored and grouped by chromosome.
(PDF)

S17 FigTR-target data in normal human lung fibroblast cell lines.(A) Bar plot of the number of TR-target links from the hTFtarget data set in five human lung fibroblast cell lines (AG04450, HPF, NHLF, WI38, IMR-90). Only targets that are active in IMR-90 are considered. (B) Set overlap plot for the TR-target relationships in cell lines AG04450, HPF, NHLF, WI38, and IMR-90. Only targets that are active in IMR-90 are considered. While most identified TR-target interactions come from the IMR-90 data, other human lung fibroblast cell lines contribute a few additional TR-target interactions that are not present in the IMR-90 data. (C) Table of TRs present for all normal human lung fibroblast cell lines. Red font indicates inactive genes, purple font indicates TRs that are not present in IMR-90, and brown font indicates TRs that target inactive adhesome genes. (D) Histogram of z-scored RNA-seq expression for all genes in IMR-90. The expression of SUMO2, SUMO1, UBE2I and PIAS4 (transcription regulators found in WI38 only) are represented with colored vertical dashed lines. (E) Histograms of the number of nodes (top) and the number of edges (bottom) in 500 random TR regulatory networks (only lung fibroblast TR-target relationships in hTFtarget [24] are considered). Each random network is obtained from the active TRs that target a set of randomly selected genes (with the same cardinality as the set of active adhesome genes). The red dashed vertical lines report values of nodes (*p*-value = 0.066) and edges (*p*-value = 0.028) corresponding to the network of active adhesome TRs. (F) Bar plot of Katz centrality of all active lung fibroblast TRs in the network of S18B Fig. The parameter alpha of Katz centrality was chosen to be 1/s-0.01, where s is the largest eigenvalue (in magnitude) of the adjacency matrix of the network in S18B Fig. Bars are colored in red if they correspond to significant TRs from main Fig 3C, otherwise they are colored in blue. There is a significant enrichment of significant TRs at the top of the list (XL-minimal hypergeometric test [76], *p*-value $<6\times 10^{-4}$ and optimal cutoff value at 17, which corresponds to BRCA1). The parameters X and L were chosen to be 10% of the number of significant TRs and 150% of the number of significant TRs, respectively.(PDF)

S18 Fig (A) tSNE representation of all 25,959 genes based on 48 IMR-90 regulatory features. Orange dots correspond to inactive genes (as determined by the corresponding expression of regulatory marks), and blue dots correspond to active genes. Adhesome genes are shown in green, and TRs targeting active adhesome genes in the lung are shown in magenta. CHD1, SNAPC4, and SPI1 were classified as inactive, as described in the main Results. (B) Network of interactions between all active lung fibroblast TRs targeting active adhesome genes. TRs identified as significant in main Fig 3B–3C are represented as red nodes, and the remaining TRs are represented as blue nodes. Red self-loops indicate that a TR targets itself.
(PDF)

S19 FigSpatial proximity analysis of transcription regulator targets.(A) Histograms of the number of TRs targeting active adhesome genes and the number of TRs targeting a set of random active non-adhesome genes (Wilcoxon Rank-Sum *p*-value $<2\times 10^{-10}$). (B) Histograms of interchromosomal Hi-C contacts among active adhesome TR targets and random active non-adhesome non-TR target genes located on the same chromosomes (Wilcoxon Rank-Sum test, *p*-value $<3\times 10^{-308}$). (C) Histograms of interchromosomal Hi-C contacts among active adhesome TR targets and among random active non-adhesome non-TR target genes located on the same chromosomes, separately for each TR. The plots are organized in ascending order of Wilcoxon Rank-Sum *p*-value.(PDF)

S20 Fig The identified active human lung fibroblast TRs target fewer adhesome genes in other cell lines and are quite specific to human lung fibroblasts. (A) Number of unique TRs present in the hTFtarget data set for different adherent and non-adherent cell lines. (B) Number of unique TR-target links present in the hTFtarget data set for different cell lines. The cell lines HepG2, GM12878, HEK293, A549 and normal lung fibroblasts (combination of IMR-90, WI38, AG04450, HPF and NHLF) have the highest amount of data. (C) Table showing the proportion of adhesome genes targeted by selected lung fibroblast TRs in different cell lines, including lung fibroblasts, HepG2, GM12878, HEK293, and A549. Rows shaded in red correspond to the 13 significant TRs identified in lung fibroblasts. (D) Heatmap of the proportion of adhesome genes targeted by TRs in different cell lines compared to the proportion of adhesome genes targeted in normal lung fibroblasts. These include cell lines with adherence properties (A549, HepG2, HEK293) and a non-adherent cell line (GM12878). TRs that target (active and/or inactive) adhesome genes in IMR-90 are in the top 29 rows. The remaining TRs do not target adhesome genes in IMR-90 but target at least one adhesome gene in other cell lines.
(PDF)

S21 Fig Projection of LAS submatrices onto adhesome loci. (A) Heatmap of transformed Hi-C contact frequencies among active adhesome and adhesome TR loci (frequencies were log-transformed and centerdized using the mean and standard deviation of contact values across the entire genome). Significant submatrices identified by the LAS algorithm and whose average Hi-C value is higher than 2 are shown as black boxes. This heatmap is obtained by combining LAS interacting regions containing adhesome genes from all interchromosomal Hi-C contact matrices (such as, for example, the matrix in main Fig 4A). (B) Bar plot of the number of adhesome genes per adhesome interacting region. Adhesome LAS interacting regions contain at most 3 adhesome genes. (C) Bar plot of the number of adhesome loci per adhesome interacting region. Adhesome LAS interacting regions contain at most 4 adhesome loci. To relate this to (B), note that an adhesome gene may span multiple 250 kb loci.
(PDF)

S22 Fig Statistical significance of the LAS adhesome network. (A) Histogram of the number of LAS nodes containing at least one gene from a collection of 500 random gene sets. Each random gene set is sampled uniformly without replacement among all active non-adhesome genes. The number of nodes containing active adhesome and adhesome TR genes is shown with a red dashed vertical line, corresponding to a *p*-value of 0.008. This shows that a significantly high number of nodes contain adhesome genes under the 1% significance level. (B) Histogram of the number of interactions among LAS nodes containing at least one gene from the collection of 500 random gene sets in (A). The number of interactions among adhesome LAS nodes is shown with a red dashed vertical line, corresponding to a *p*-value of 0.006. This shows that there is a significantly high number of interactions between adhesome LAS nodes at the 1% significance level. (C) Histogram of the proportion of genes belonging to LAS nodes from the collection of 500 random gene sets in (A). The proportion of adhesome genes belonging to adhesome LAS nodes is shown with a red dashed vertical line, corresponding to a *p*-value of 0.002. This shows that there is a significantly high proportion of adhesome genes that belong to adhesome LAS nodes at the 1% significance level. (D) Histogram of average degree computed on networks of LAS nodes containing genes from the collection of 500 random gene sets in (A). The average degree of the adhesome LAS network is shown with a red dashed vertical line, corresponding to a *p*-value of 0.008. This shows that, on average, the degree of adhesome LAS nodes is significantly higher than the degree of LAS nodes containing random genes at the 1% significance level. (E) Histogram of graph density computed on networks of LAS nodes containing genes from the collection of 500 random gene sets in (A). The graph density of the adhesome LAS network is shown with a red dashed vertical line, corresponding to a *p*-value of 0.116. (F) Histogram of the average number of genes per LAS node from the collection of 500 random gene sets in (A); only LAS nodes containing at least one gene from the random gene set at hand are considered. The average number of adhesome genes per adhesome LAS node is shown with a red dashed vertical line, corresponding to a *p*-value of 0.351.
(PDF)

S23 Fig Regulatory correlation of adhesome LAS nodes. (A) Scatterplot and 2D contour plot of pairs of adhesome LAS nodes. The location of a given pair (u, v) on the scatterplot is determined by the average Hi-C contact value (x-axis) and the regulatory correlation (y-axis) associated with the edge u–v of the adhesome LAS network. Pairs that involve at least one TR targeting active adhesome genes are shown in red. The red dashed lines correspond to marginal thresholds on the distribution of average Hi-C contact values (vertical, 0% quantile) and regulatory correlations (horizontal, 85% quantile). (B) Histogram of average transformed Hi-C contact values among all adhesome LAS nodes. The red dashed vertical line corresponds to the minimum value, which is equal to 2 (by design from the LAS thresholding we performed in a previous step, see S7C Fig). (C) Histogram of average Spearman regulatory correlation among all adhesome LAS nodes. The average Spearman regulatory correlation between two adhesome LAS nodes is computed by taking the average of Spearman regulatory correlations between all tuples of adhesome genes in the two nodes, using the 48 regulatory features used throughout this work. The red dashed vertical line corresponds to the 85% quantile, used as a lower regulatory threshold to select interactions among adhesome LAS nodes for the adhesome LAS network of main Fig 4B. (D) Bar plot of the number of unique chromosomes per maximal clique in the adhesome LAS network obtained after dropping interactions whose average Spearman regulatory correlation is below the threshold in (C). Maximal cliques contain at most 5 unique chromosomes, which is consistent with the hypothesis that no more than 5 chromosomes can physically interact at a given location.
(PDF)

S24 Fig Significant Gene Ontology (GO) terms associated to active non-adhesome genes belonging to genomic regions that interact with adhesome interacting regions. Only genes having a strong positive regulatory correlation (upper decile) with the adhesome genes they interact with (in LAS) are considered. A GO term is reported as significant if its FDR-adjusted *p*-value is lower than 10^–2^. Many of the identified GO terms are associated with the ubiquitin-proteasome system.
(PDF)

S25 Fig Gene set enrichment analysis of active non-adhesome genes interacting with active adhesome genes. (A) Histogram of Spearman regulatory correlation between (i) active non-adhesome genes in adhesome interacting regions and (ii) the active adhesome genes with which these genes interact interchromosomally. Regulatory correlation is computed from the 48 regulatory features used throughout this work. The red dashed vertical threshold line marks the upper decile of the distribution. (B) Same as A, with the red dashed vertical threshold line marking the upper percentile of the distribution. (C) Significant Gene Ontology (GO) terms associated to active non-adhesome genes belonging to genomic regions that interact with adhesome interacting regions. Only genes having a strong positive regulatory correlation (upper percentile in B) with the active adhesome genes they interact with (in LAS) are considered. A GO term is reported as significant if its FDR-adjusted *p*-value is lower than 10^–2^.
(PDF)

S26 Fig Left: Heatmap of cell-normalized MERFISH Euclidean distance between active adhesome probe loci. Loci are ordered according to chromosome and genomic position. Cell-normalized Euclidean distances between loci i and j are averaged over all cells containing probes i and j. Cell-normalization corresponds to dividing the physical distance between i and j in cell c by the maximum probe-probe distance in that cell. Right: Heatmap of Euclidean distance between selected random non-adhesome loci. The number of random probe loci per chromosome is chosen equal to that of adhesome probe loci, to allow for a fair comparison. Probe loci were mapped within 500 kb of the corresponding genes (Methods).
(PDF)

S27 FigExtended MERFISH analysis.Distribution of average normalized Euclidean distances between active adhesome gene probes and active non-adhesome gene probes (a MERFISH probe is associated with a gene if it overlaps with the gene). The average interchromosomal Euclidean distance between active adhesome probes is smaller than the average interchromosomal Euclidean distance among active non-adhesome probes (Wilcoxon Rank-Sum *p*-value $<1\times 10^{-18}$).(PDF)

S28 FigBipartite network of active non-adhesome genes interacting with active adhesome genes.(A) Red histogram of the average cell-normalized Euclidean distance between 135 active non-adhesome genes (61 MERFISH probes) and the 17 active adhesome genes (16 MERFISH probes) they interact with. MERFISH probes are associated with a gene if they are less than 100 kb upstream or downstream of the gene. Only gene pairs that interact in LAS and have a strong regulatory correlation (upper decile in S25A Fig) are considered. The red vertical dashed line corresponds to an upper threshold (first quartile) on the distance used to select edges for (C). Blue histogram of the average cell-normalized Euclidean distance between 23 active adhesome MERFISH probes across LAS regions, used for comparison with the red histogram (Wilcoxon Rank Sums test, *p*-value $<2\times 10^{-29}$). (B) Red histogram as in (A). Blue histogram of the average cell-normalized Euclidean distance between 13 active adhesome MERFISH probes (only LAS interactions are reported) (Wilcoxon Rank Sums test, *p*-value = 0.75). (C) Bipartite network of active adhesome genes (10 genes, left) and active non-adhesome genes they interact with (42 genes, right). Only genes with an associated MERFISH probe (less than 100 kb upstream or downstream) are considered; more details on how this network is constructed can be found in the main text. Gene colors indicate the corresponding chromosomes. Edge colors and thicknesses reflect the physical distance between genes. Edges with an average cell-normalized distance above the first quartile threshold of (A) are not drawn.(PDF)

S29 Fig Relationship between Hi-C contact frequency and DNA-MERFISH Euclidean distance between adhesome loci. Heatmap of average cell-normalized DNA-MERFISH Euclidean distance between adhesome LAS nodes. The distance between two nodes is defined by averaging the distances between all DNA-MERFISH probes associated to the first node and all DNA-MERFISH probes associated to the second node. A DNA-MERFISH probe is associated to a given adhesome LAS node if it is located at most 500 kb upstream or downstream of an adhesome gene within that node. Adhesome LAS nodes that do not have an associated DNA-MERFISH probe are represented as grey rows/columns in the heatmap (missing values). Self-distances are also shown in grey. Dark entries (very low distance) typically correspond to pairs of adhesome LAS nodes on the same chromosome.
(PDF)

S30 FigRepresentative examples of (A) max z projected confocal microscopy images labelled with DAPI, pRNA POL2, and DNA-FISH probes to core adhesome genes and negative control genes, and (B) segmented nuclei, adhesome and negative control gene loci masks.(C) Box plots show the median shortest distance between adhesome gene loci and lamina against negative control gene loci (N (biological replicates) = 2, ≥100 nuclei per replicate). (D) Box plot shows the distinct loci of the adhesome gene observed in each nucleus against the negative control (N (biological replicates) = 2, ≥100 nuclei per replicate). (E) Box plot shows the fraction of adhesome genes on each chromosome against negative control genes. (F) The karyogram of adhesome gene loci (green) and negative control gene loci (Red). (G) The radial distribution function represents the density of particles from the nuclear geometric centre. (H) The particle radial distribution function describes how the density of segmented gene loci varies as a function of distance from each locus’s centroid. Here, the PRDF of adhesome genes is represented in green, and the PRDF of negative control genes is represented in red. Scale bars: 20 μm.(PDF)

S31 Fig Schematic representation of the segmentation of adhesome loci, corresponding active transcription sites and evaluation of adhesome loci distribution. (PDF)

SR1 Supplementary Results Extended results for the thresholded adhesome network (S16 Fig), the regulatory network of adhesome transcription regulators (S18B Fig), the functional enrichment of interacting non-adhesome genes (S24 Fig), and adhesion-related genes in the supplementary MERFISH network (S28 Fig). (PDF)

S1 File List of adhesome genes and their activity. Intrinsic and associated adhesome genes colored by activity level (obtained from hierarchical clustering of the combined set of 48 regulatory features as explained in the main text).
(XLSX)

S2 File Interactive version (.html) of the network in S1B Fig. (HTML)

S3 File DNA-FISH probes for core adhesome genes. (XLSX)

S4 File DNA-FISH probes for negative control genes. (XLSX)
