# Supplementary material for: Coupling between adhesome assembly and 3D chromatin organization
Source: PLoS One. 2026 Oct 29;21(10):e0361202. doi: 10.1371/journal.pone.0361202 (PMC13719029; doi:10.1371/journal.pone.0361202)
Supplement: Supplementary file 1 [file pone.0361202.s001.pdf]

(A)

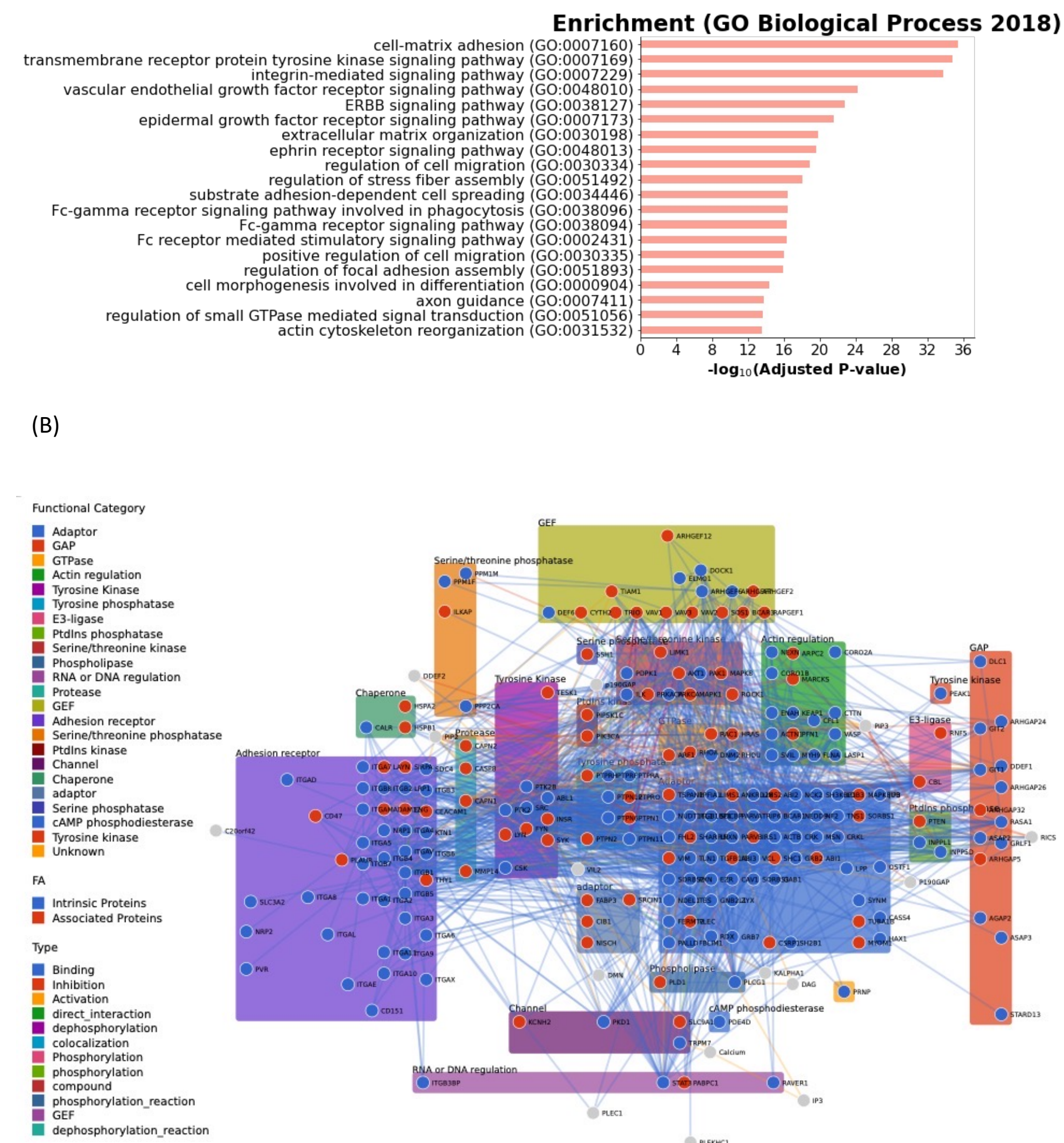

Fig S1: Function and structure of the adhesome. (A) Gene Set Enrichment Analysis of intrinsic and associated adhesome components using the 2018 Gene Ontology database for biological processes. (B) Network of interactions among adhesome proteins (236 nodes, 1501 edges, average degree = 12.7, density = 0.0541). Nodes are colored in blue if they are intrinsic adhesome proteins, and in red if they are associated adhesome proteins. Nodes are grouped by functional categories. Links between nodes are colored based on the type of interaction. An interactive version of the network in .html format is provided as Supplementary File S2.
