# Supplementary material for: Coupling between adhesome assembly and 3D chromatin organization
Source: PLoS One. 2026 Oct 29;21(10):e0361202. doi: 10.1371/journal.pone.0361202 (PMC13719029; doi:10.1371/journal.pone.0361202)
Supplement: Supplementary file 2 [file pone.0361202.s002.pdf]

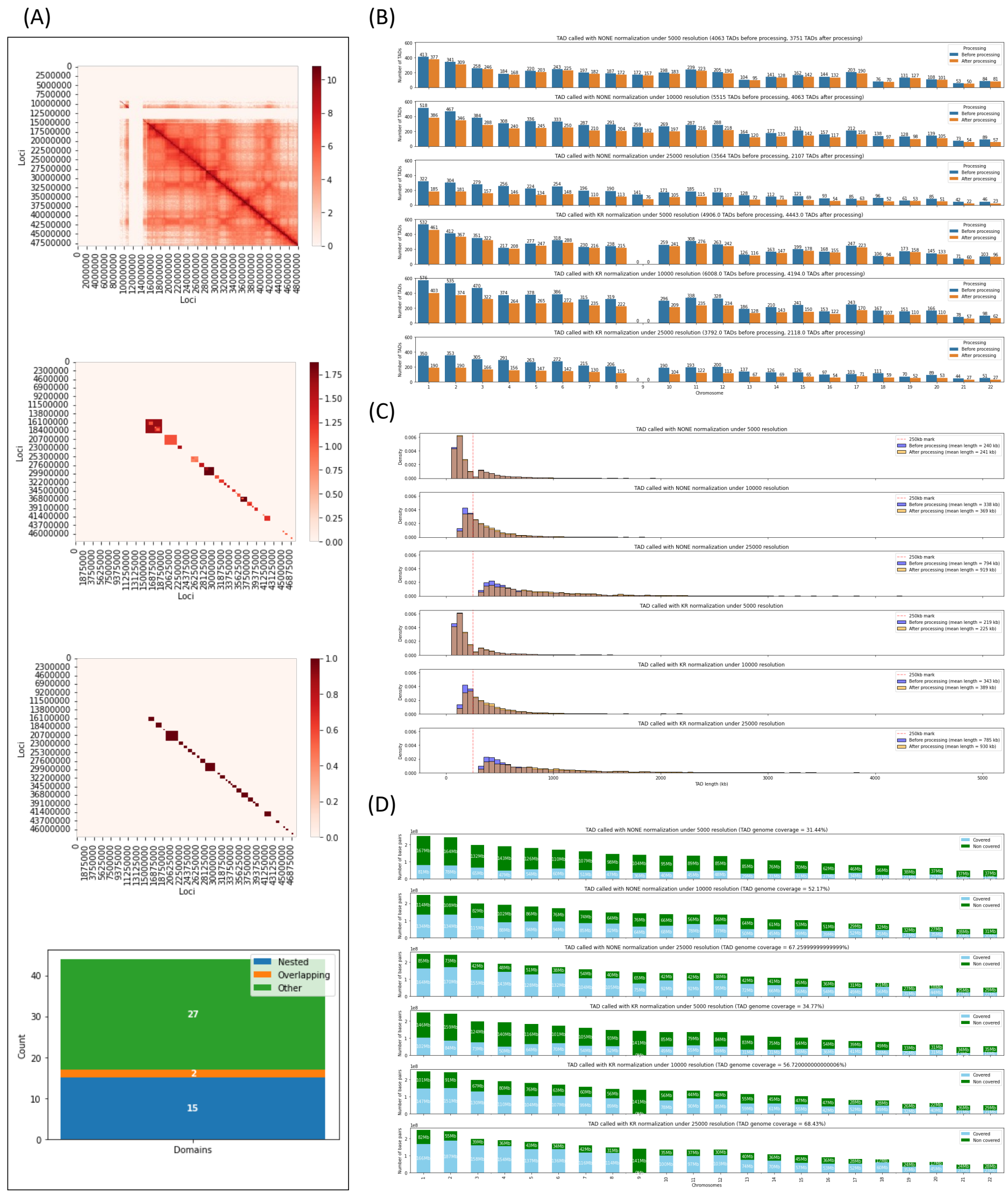

Fig S2: TAD calling. (A) TAD calling pipeline shown for chromosome 21 at resolution 25 kb with Knight-Ruiz normalization. The Arrowhead software (see Methods section) is applied to the normalized intrachromosomal Hi-C contact matrix of chromosome 21 at a given resolution (1st from top), resulting in a list of potentially overlapping and nested TADs (2nd from top). This TAD list is then processed to remove nested TADs and merge overlapping TADs (3rd from top). Most TADs are disjoint, yet a minority of TADs are nested and only very few TADs are overlapping (last from top). (B) Number of TADs called per chromosome before and after post-processing for TAD lists called by Arrowhead at different resolutions (5 kb, 10 kb, 25 kb), with and without Knight-Ruiz normalization. (C) Distribution of TAD length before and after post-processing for TAD lists called by Arrowhead at different resolutions (5 kb, 10 kb, 25 kb), with and without Knight-Ruiz normalization. (D) Number of base pairs covered per chromosome by processed TADs for TAD lists called by Arrowhead at different resolutions (5 kb, 10 kb, 25 kb), with and without Knight-Ruiz normalization.
