# Supplementary material for: Coupling between adhesome assembly and 3D chromatin organization
Source: PLoS One. 2026 Oct 29;21(10):e0361202. doi: 10.1371/journal.pone.0361202 (PMC13719029; doi:10.1371/journal.pone.0361202)
Supplement: Supplementary file 3 [file pone.0361202.s003.pdf]

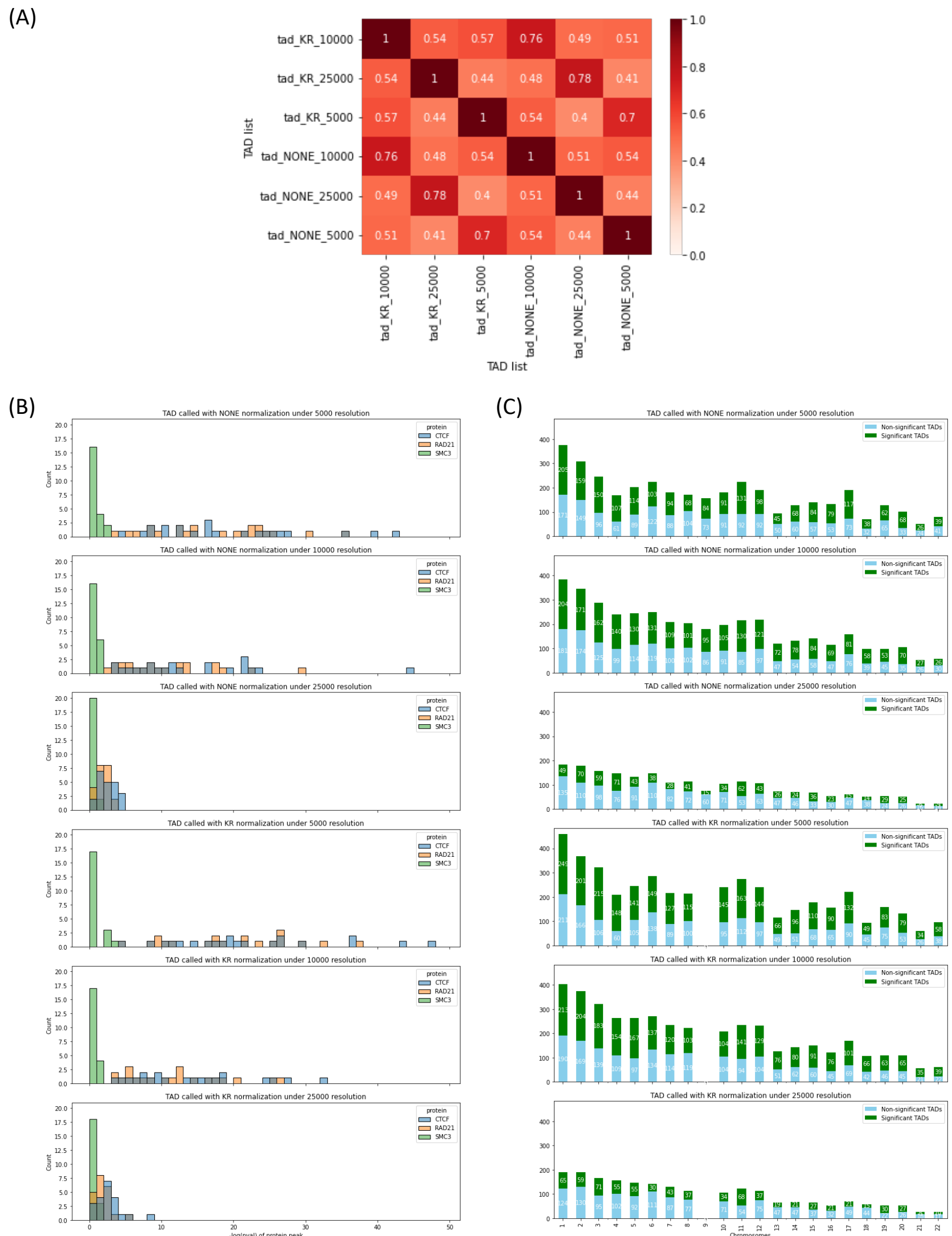

Fig S3: TAD quality. (A) Measure of Concordance (MoC) heatmap for TAD lists called by Arrowhead at different resolutions (5 kb, 10 kb, 25 kb), with and without Knight-Ruiz normalization. An MoC of 1 indicates that 2 TAD lists are identical, while an MoC of 0 indicates that 2 TAD lists are non-overlapping (see Methods). (B) Negative log-p-value of the structural protein profiles (see Methods) of CTCF, RAD21, and SMC3 for TAD lists called by Arrowhead at different resolutions (5 kb, 10 kb, 25 kb), with and without Knight-Ruiz normalization. (C) Number of TADs per chromosome significantly enriched for either transcriptional activating (H3K36me3) or repressing (H3K27me3) histone marks for TAD lists called by Arrowhead at different resolutions (5 kb, 10 kb, 25 kb), with and without Knight-Ruiz normalization. Enrichment is estimated as the log10-ratio between the ChIP-seq signals of H3K36me3 and H3K27me3 and compared with a null distribution derived from random permutations (see Methods).
