# Supplementary material for: Coupling between adhesome assembly and 3D chromatin organization
Source: PLoS One. 2026 Oct 29;21(10):e0361202. doi: 10.1371/journal.pone.0361202 (PMC13719029; doi:10.1371/journal.pone.0361202)
Supplement: Supplementary file 4 [file pone.0361202.s004.pdf]

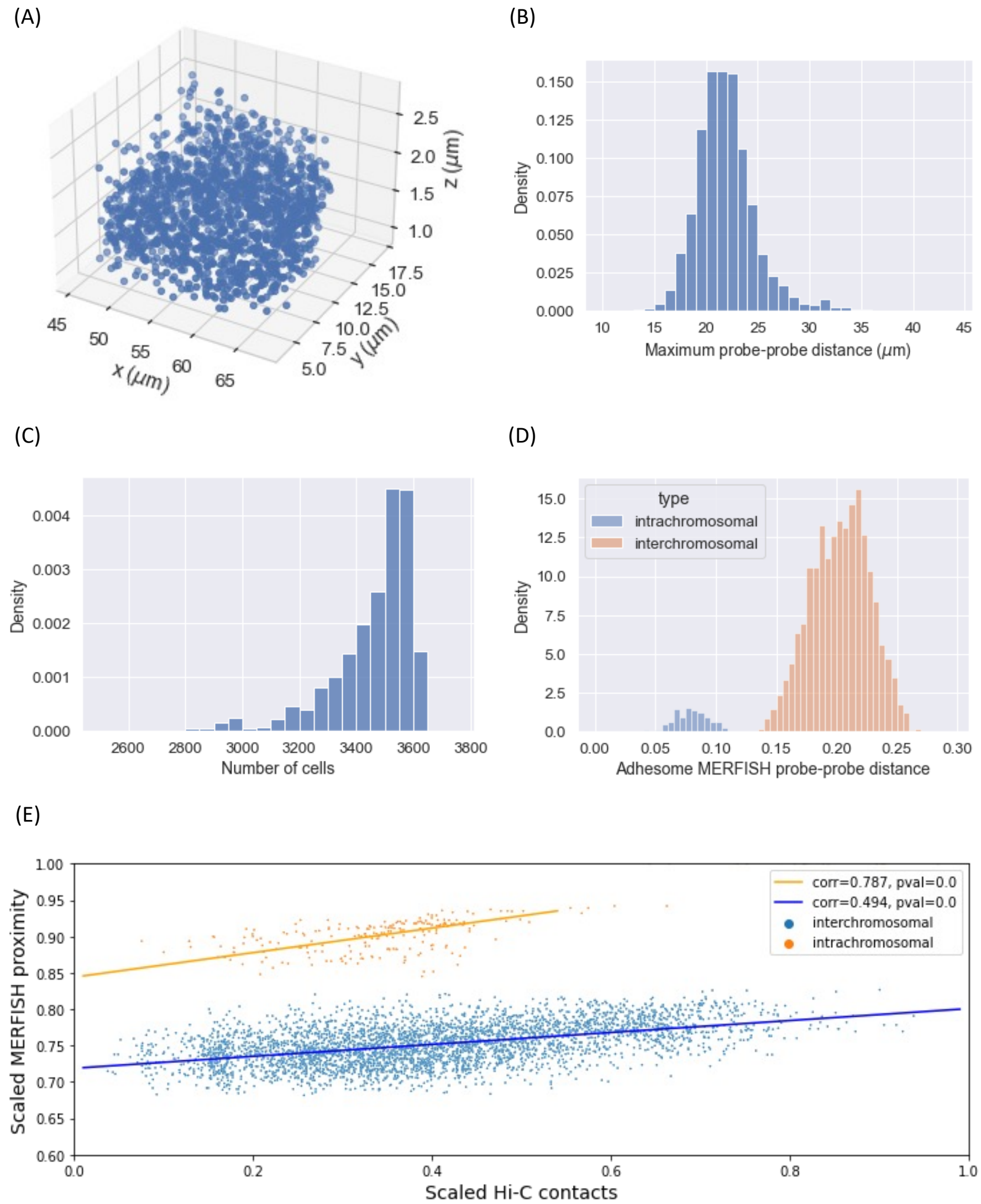

Fig S4: Normalization of Euclidean distances between DNA-MERFISH probes. (A) 3D scatterplot of all 959 DNA-MERFISH probes in the nucleus of a given cell (cell ID 1788). The shape of the nucleus is approximately an ellipsoid with major axis length $\sim$20 μm, which is consistent with the expected nucleus size of IMR-90 cells. (B) Histogram of the maximum distance between pairs of DNA-MERFISH probes for 3,668 cells before downstream filtering. The maximum distance for each cell is used to normalize distances between probes in that cell. (C) Histogram of the number of cells in which a pair of DNA-MERFISH probes is found. The distance metric we use in the main text, i.e., the average cell-normalized Euclidean distance between two probes, corresponds to normalizing the distance between two probes in a cell by the maximum probe-probe distance in the cell in which the two probes are found, then taking the average over all cells that contain these two probes. The histogram shows that this average is taken on at least 2,800 cells and should thus enjoy a high statistical accuracy. (D) Histogram of average cell-normalized Euclidean distances between adhesome DNA-MERFISH probes. Intrachromosomal probe-probe distances are shown in blue, while interchromosomal probe-probe distances are shown in orange. (E) Scatter plot of max-min scaled Hi-C contacts and cell-normalized DNA-MERFISH Euclidean proximity (defined as (1-Euclidean distance)) for interchromosomal adhesome gene pairs (blue, p-value $< 3 \times 10^{-308}$, see ref. [1]) and intrachromosomal adhesome gene pairs (orange, p-value $< 3 \times 10^{-308}$).
