# Supplementary material for: Coupling between adhesome assembly and 3D chromatin organization
Source: PLoS One. 2026 Oct 29;21(10):e0361202. doi: 10.1371/journal.pone.0361202 (PMC13719029; doi:10.1371/journal.pone.0361202)
Supplement: Supplementary file 5 [file pone.0361202.s005.pdf]

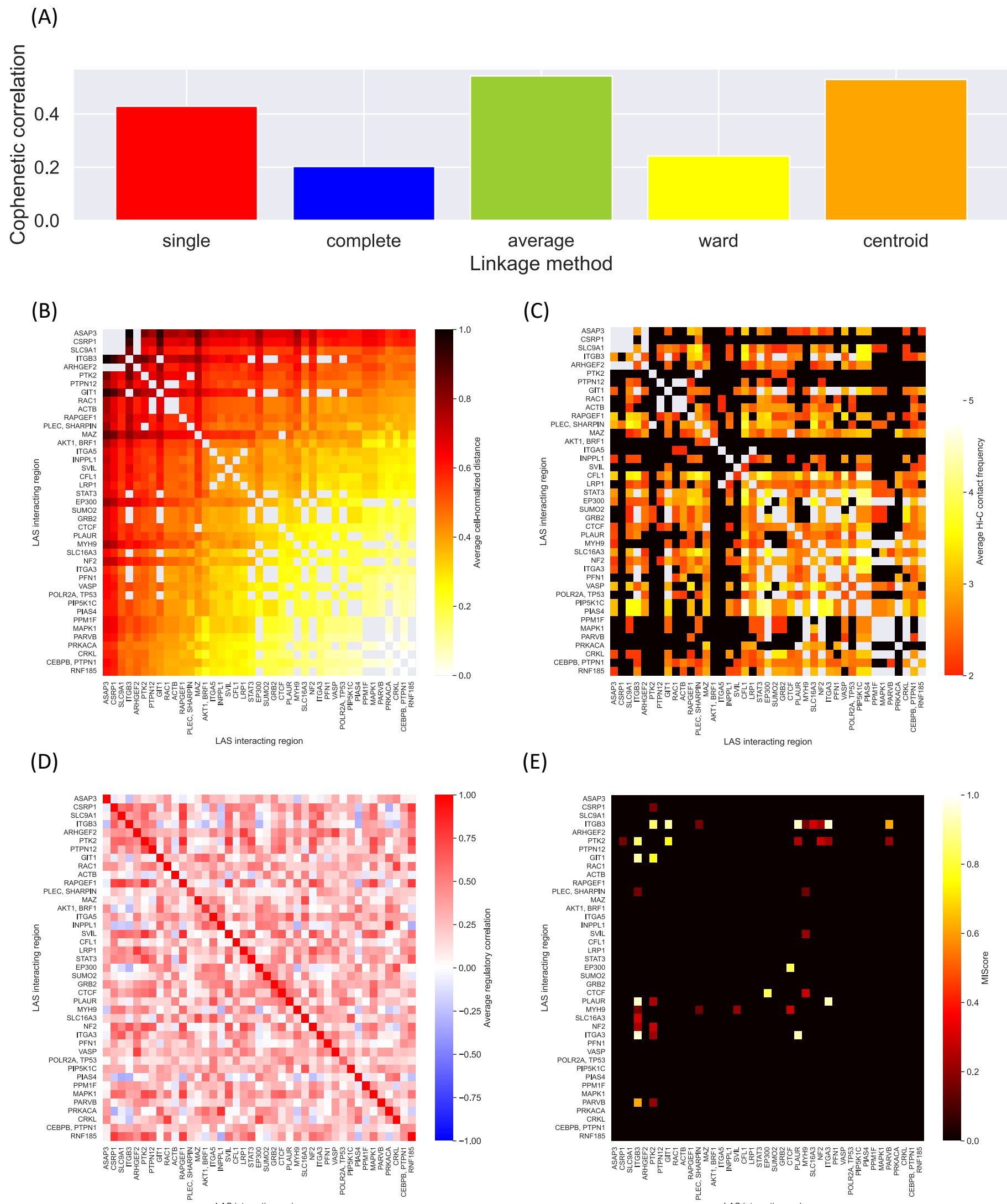


**Fig S5:** Joint analysis of DNA-MERFISH data, Hi-C data, regulatory data, and protein-protein interaction data identifies a cluster of highly co-localized and co-regulated adhesome genes. (A) Bar plot of cophenetic correlation for different linkage functions applied to the average cell-normalized DNA-MERFISH Euclidean distance matrix between adhesome LAS nodes having at least one associated DNA-MERFISH probe. (B) Heatmap of average cell-normalized DNA-MERFISH Euclidean distance between adhesome LAS nodes having at least one associated DNA-MERFISH probe. Rows and columns are ordered using hierarchical clustering with single linkage. Single linkage produces a dendrogram that is reasonably faithful to the original Euclidean distances (cophenetic correlation 0.4, see (A)) and yields an interpretable ordering of the heatmap. Entries of the heatmap that correspond to self-interactions and intrachromosomal interactions are shown in grey. (C) Heatmap of average transformed Hi-C contact frequency between adhesome LAS nodes having at least one associated DNA-MERFISH probe. Rows and columns are ordered as in (B). Entries of the heatmap that correspond to self-interactions and intrachromosomal interactions are shown in grey. Entries corresponding to interactions that were not identified by the LAS algorithm are shown in black. (D) Heatmap of average Spearman regulatory correlation between adhesome LAS nodes having at least one associated DNA-MERFISH probe. Rows and columns are ordered as in (B). (E) Heatmap of protein-protein physical interaction confidence score between genes in adhesome LAS nodes having at least one associated DNA-MERFISH probe. Rows and columns are ordered as in (B). Protein-protein physical interactions are retrieved from the physical STRING network. The confidence score for the interaction of two proteins is based on the number and type of sources reporting this interaction. A score of 0 indicates that two proteins are not known to physically interact.
