# Supplementary material for: Coupling between adhesome assembly and 3D chromatin organization
Source: PLoS One. 2026 Oct 29;21(10):e0361202. doi: 10.1371/journal.pone.0361202 (PMC13719029; doi:10.1371/journal.pone.0361202)
Supplement: Supplementary file 6 [file pone.0361202.s006.pdf]

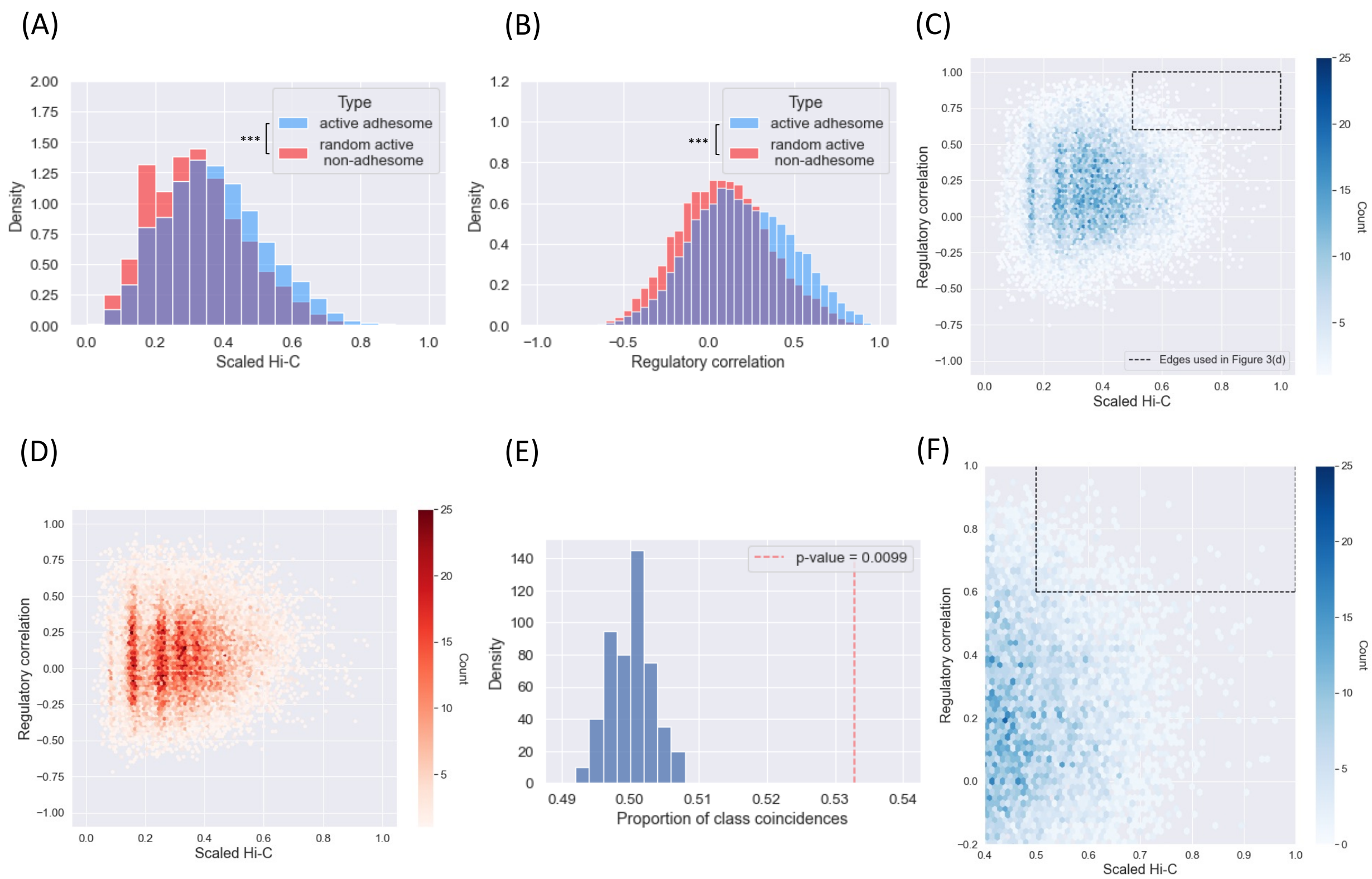

Fig S6: Joint distribution of scaled Hi-C contact values and regulatory correlation among active adhesome genes in IMR-90 cells. (A) Histogram of scaled Hi-C contact values between active adhesome genes (blue) and a random set of active non-adhesome genes (red). There are significantly more contacts between adhesome genes compared to random non-adhesome genes (Wilcoxon Rank-Sums test, p-value = 1e-85). (B) Histogram of regulatory Pearson correlation (based on the 48 regulatory features of Supplementary Fig. S10A) between active adhesome genes (blue) and a random set of active non-adhesome genes (red). There is significantly more coregulation among adhesome genes compared to random non-adhesome genes (Wilcoxon Rank-Sums test, p-value $< 3 \times 10^{-308}$). (C) 2D histogram of scaled Hi-C contact values and Pearson regulatory correlation for active adhesome genes. The dashed box shows the thresholds used to build the network in Supplementary Fig. S16. (D) 2D histogram of scaled Hi-C contact values and Pearson regulatory correlation for random active non-adhesome genes. (E) Simulated null distribution for the permutation test used to assess the difference between the joint distribution of scaled Hi-C contact values and Pearson regulatory correlation for the two gene groups of interest: active adhesome genes (blue, see (C)) and the randomly selected set of active non-adhesome genes (red, see (D)). The test is a two-sample test based on the number of nearest neighbor type co-incidences 3. The red vertical dashed line corresponds to the actual value of the statistic. (F) Zoom into the region around the dashed box of (C).
