# Supplementary material for: Coupling between adhesome assembly and 3D chromatin organization
Source: PLoS One. 2026 Oct 29;21(10):e0361202. doi: 10.1371/journal.pone.0361202 (PMC13719029; doi:10.1371/journal.pone.0361202)
Supplement: Supplementary file 7 [file pone.0361202.s007.pdf]

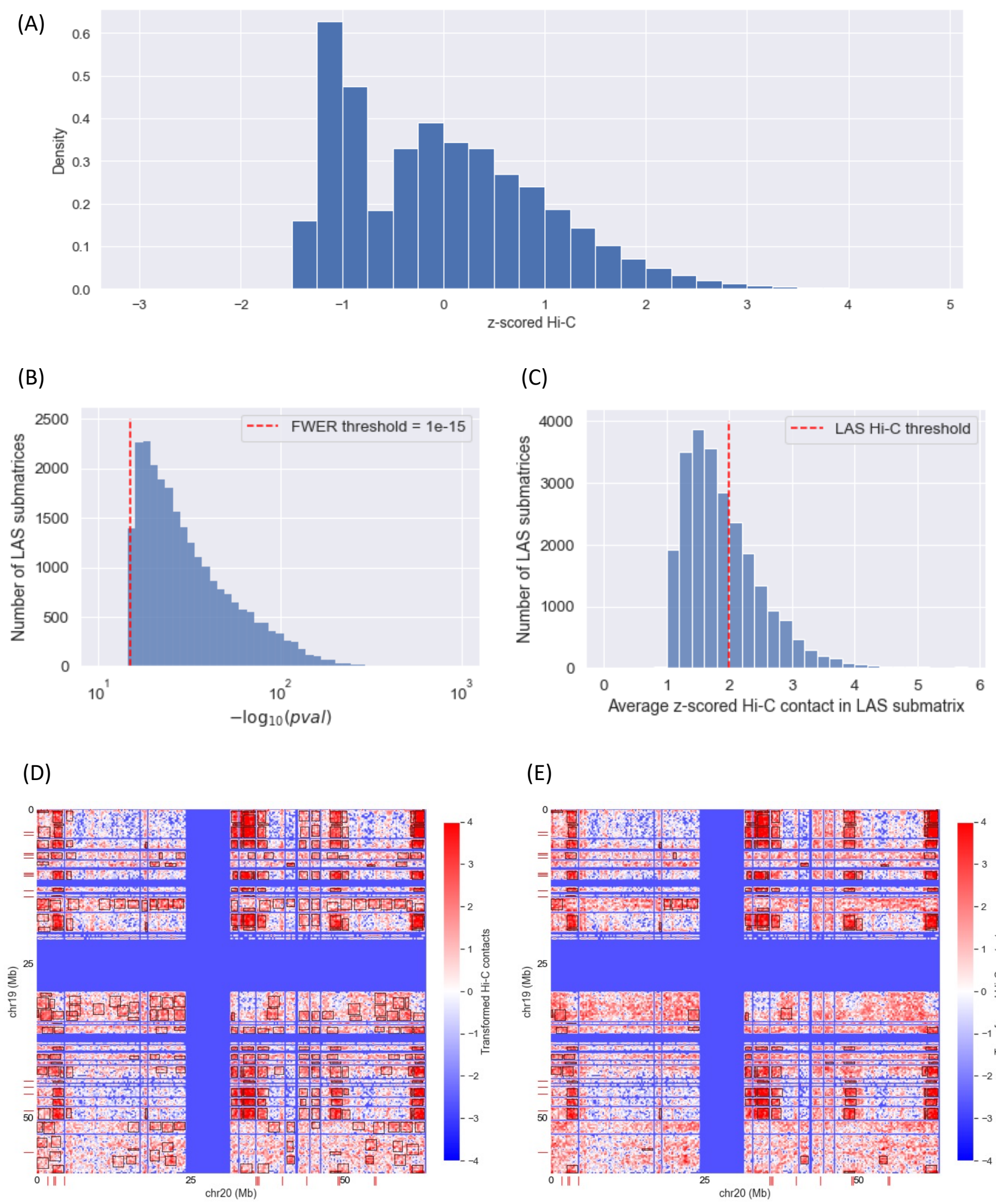

Fig S7: Clustering of transformed Hi-C contact frequencies using LAS algorithm. (A) Histogram of non-zero log-transformed and centerdized Hi-C contact values in the whole genome. Centerdization was performed using the mean and standard deviation of the non-zero contact values across the whole genome. (B) Histogram of negative log10-transformed p-values of submatrices identified by the LAS algorithm [2] in all interchromosomal Hi-C contact matrices. The p-value threshold of 10^{-15} used for signal detection is represented as a red dashed vertical line. This p-value threshold controls the FWER at <0.0001. (C) Histogram of average transformed Hi-C contact values in all LAS submatrices. The Hi-C threshold of 2 used to discard low-average submatrices is represented as a red dashed vertical line. (D) Heatmap of transformed Hi-C contact frequencies between genomic loci on chromosome 19 and chromosome 20. Adhesome loci are indicated by red ticks on the x-axis (for chromosome 20) and the y-axis (for chromosome 19). Significant submatrices identified by the LAS algorithm are shown in black boxes. (E) Same as (D), except that only significant submatrices identified by the LAS algorithm with an average Hi-C contact value higher than 2 are shown in black boxes.
