# Supplementary material for: Coupling between adhesome assembly and 3D chromatin organization
Source: PLoS One. 2026 Oct 29;21(10):e0361202. doi: 10.1371/journal.pone.0361202 (PMC13719029; doi:10.1371/journal.pone.0361202)
Supplement: Supplementary file 8 [file pone.0361202.s008.pdf]

Gene	Functional Category	FA	activity
ACTN1	Actin regulation	Intrinsic Proteins	active
CFL1	Actin regulation	Intrinsic Proteins	active
CORO1B	Actin regulation	Intrinsic Proteins	active
CTTN	Actin regulation	Intrinsic Proteins	active
FLNA	Actin regulation	Intrinsic Proteins	NA
KEAP1	Actin regulation	Intrinsic Proteins	active
LASP1	Actin regulation	Intrinsic Proteins	active
ENAH	Actin regulation	Intrinsic Proteins	active
NEXN	Actin regulation	Intrinsic Proteins	active
SVIL	Actin regulation	Intrinsic Proteins	active
VASP	Actin regulation	Intrinsic Proteins	active
CORO2A	Actin regulation	Intrinsic Proteins	active
ACTB	Adaptor	Intrinsic Proteins	active
JUB	Adaptor	Intrinsic Proteins	NA
SORBS2	Adaptor	Intrinsic Proteins	active
BCAR1	Adaptor	Intrinsic Proteins	active
CAV1	Adaptor	Intrinsic Proteins	active
SMPX	Adaptor	Intrinsic Proteins	NA
SH3KBP1	Adaptor	Intrinsic Proteins	NA
CRK	Adaptor	Intrinsic Proteins	active
CRKL	Adaptor	Intrinsic Proteins	active
EZR	Adaptor	Intrinsic Proteins	active
FHL2	Adaptor	Intrinsic Proteins	active
GAB1	Adaptor	Intrinsic Proteins	active
GRB2	Adaptor	Intrinsic Proteins	active
GRB7	Adaptor	Intrinsic Proteins	active
HAX1	Adaptor	Intrinsic Proteins	active
NEDD9	Adaptor	Intrinsic Proteins	active
CASS4	Adaptor	Intrinsic Proteins	active
TGFB1I1	Adaptor	Intrinsic Proteins	active
ITGB1BP1	Adaptor	Intrinsic Proteins	active
FERMT1	Adaptor	Intrinsic Proteins	inactive
FERMT2	Adaptor	Intrinsic Proteins	active
FERMT3	Adaptor	Intrinsic Proteins	active
LPXN	Adaptor	Intrinsic Proteins	active
PPFIA1	Adaptor	Intrinsic Proteins	active
LPP	Adaptor	Intrinsic Proteins	active
NF2	Adaptor	Intrinsic Proteins	active
FBLIM1	Adaptor	Intrinsic Proteins	active
MSN	Adaptor	Intrinsic Proteins	NA
NCK2	Adaptor	Intrinsic Proteins	active
PALLD	Adaptor	Intrinsic Proteins	active
PARVA	Adaptor	Intrinsic Proteins	active
PARVB	Adaptor	Intrinsic Proteins	active
PXN	Adaptor	Intrinsic Proteins	inactive
LIMS1	Adaptor	Intrinsic Proteins	active
LIMS2	Adaptor	Intrinsic Proteins	active
GNB2L1	Adaptor	Intrinsic Proteins	active
RDX	Adaptor	Intrinsic Proteins	active
OSTF1	Adaptor	Intrinsic Proteins	active
NUDT16L1	Adaptor	Intrinsic Proteins	active
SYNM	Adaptor	Intrinsic Proteins	active
SDCBP	Adaptor	Intrinsic Proteins	active
TLN1	Adaptor	Intrinsic Proteins	active
TNS1	Adaptor	Intrinsic Proteins	active
TES	Adaptor	Intrinsic Proteins	active
TRIP6	Adaptor	Intrinsic Proteins	active
VCL	Adaptor	Intrinsic Proteins	active
SORBS3	Adaptor	Intrinsic Proteins	active
LDB3	Adaptor	Intrinsic Proteins	active
ZYX	Adaptor	Intrinsic Proteins	active
NDEL1	Adaptor	Intrinsic Proteins	active
SH2B1	Adaptor	Intrinsic Proteins	active
TENC1	Adaptor	Intrinsic Proteins	active
ZFYVE21	Adaptor	Intrinsic Proteins	active
SLC3A2	Adhesion receptor	Intrinsic Proteins	active
KTN1	Adhesion receptor	Intrinsic Proteins	active
LRP1	Adhesion receptor	Intrinsic Proteins	active
PVR	Adhesion receptor	Intrinsic Proteins	active
SDC4	Adhesion receptor	Intrinsic Proteins	active
ITGA1	Adhesion receptor	Intrinsic Proteins	active
ITGA2	Adhesion receptor	Intrinsic Proteins	active
ITGA3	Adhesion receptor	Intrinsic Proteins	active
ITGA4	Adhesion receptor	Intrinsic Proteins	active
ITGA5	Adhesion receptor	Intrinsic Proteins	active
ITGA6	Adhesion receptor	Intrinsic Proteins	active
ITGA7	Adhesion receptor	Intrinsic Proteins	inactive
ITGA8	Adhesion receptor	Intrinsic Proteins	active
ITGA9	Adhesion receptor	Intrinsic Proteins	active
ITGA10	Adhesion receptor	Intrinsic Proteins	active
ITGA11	Adhesion receptor	Intrinsic Proteins	active
ITGAD	Adhesion receptor	Intrinsic Proteins	inactive
ITGAE	Adhesion receptor	Intrinsic Proteins	active
ITGAL	Adhesion receptor	Intrinsic Proteins	inactive
ITGAM	Adhesion receptor	Intrinsic Proteins	inactive
ITGAV	Adhesion receptor	Intrinsic Proteins	active
ITGAW	Adhesion receptor	Intrinsic Proteins	NA
ITGAX	Adhesion receptor	Intrinsic Proteins	inactive
ITGB1	Adhesion receptor	Intrinsic Proteins	active
ITGB2	Adhesion receptor	Intrinsic Proteins	active
ITGB3	Adhesion receptor	Intrinsic Proteins	active
ITGB4	Adhesion receptor	Intrinsic Proteins	inactive
ITGB5	Adhesion receptor	Intrinsic Proteins	active
ITGB6	Adhesion receptor	Intrinsic Proteins	active
ITGB7	Adhesion receptor	Intrinsic Proteins	active
ITGB8	Adhesion receptor	Intrinsic Proteins	active
NRP1	Adhesion receptor	Intrinsic Proteins	active
NRP2	Adhesion receptor	Intrinsic Proteins	active
CD151	Adhesion receptor	Intrinsic Proteins	active
PDE4D	cAMP phosphodiesterase	Intrinsic Proteins	active
PKD1	Channel	Intrinsic Proteins	active
TRPM7	Channel	Intrinsic Proteins	active
CALR	Chaperone	Intrinsic Proteins	active
DDEF1	GAP	Intrinsic Proteins	NA
ASAP3	GAP	Intrinsic Proteins	active
GIT1	GAP	Intrinsic Proteins	active
GIT2	GAP	Intrinsic Proteins	active
ARHGAP26	GAP	Intrinsic Proteins	active
GRLF1	GAP	Intrinsic Proteins	NA
ASAP2	GAP	Intrinsic Proteins	active
ARHGAP24	GAP	Intrinsic Proteins	active
DLC1	GAP	Intrinsic Proteins	active
AGAP2	GAP	Intrinsic Proteins	active
STARD13	GAP	Intrinsic Proteins	active
RASA1	GAP	Intrinsic Proteins	active
DEF6	GEF	Intrinsic Proteins	active
DOCK1	GEF	Intrinsic Proteins	active
ELMO1	GEF	Intrinsic Proteins	active
ARHGEF6	GEF	Intrinsic Proteins	NA
ARHGEF7	GEF	Intrinsic Proteins	active
DNM2	GTPase	Intrinsic Proteins	active
RHOU	GTPase	Intrinsic Proteins	active
PLCG1	Phospholipase	Intrinsic Proteins	active
INPP5D	PtdIns phosphatase	Intrinsic Proteins	inactive
INPPL1	PtdIns phosphatase	Intrinsic Proteins	active
ITGB3BP	RNA or DNA regulation	Intrinsic Proteins	active
RAVER1	RNA or DNA regulation	Intrinsic Proteins	active
STAT3	RNA or DNA regulation	Intrinsic Proteins	active
SPTLC1	Serine palmitoyltransferase	Intrinsic Proteins	active
ILK	Serine/threonine kinase	Intrinsic Proteins	active
PAK1	Serine/threonine kinase	Intrinsic Proteins	active
PDPK1	Serine/threonine kinase	Intrinsic Proteins	inactive
PRKCA	Serine/threonine kinase	Intrinsic Proteins	active
PPM1M	Serine/threonine phosphatase	Intrinsic Proteins	active
PPM1F	Serine/threonine phosphatase	Intrinsic Proteins	active
PPP2CA	Serine/threonine phosphatase	Intrinsic Proteins	NA
ABL1	Tyrosine Kinase	Intrinsic Proteins	active
CSK	Tyrosine Kinase	Intrinsic Proteins	active
PTK2	Tyrosine Kinase	Intrinsic Proteins	active
PTK2B	Tyrosine Kinase	Intrinsic Proteins	active
SRC	Tyrosine Kinase	Intrinsic Proteins	active
PEAK1	Tyrosine kinase	Intrinsic Proteins	active
PTPRF	Tyrosine phosphatase	Intrinsic Proteins	active
PTPN12	Tyrosine phosphatase	Intrinsic Proteins	active
PTPRA	Tyrosine phosphatase	Intrinsic Proteins	active
PTPN6	Tyrosine phosphatase	Intrinsic Proteins	active
PTPN11	Tyrosine phosphatase	Intrinsic Proteins	active
PRNP	Unknown	Intrinsic Proteins	active
MYH9	Actin regulation	Intrinsic Proteins	active
MACF1	Actin regulation	Intrinsic Proteins	active
ARPC2	Actin regulation	Associated Proteins	active
MARCKS	Actin regulation	Associated Proteins	active
PFN1	Actin regulation	Associated Proteins	active
ABI1	Adaptor	Associated Proteins	active
ABI2	Adaptor	Associated Proteins	active
ABI3	Adaptor	Associated Proteins	inactive
ANKRD28	Adaptor	Associated Proteins	active
CSRP1	Adaptor	Associated Proteins	active
IRS1	Adaptor	Associated Proteins	active
MAPK8IP3	Adaptor	Associated Proteins	active
PLEC	Adaptor	Associated Proteins	active
SORBS1	Adaptor	Associated Proteins	active
SHC1	Adaptor	Associated Proteins	active
MYOM1	Adaptor	Associated Proteins	inactive
TSPAN1	Adaptor	Associated Proteins	active
TUBA1B	Adaptor	Associated Proteins	active
VIM	Adaptor	Associated Proteins	active
SHARPIN	Adaptor	Associated Proteins	active
FABP3	adaptor	Associated Proteins	active
NISCH	adaptor	Associated Proteins	active
CIB1	adaptor	Associated Proteins	active
CIB2	adaptor	Associated Proteins	inactive
SRCIN1	adaptor	Associated Proteins	inactive
ADAM12	Adhesion receptor	Associated Proteins	active
CEACAM1	Adhesion receptor	Associated Proteins	inactive
ENG	Adhesion receptor	Associated Proteins	inactive
CD47	Adhesion receptor	Associated Proteins	active
LAYN	Adhesion receptor	Associated Proteins	active
SIRPA	Adhesion receptor	Associated Proteins	active
THY1	Adhesion receptor	Associated Proteins	active
PLAUR	Adhesion receptor	Associated Proteins	active
KCNH2	Channel	Associated Proteins	inactive
SLC16A3	Channel	Associated Proteins	active
SLC9A1	Channel	Associated Proteins	active
HSPB1	Chaperone	Associated Proteins	active
HSPA2	Chaperone	Associated Proteins	active
CBL	E3-ligase	Associated Proteins	active
RNF5	E3-ligase	Associated Proteins	active
RNF185	E3-ligase	Associated Proteins	active
ARHGAP5	GAP	Associated Proteins	active
ARHGAP32	GAP	Associated Proteins	active
BCAR3	GEF	Associated Proteins	active
RAPGEF1	GEF	Associated Proteins	active
SOS1	GEF	Associated Proteins	active
TIAM1	GEF	Associated Proteins	active
TRIO	GEF	Associated Proteins	active
VAV1	GEF	Associated Proteins	inactive
VAV2	GEF	Associated Proteins	active
VAV3	GEF	Associated Proteins	inactive
ARHGEF12	GEF	Associated Proteins	active
ARHGEF2	GEF	Associated Proteins	active
CYTH2	GEF	Associated Proteins	active
ARF1	GTPase	Associated Proteins	active
HRAS	GTPase	Associated Proteins	active
RAC1	GTPase	Associated Proteins	active
RHOA	GTPase	Associated Proteins	active
PLD1	Phospholipase	Associated Proteins	active
CAPN1	Protease	Associated Proteins	active
CAPN2	Protease	Associated Proteins	active
CASP8	Protease	Associated Proteins	active
MMP14	Protease	Associated Proteins	active
PIK3CA	PtdIns kinase	Associated Proteins	active
PIP5K1C	PtdIns kinase	Associated Proteins	active
PTEN	PtdIns phosphatase	Associated Proteins	active
PABPC1	RNA or DNA regulation	Associated Proteins	active
SSH1	Serine phosphatase	Associated Proteins	active
AKT1	Serine/threonine kinase	Associated Proteins	active
MAPK1	Serine/threonine kinase	Associated Proteins	active
MAPK8	Serine/threonine kinase	Associated Proteins	active
LIMK1	Serine/threonine kinase	Associated Proteins	active
PRKACA	Serine/threonine kinase	Associated Proteins	active
ROCK1	Serine/threonine kinase	Associated Proteins	active
ILKAP	Serine/threonine phosphatase	Associated Proteins	active
INSR	Tyrosine Kinase	Associated Proteins	active
FYN	Tyrosine Kinase	Associated Proteins	active
LYN	Tyrosine Kinase	Associated Proteins	active
SYK	Tyrosine Kinase	Associated Proteins	inactive
TESK1	Tyrosine Kinase	Associated Proteins	active
PTPN1	Tyrosine phosphatase	Associated Proteins	active
PTPRO	Tyrosine phosphatase	Associated Proteins	active
PTPRH	Tyrosine phosphatase	Associated Proteins	active
PTPN2	Tyrosine phosphatase	Associated Proteins	active

Active gene

Inactive gene

Missing gene

Fig S8: List of adhesome genes and their activity. Intrinsic and associated adhesome genes from ref. [3] colored by activity level (obtained from hierarchical clustering of the combined set of 48 regulatory features as explained in the main text). A searchable version in XLSX format is provided as Supplementary File S1.
