# Supplementary material for: Coupling between adhesome assembly and 3D chromatin organization
Source: PLoS One. 2026 Oct 29;21(10):e0361202. doi: 10.1371/journal.pone.0361202 (PMC13719029; doi:10.1371/journal.pone.0361202)
Supplement: Supplementary file 9 [file pone.0361202.s009.pdf]

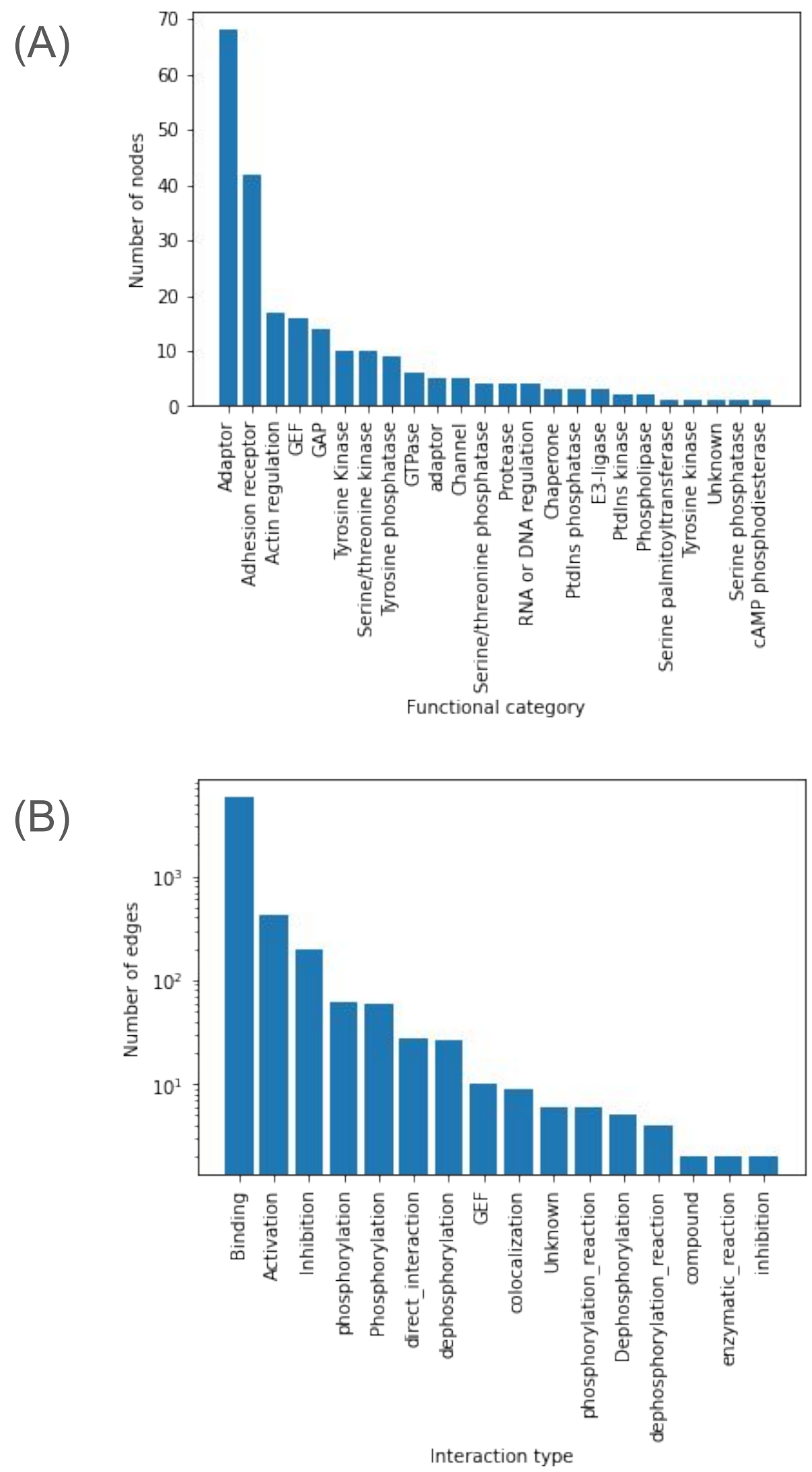

Fig S9: (A) Histogram of the functional categories for all nodes in the network of Supplementary Fig S1B. (B) Histogram of the interaction types for all edges in the network of Supplementary Fig S1B.
