# Supplementary material for: Coupling between adhesome assembly and 3D chromatin organization
Source: PLoS One. 2026 Oct 29;21(10):e0361202. doi: 10.1371/journal.pone.0361202 (PMC13719029; doi:10.1371/journal.pone.0361202)
Supplement: Supplementary file 10 [file pone.0361202.s010.pdf]

(A)

Regulatory mark	Retrieved	Original source	Accession	Category
RNA-seq	ENCODE	ENCODE	ENCSR424FAZ	active
CEBPB	TargetFinder	ENCODE	GSE31477	active
CHD1	TargetFinder	ENCODE	GSE31477	active
CTCF	TargetFinder	ENCODE	GSE31477	active
DNase-seq	TargetFinder	Roadmap Epigenomics	GSE18927	active
EP300	TargetFinder	Jin et al.	GSE43070	active
H2AK5ac	TargetFinder	Roadmap Epigenomics	GSE16256	active
H2AK9ac	TargetFinder	Roadmap Epigenomics	GSE16256	unknown
H2AY	TargetFinder	Chen et al.	GSE54847	unknown
H2AZ	TargetFinder	Roadmap Epigenomics	GSE16256	unknown
H2BK120ac	TargetFinder	Roadmap Epigenomics	GSE16256	unknown
H2BK12ac	TargetFinder	Roadmap Epigenomics	GSE16256	unknown
H2BK15ac	TargetFinder	Roadmap Epigenomics	GSE16256	unknown
H2BK20ac	TargetFinder	Roadmap Epigenomics	GSE16256	unkown
H2BK5ac	TargetFinder	Roadmap Epigenomics	GSE16256	active
H3K14ac	TargetFinder	Roadmap Epigenomics	GSE16256	active
H3K18ac	TargetFinder	Roadmap Epigenomics	GSE16256	active
H3K23ac	TargetFinder	Roadmap Epigenomics	GSE16256	active
H3K27ac	TargetFinder	Roadmap Epigenomics	GSE16256	unkown
H3K27me3	TargetFinder	Roadmap Epigenomics	GSE16256	repressive
H3K36me3	TargetFinder	Roadmap Epigenomics	GSE16256	active
H3K4ac	TargetFinder	Roadmap Epigenomics	GSE16256	unknown
H3K4me1	TargetFinder	Roadmap Epigenomics	GSE16256	active
H3K4me2	TargetFinder	Roadmap Epigenomics	GSE16256	active
H3K4me3	TargetFinder	Roadmap Epigenomics	GSE16256	active
H3K56ac	TargetFinder	Roadmap Epigenomics	GSE16256	unknown
H3K79me1	TargetFinder	Roadmap Epigenomics	GSE16256	unknown
H3K79me2	TargetFinder	Roadmap Epigenomics	GSE16256	unknown
H3K9ac	TargetFinder	Roadmap Epigenomics	GSE16256	active
H3k9me1	TargetFinder	Roadmap Epigenomics	GSE16256	repressive
H3K9me3	TargetFinder	Roadmap Epigenomics	GSE16256	repressive
H4K20me1	TargetFinder	Roadmap Epigenomics	GSE16256	repressive
H4K5ac	TargetFinder	Roadmap Epigenomics	GSE16256	unknown
H4K8ac	TargetFinder	Roadmap Epigenomics	GSE16256	active
H4K91ac	TargetFinder	Roadmap Epigenomics	GSE16256	unknown
LMNB1	TargetFinder	Otte et al.	GSE53332	other
MAFK	TargetFinder	ENCODE	GSE31477	active
MAZ	TargetFinder	ENCODE	GSE31477	active
MECP2	TargetFinder	Maunakea et al.	GSE47678	other
MXI1	TargetFinder	ENCODE	GSE31477	active
POLR2A	TargetFinder	ENCODE	GSE31477	active
RAD21	TargetFinder	ENCODE	GSE31477	other
RB1	TargetFinder	Chicas et al.	GSE19899	repressive
RBL2	TargetFinder	Chicas et al.	GSE19899	repressive
RCOR1	TargetFinder	ENCODE	GSE31477	repressive
RELA	TargetFinder	Jin et al.	GSE43070	active
RFX5	TargetFinder	ENCODE	GSE31477	active
YAP1	TargetFinder	Stein et al.	GSE61852	other

(B)

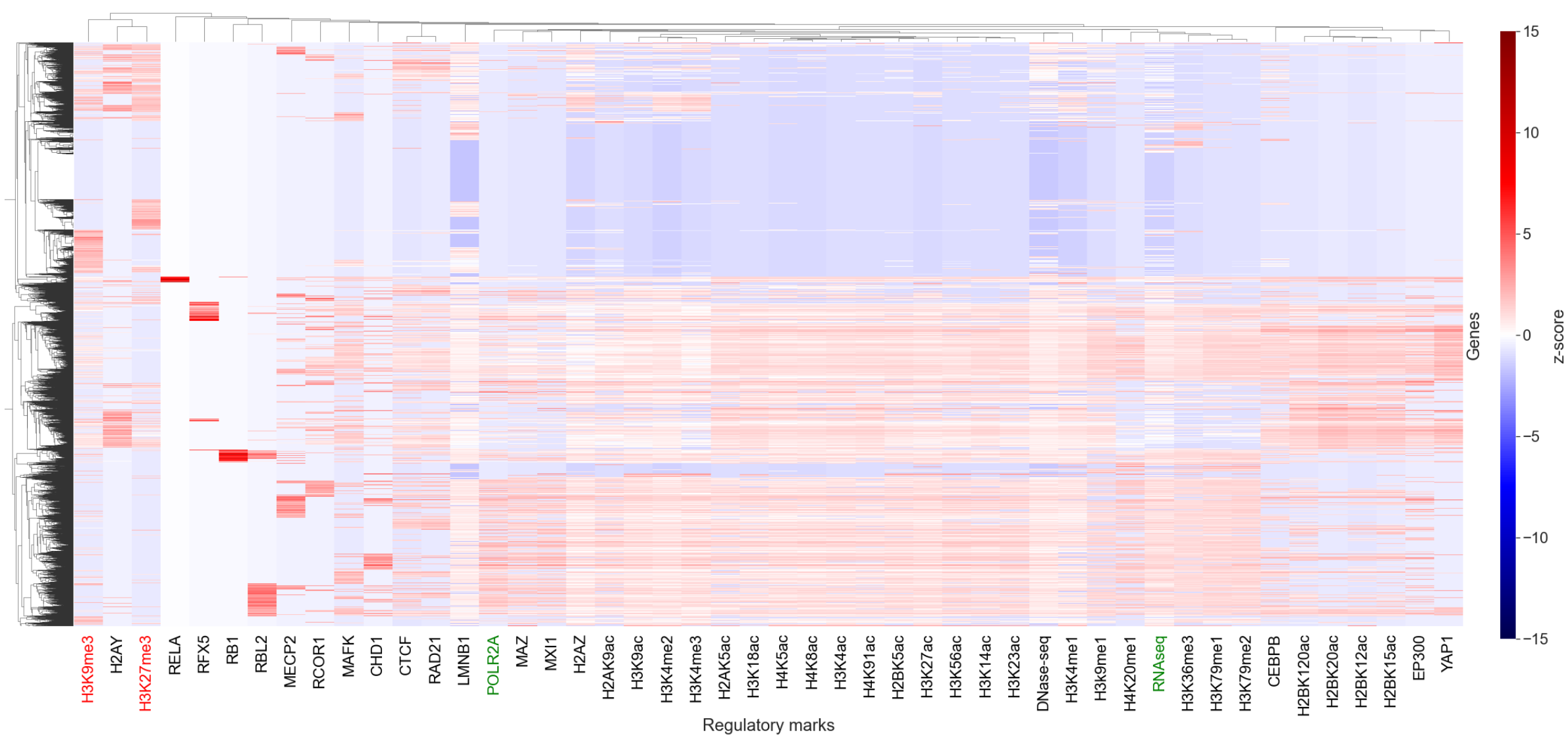

Fig S10: Genome-wide regulatory features in IMR-90 cells. (A) Table describing the 48 regulatory features used in this study, including bulk RNA-seq and 47 cistromic marks (TR and histone ChIP-seq, DNAse-seq). The table also reports the source and accession ID of each data set. Likely annotations on the regulatory activity of each mark are provided when available in the literature (see ref. [4] and Methods). (B) Genome-wide heatmap in IMR-90 cells of the 48 regulatory features in (A). Genes (rows) and regulatory features (columns) were clustered using hierarchical clustering with average linkage and Pearson correlation metric. The feature matrix was normalized and scaled prior to clustering (Methods). The labels of two typical active marks (RNA-seq, POLR2A ChIP-seq) are colored in green and the labels of two typical repressive marks (H3K9me3, H3K27me3 ChIP-seq) are colored in red.
