# Supplementary material for: Coupling between adhesome assembly and 3D chromatin organization
Source: PLoS One. 2026 Oct 29;21(10):e0361202. doi: 10.1371/journal.pone.0361202 (PMC13719029; doi:10.1371/journal.pone.0361202)
Supplement: Supplementary file 11 [file pone.0361202.s011.pdf]

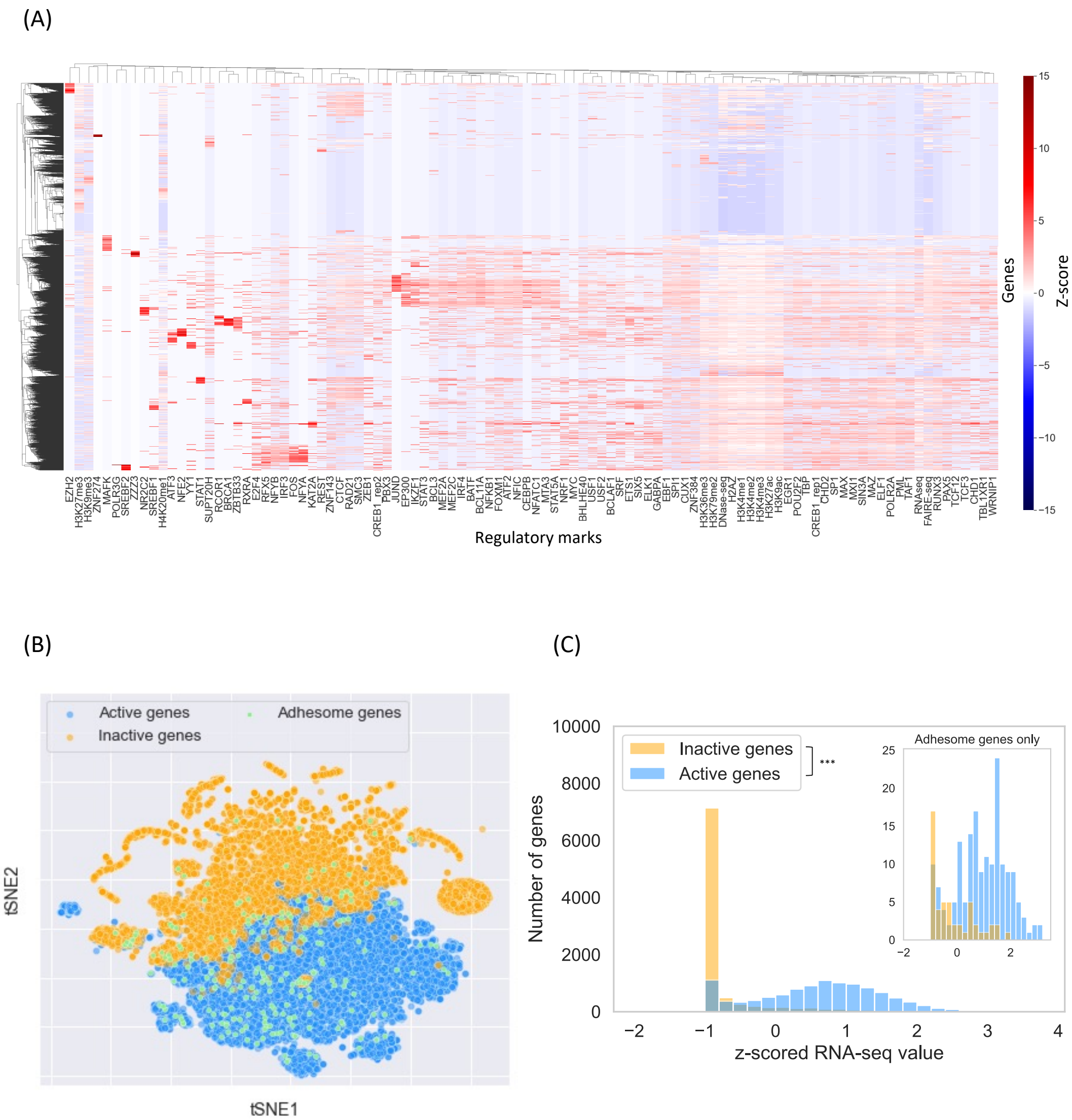

Fig S11: Activity of adhesome genes in GM12878. (A) Heatmap of 100 regulatory marks used to determine the activity of genes in GM12878. Genes (rows) and regulatory marks (columns) were clustered using hierarchical clustering with average linkage and Pearson correlation metric. The feature matrix was normalized and scaled prior to clustering (Methods). (B) tSNE representation of all 25,959 genes based on the 100 GM12878 regulatory marks. Orange dots correspond to inactive genes (9,107 genes, as determined by the clustering in (A)), blue dots correspond to active genes (10,984 genes), and green dots correspond to adhesome genes. Out of the 222 adhesome genes studied (green dots), 172 are active (in contrast to 202 in IMR-90 cells). (C) Histogram of bulk RNA-seq expression in GM12878 for all active genes (blue) and all inactive genes (orange). Active genes have a significantly higher expression than inactive genes (p-value $< 3 \times 10^{-308}$, Wilcoxon rank-sum test). The embedded graph in the upper right corner corresponds to the histogram of RNA-seq expression in GM12878 for all active adhesome genes and all inactive adhesome genes.
