# Supplementary material for: Coupling between adhesome assembly and 3D chromatin organization
Source: PLoS One. 2026 Oct 29;21(10):e0361202. doi: 10.1371/journal.pone.0361202 (PMC13719029; doi:10.1371/journal.pone.0361202)
Supplement: Supplementary file 12 [file pone.0361202.s012.pdf]

(A)

| Pathways | GO/KEGG annotation | Total number of genes | Number of genes active in GM12878 |
|---|---|---|---|
| B cell receptor signaling | GO0050853 | 81 | 63 |
| NF-kB signaling | map04010 | 104 | 66 |
| MAPK signaling | GO0000165 | 218 | 121 |
| Actin cytoskeleton regulation | GO0030036 | 218 | 135 |
| Calcium-mediated signaling | GO0019722 | 240 | 103 |
| PI3-AKT signaling | GO0043491 | 354 | 190 |

(B)

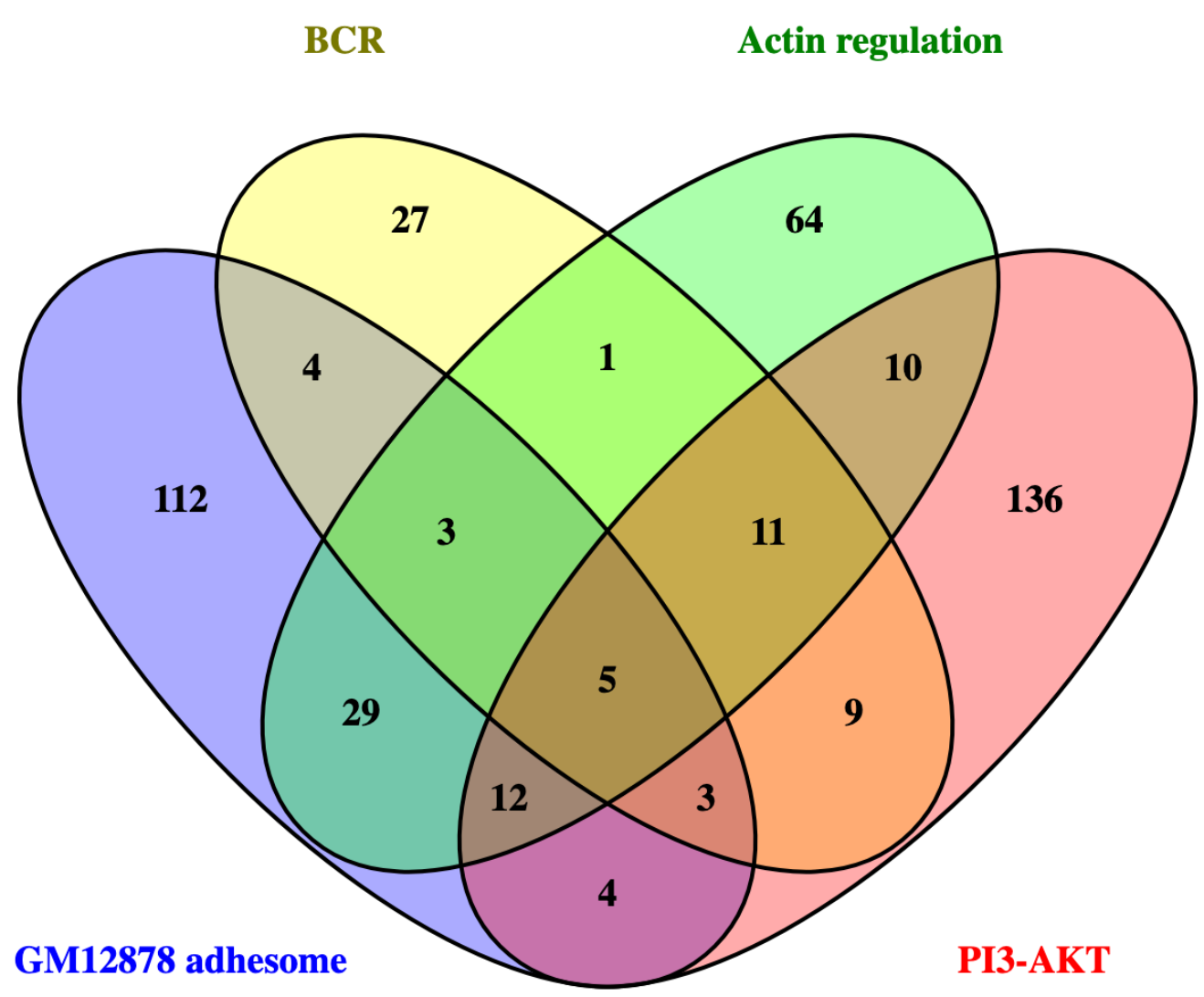


**Fig S12:** Active adhesome genes intersect with B Cell Receptor signaling pathways in GM12878. (A) Table of signaling pathways [5–7] related to the B Cell Receptor (BCR) signaling pathway. We intersect genes in each pathway with the active genes in GM12878 (as determined in Supplementary Fig. S11) to obtain the corresponding number of active pathway genes. (B) Venn diagram of four gene lists: active adhesome genes in GM12878 (blue), active BCR signaling genes (yellow), active actin cytoskeleton regulation genes (green), and active PI3-AKT signaling pathway genes (red). The Venn diagram was drawn using Venny [8].
