# Supplementary material for: Coupling between adhesome assembly and 3D chromatin organization
Source: PLoS One. 2026 Oct 29;21(10):e0361202. doi: 10.1371/journal.pone.0361202 (PMC13719029; doi:10.1371/journal.pone.0361202)
Supplement: Supplementary file 13 [file pone.0361202.s013.pdf]

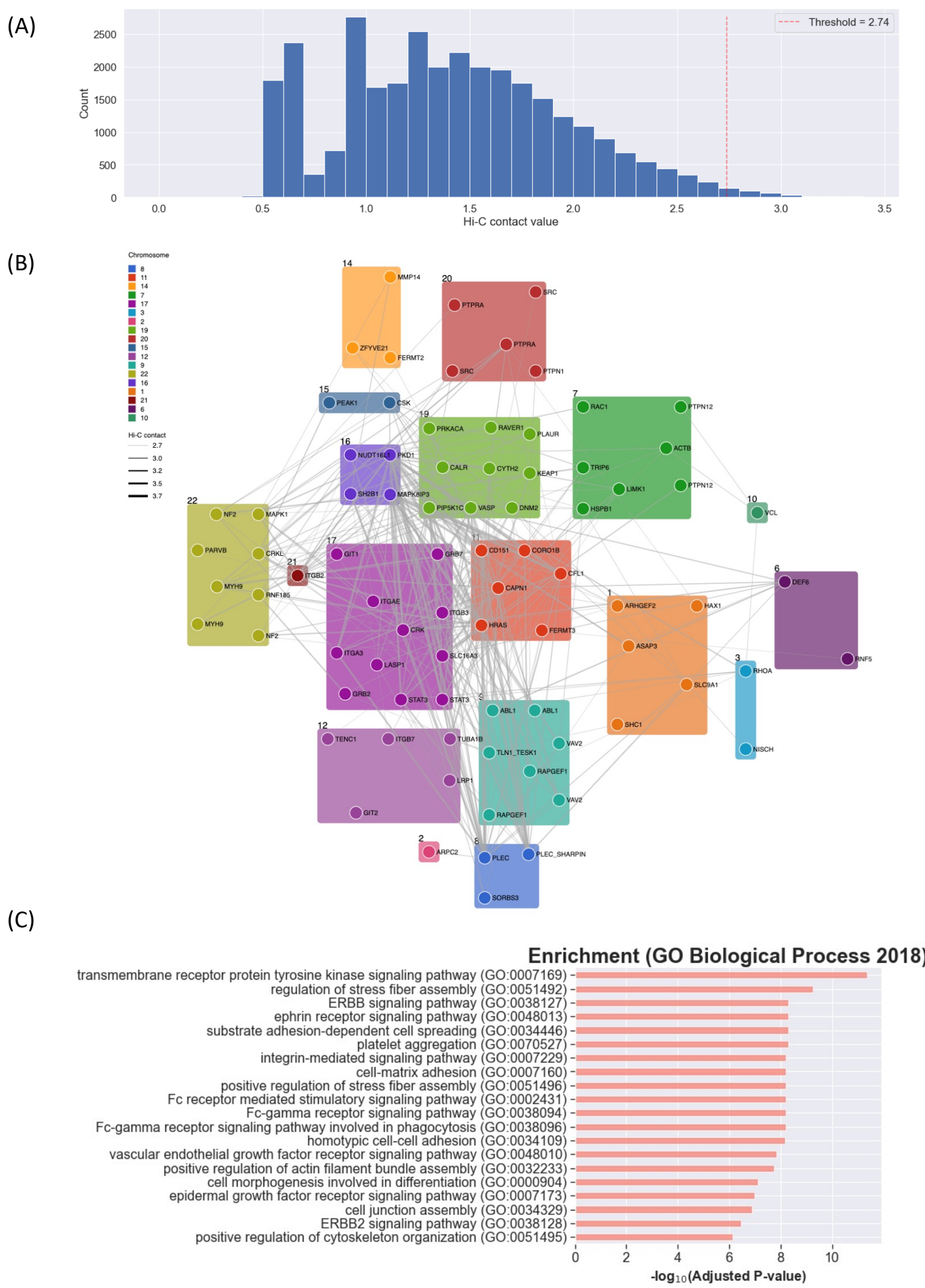

Fig S13: Active adhesome genes with high Hi-C contacts. (A) Distribution of processed interchromosomal Hi-C contact values among active adhesome gene loci. A lower threshold materialized by the dashed red vertical line at 2.74 (99th percentile) is applied to select the strongest loci interactions, which are shown in the network in B. (B) Subnetwork of active adhesome gene loci, colored by chromosomal location and labeled with the active adhesome genes they harbor. Only non-isolated nodes engaging in high Hi-C contacts (Hi-C > 2.74, see A) are shown in the plot; edges are sized according to the corresponding Hi-C contact value. The network contains 82 active adhesome loci (74 adhesome genes) and 321 edges (average degree $= 7.8$). (C) Gene set enrichment analysis of active adhesome genes present in the subnetwork in B using the GO Biological Process 2018 gene sets. Only GO terms with FDR-corrected enrichment p-value smaller than 0.0001 are reported.
