# Supplementary material for: Coupling between adhesome assembly and 3D chromatin organization
Source: PLoS One. 2026 Oct 29;21(10):e0361202. doi: 10.1371/journal.pone.0361202 (PMC13719029; doi:10.1371/journal.pone.0361202)
Supplement: Supplementary file 14 [file pone.0361202.s014.pdf]

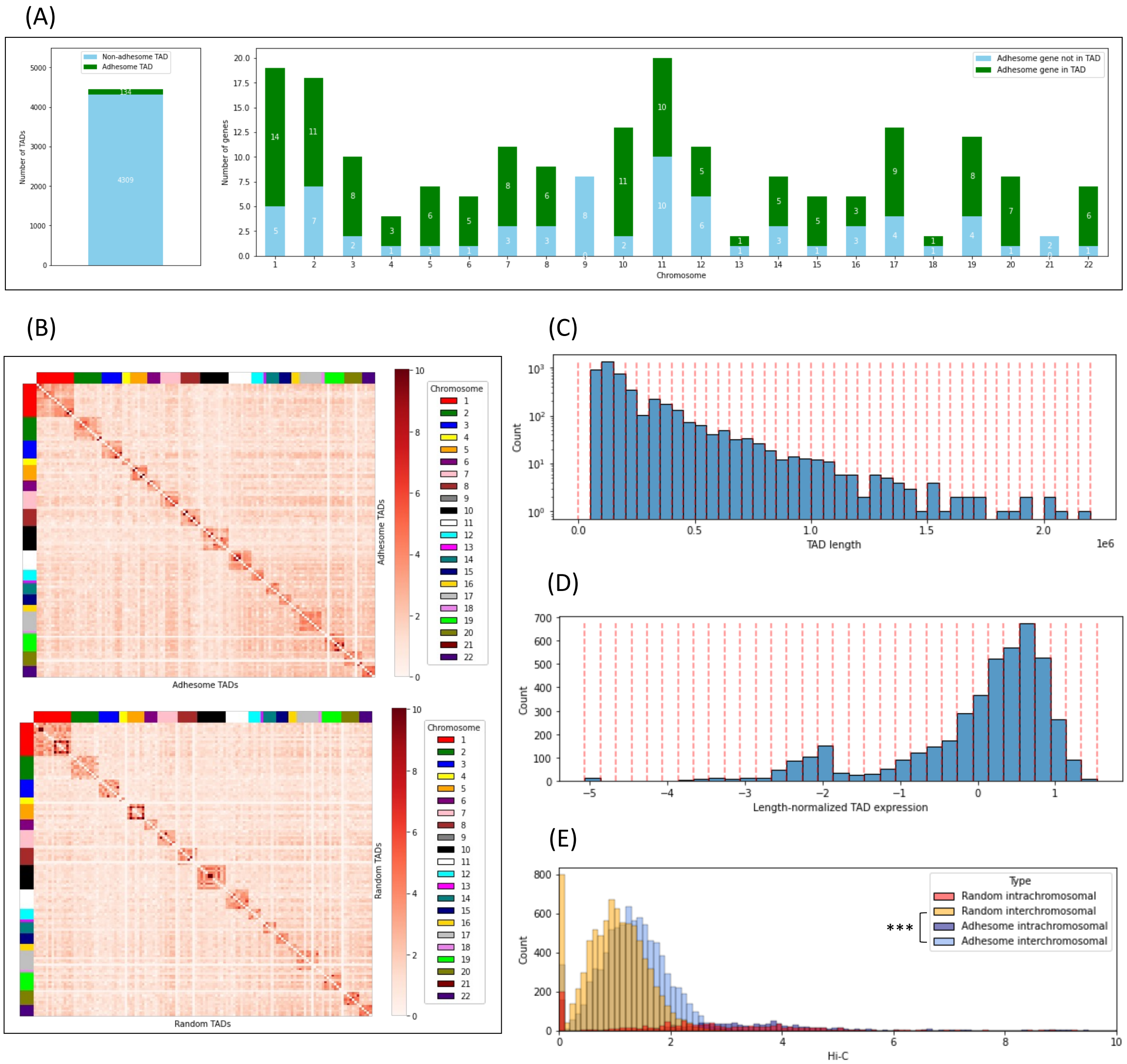

Fig S14: TAD-based interchromosomal proximity analysis. (A) 134 TADs out of 4,443 TADs called by Arrowhead (at resolution 5 kb with Knight-Ruiz normalization) contain at least one active adhesome gene (left). The number of active adhesome genes either in TADs or not in TADs per chromosome is shown in the right sub-panel. (B) Hi-C contact map between active adhesome TADs (top) and random active non-adhesome TADs (bottom). Each row/column corresponds to a TAD. The contact value between two TADs is obtained by averaging the Hi-C contact values associated with these TADs. TADs are grouped by chromosomal location, as shown by the side color bars. The selection of random active adhesome TADs is explained in D and E. (C) Distribution of TAD length for all 4,443 TADs called by Arrowhead at resolution 5 kb with Knight-Ruiz normalization. TADs are stratified into buckets of length 50 kb. These buckets are used to sample background TADs on a per-chromosome basis. (D) Distribution of length-normalized TAD gene expression for all 4,443 TADs called by Arrowhead at resolution 5 kb with Knight-Ruiz normalization. TADs are stratified into buckets of size 0.2. These buckets are used to sample background TADs on a per-chromosome basis. (E) Distribution of interchromosomal and intrachromosomal contacts between active adhesome TADs and between random TADs. Random TADs were selected such that they are similar in size to adhesome TADs, they are similarly distributed over the chromosomes, and they have a similar expression level. The distribution of interchromosomal contacts between active adhesome TADs significantly dominates the distribution of interchromosomal contacts between random TADs (Wilcoxon Rank-Sum test, p-value $< 5 \times 10^{-124}$).
