# Supplementary material for: Coupling between adhesome assembly and 3D chromatin organization
Source: PLoS One. 2026 Oct 29;21(10):e0361202. doi: 10.1371/journal.pone.0361202 (PMC13719029; doi:10.1371/journal.pone.0361202)
Supplement: Supplementary file 15 [file pone.0361202.s015.pdf]

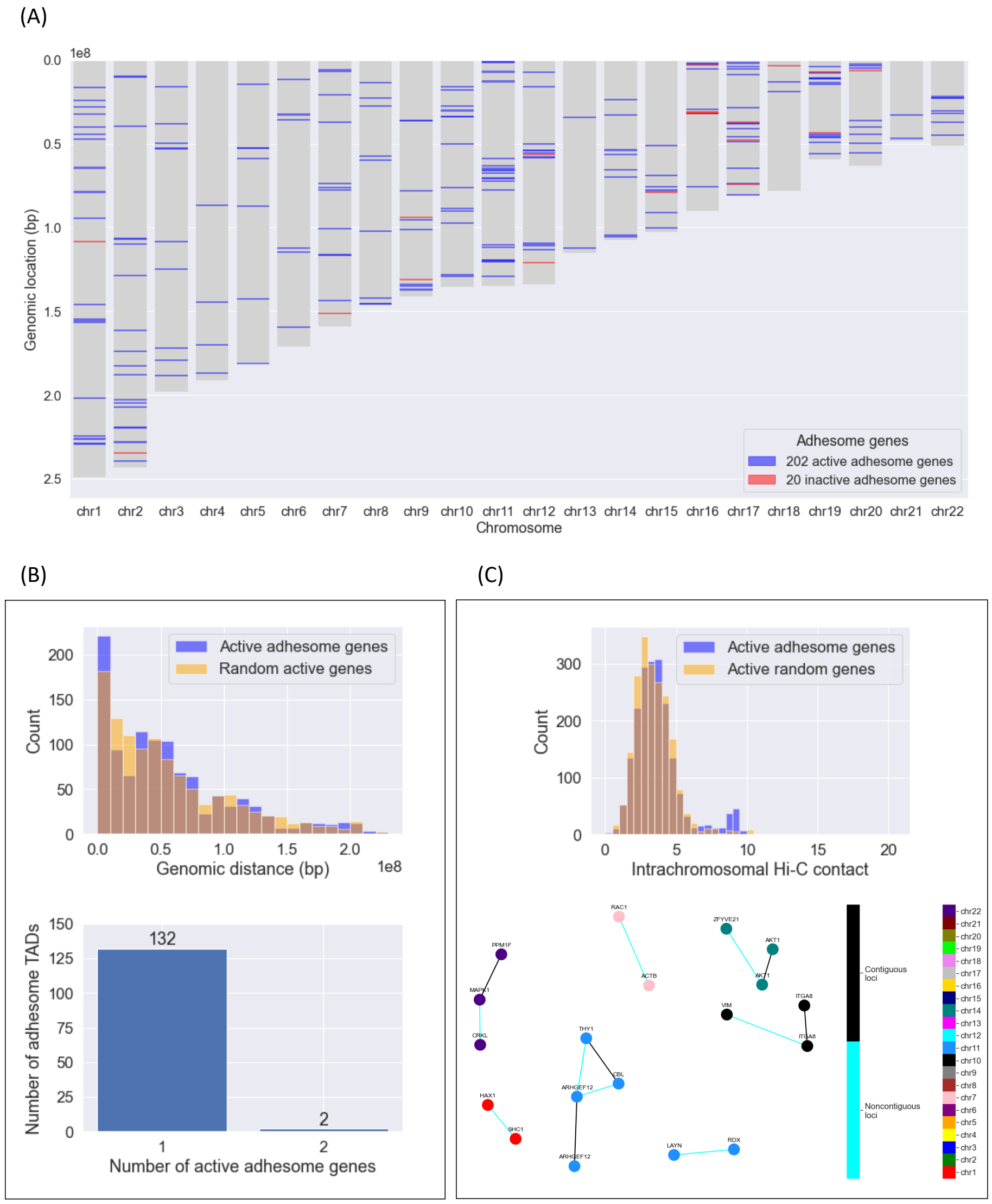

Fig S15: Intrachromosomal proximity analysis. (A) Localization of active and inactive adhesome genes on the chromosomes (using the hg19 reference genome). (B) Top: Distribution of 1D genomic distances (in bp) between active adhesome genes and between a set of random active genes located on the same chromosomes; the distance between 2 genes (located on the same chromosome) is defined as the minimum distance between |start2-end1| and |start1-end2|. Bottom: Number of active adhesome genes per active adhesome TAD, using TADs called by Arrowhead at resolution 5 kb with Knight-Ruiz normalization. (C) Top: Distribution of intrachromosomal Hi-C contacts among active adhesome gene loci and random active non-adhesome loci located on the same chromosomes. Bottom: Subnetwork of active adhesome loci whose intrachromosomal Hi-C contact values are above 7 in the histogram above; only non-degenerate connected components are shown. Adhesome gene loci are colored by their chromosomal location and labeled with the adhesome genes they harbor. Contiguous loci are linked by black edges, while non-contiguous loci are linked by cyan edges.
