# Supplementary material for: Coupling between adhesome assembly and 3D chromatin organization
Source: PLoS One. 2026 Oct 29;21(10):e0361202. doi: 10.1371/journal.pone.0361202 (PMC13719029; doi:10.1371/journal.pone.0361202)
Supplement: Supplementary file 16 [file pone.0361202.s016.pdf]

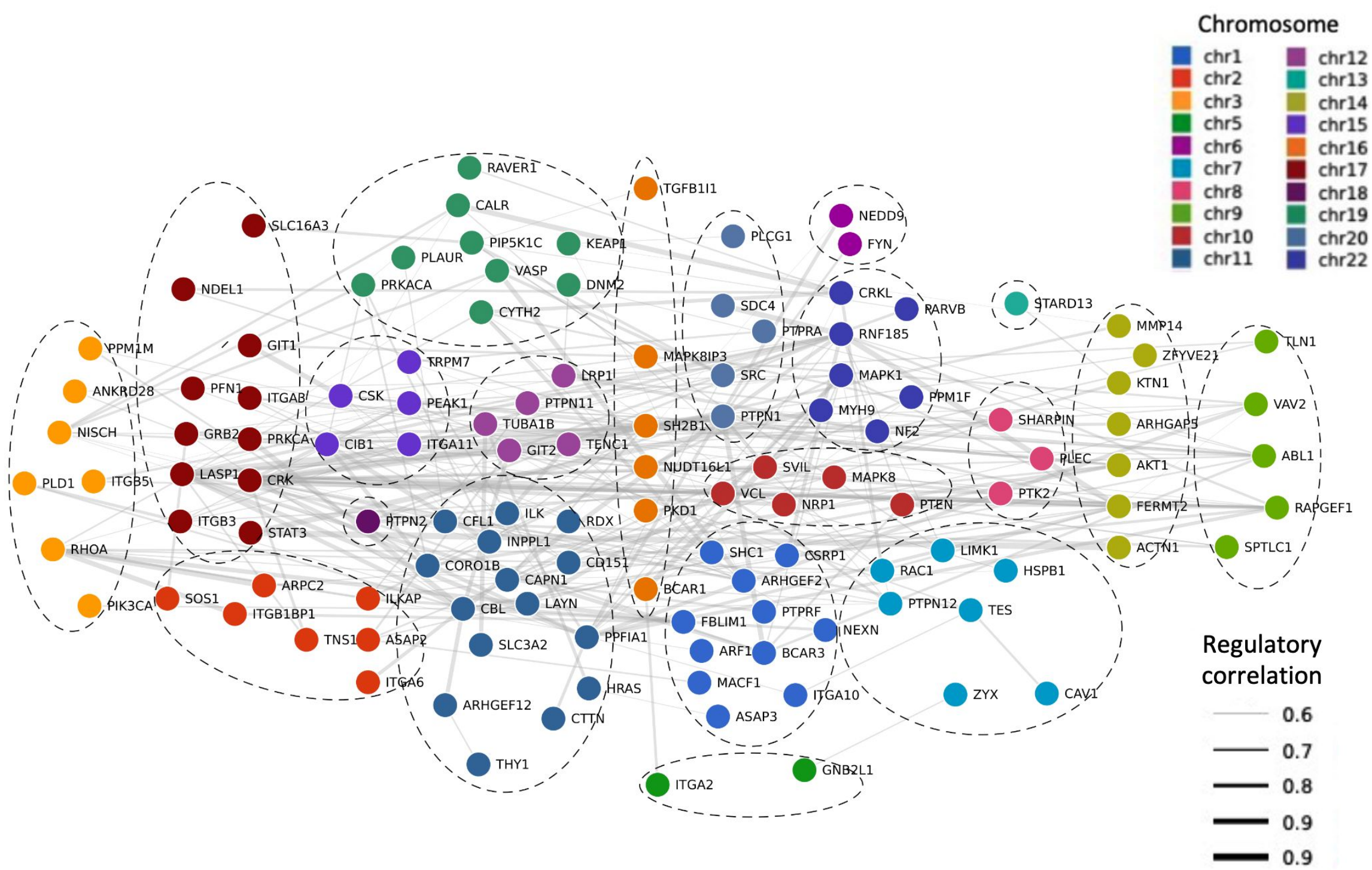

Fig S16: Co-regulatory network of active adhesome genes, where nodes have been grouped by chromosome (dashed ellipses). The network skeleton is obtained by lower-thresholding the pairwise Hi-C contact values (scaled between 0 and 1) at 0.5 and lower-thresholding the pairwise regulatory Pearson correlation at 0.6, which corresponds to the grey-shaded areas of the x- and y-axes in main Fig. 2C. Network weights are proportional to the corresponding pairwise regulatory Pearson correlation. Genes are colored and grouped by chromosome.
