# Supplementary material for: Coupling between adhesome assembly and 3D chromatin organization
Source: PLoS One. 2026 Oct 29;21(10):e0361202. doi: 10.1371/journal.pone.0361202 (PMC13719029; doi:10.1371/journal.pone.0361202)
Supplement: Supplementary file 17 [file pone.0361202.s017.pdf]

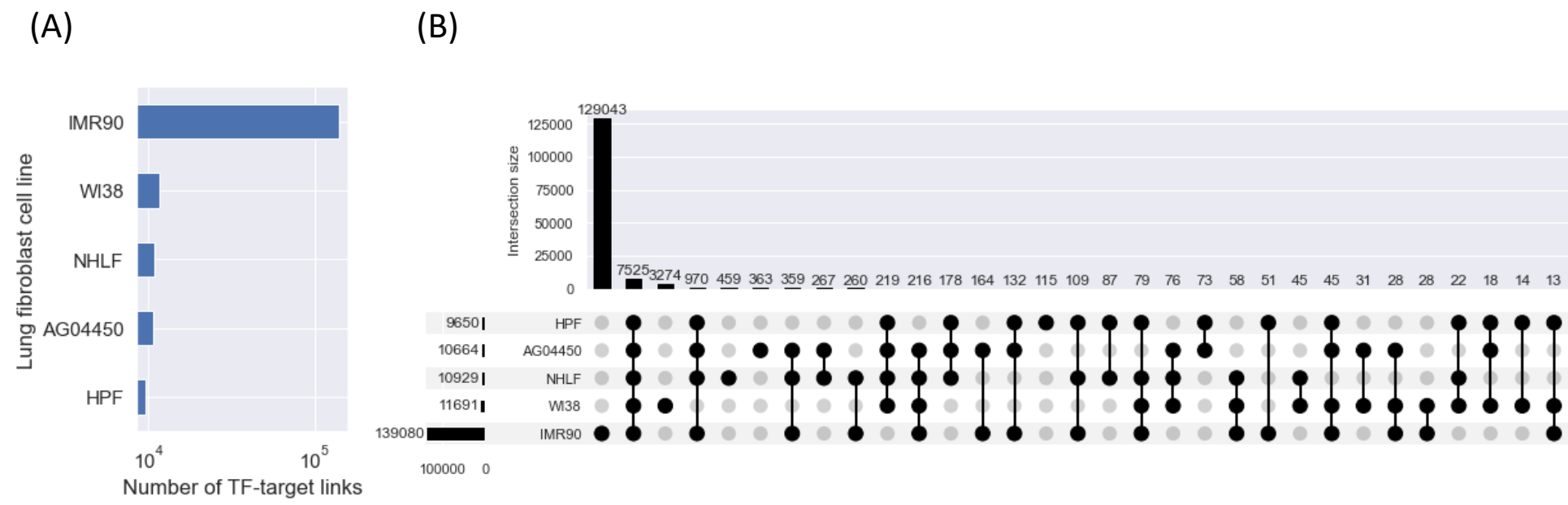

(C)

Cell line	TFs<br>red = inactive ; purple = not present in IMR90 data; brown = target inactive adhesome gene	Number of TFs	Number of active TFs
AG04450	CTCF	1	1
HPF	CTCF	1	1
IMR90	BDP1, BRCA1, BRF1, BRF2, CEBPB, CHD1, CTCF, E2F7, EP300, GTF2B, LMNB1, MAFK, MAZ, MXI1, POLR2A, RAD21, RB1, RBL1, RBL2, RCOR1, RFX5, SNAPC1, SNAPC2, SNAPC4, SNAPC5, SPI1, TP53	27	24
NHLF	CTCF	1	1
WI38	SUMO2, UBE2I, PIAS4, SUMO1, CTCF	5	5

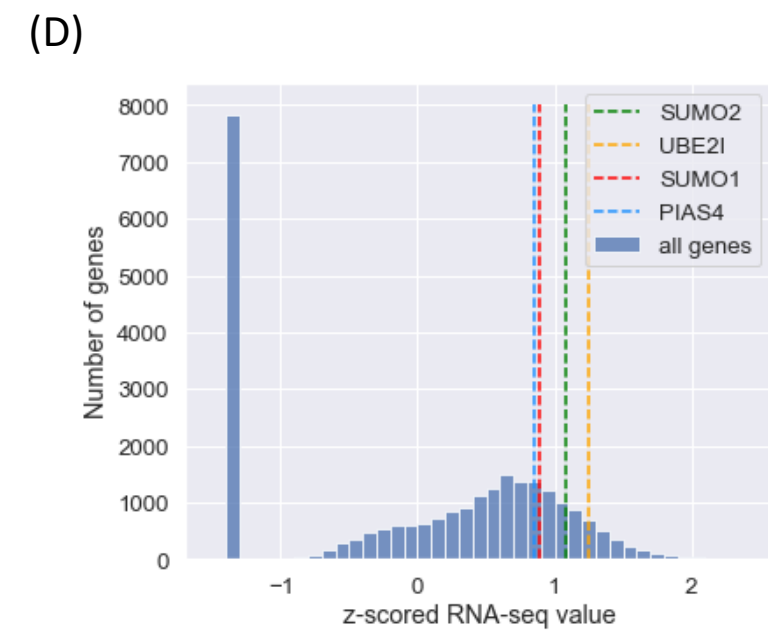

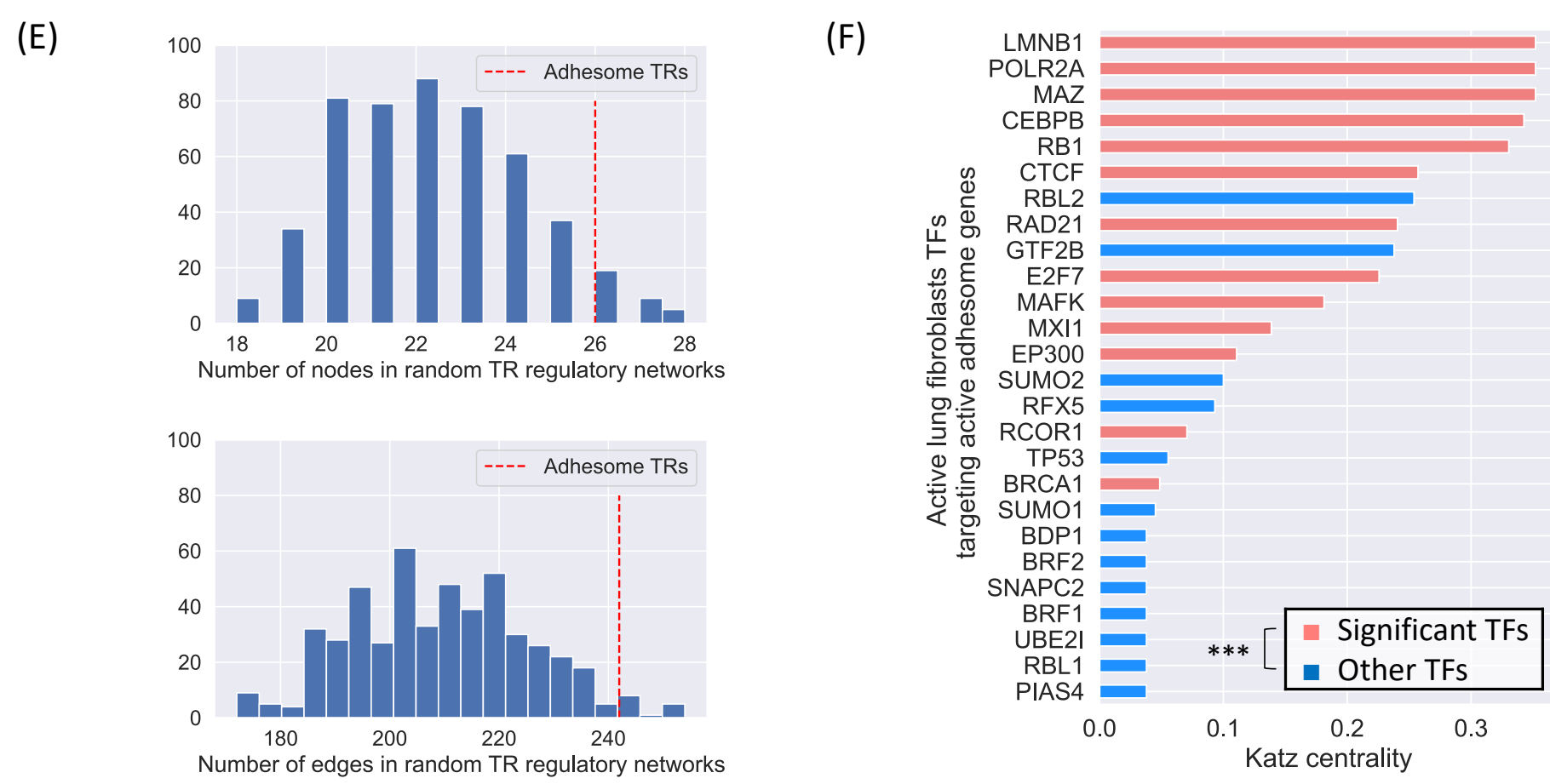

Fig S17: TR-target data in normal human lung fibroblast cell lines. (A) Bar plot of the number of TR-target links from the hTFtarget data set in five human lung fibroblast cell lines (AG04450, HPF, NHLF, WI38, IMR-90). Only targets that are active in IMR-90 are considered. (B) Set overlap plot for the TR-target relationships in cell lines AG04450, HPF, NHLF, WI38, and IMR-90. Only targets that are active in IMR-90 are considered. While most identified TR-target interactions come from the IMR-90 data, other human lung fibroblast cell lines contribute a few additional TR-target interactions that are not present in the IMR-90 data. (C) Table of TRs present for all normal human lung fibroblast cell lines. Red font indicates inactive genes, purple font indicates TRs that are not present in IMR-90, and brown font indicates TRs that target inactive adhesome genes. (D) Histogram of z-scored RNA-seq expression for all genes in IMR-90. The expression of SUMO2, SUMO1, UBE2I and PIAS4 (transcription regulators found in WI38 only) are represented with colored vertical dashed lines. (E) Histograms of the number of nodes (top) and the number of edges (bottom) in 500 random TR regulatory networks (only lung fibroblast TR-target relationships in hTFtarget [9] are considered). Each random network is obtained from the active TRs that target a set of randomly selected genes (with the same cardinality as the set of active adhesome genes). The red dashed vertical lines report values of nodes (p-value $= 0.066$) and edges (p-value $= 0.028$) corresponding to the network of active adhesome TRs. (F) Bar plot of Katz centrality of all active lung fibroblast TRs in the network of Supplementary Fig. S18B. The parameter alpha of Katz centrality was chosen to be 1/s-0.01, where s is the largest eigenvalue (in magnitude) of the adjacency matrix of the network in Supplementary Fig. S18B. Bars are colored in red if they correspond to significant TRs from main Fig. 3C, otherwise they are colored in blue. There is a significant enrichment of significant TRs at the top of the list (XL-minimal hypergeometric test [10], p-value $< 6 \times 10^{-4}$ and optimal cutoff value at 17, which corresponds to BRCA1). The parameters X and L were chosen to be 10% of the number of significant TRs and 150% of the number of significant TRs, respectively.
