# Supplementary material for: Coupling between adhesome assembly and 3D chromatin organization
Source: PLoS One. 2026 Oct 29;21(10):e0361202. doi: 10.1371/journal.pone.0361202 (PMC13719029; doi:10.1371/journal.pone.0361202)
Supplement: Supplementary file 18 [file pone.0361202.s018.pdf]

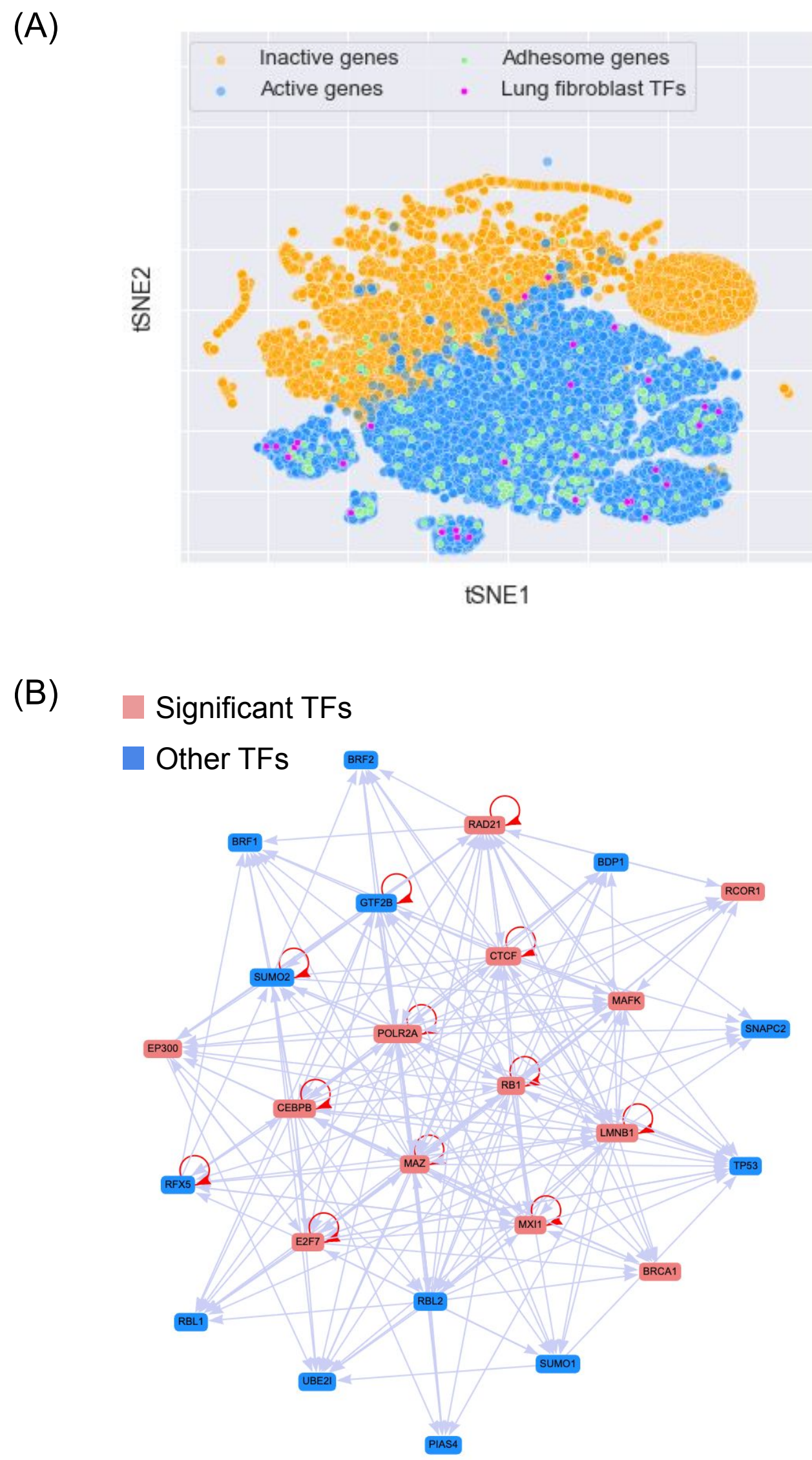

Fig S18: (A) tSNE representation of all 25,959 genes based on 48 IMR-90 regulatory features. Orange dots correspond to inactive genes (as determined by the corresponding expression of regulatory marks), and blue dots correspond to active genes. Adhesome genes are shown in green, and TRs targeting active adhesome genes in the lung are shown in magenta. CHD1, SNAPC4, and SPI1 were classified as inactive, as described in the main Results. (B) Network of interactions between all active lung fibroblast TRs targeting active adhesome genes. TRs identified as significant in main Fig. 3B–C are represented as red nodes, and the remaining TRs are represented as blue nodes. Red self-loops indicate that a TR targets itself.
