# Supplementary material for: Coupling between adhesome assembly and 3D chromatin organization
Source: PLoS One. 2026 Oct 29;21(10):e0361202. doi: 10.1371/journal.pone.0361202 (PMC13719029; doi:10.1371/journal.pone.0361202)
Supplement: Supplementary file 19 [file pone.0361202.s019.pdf]

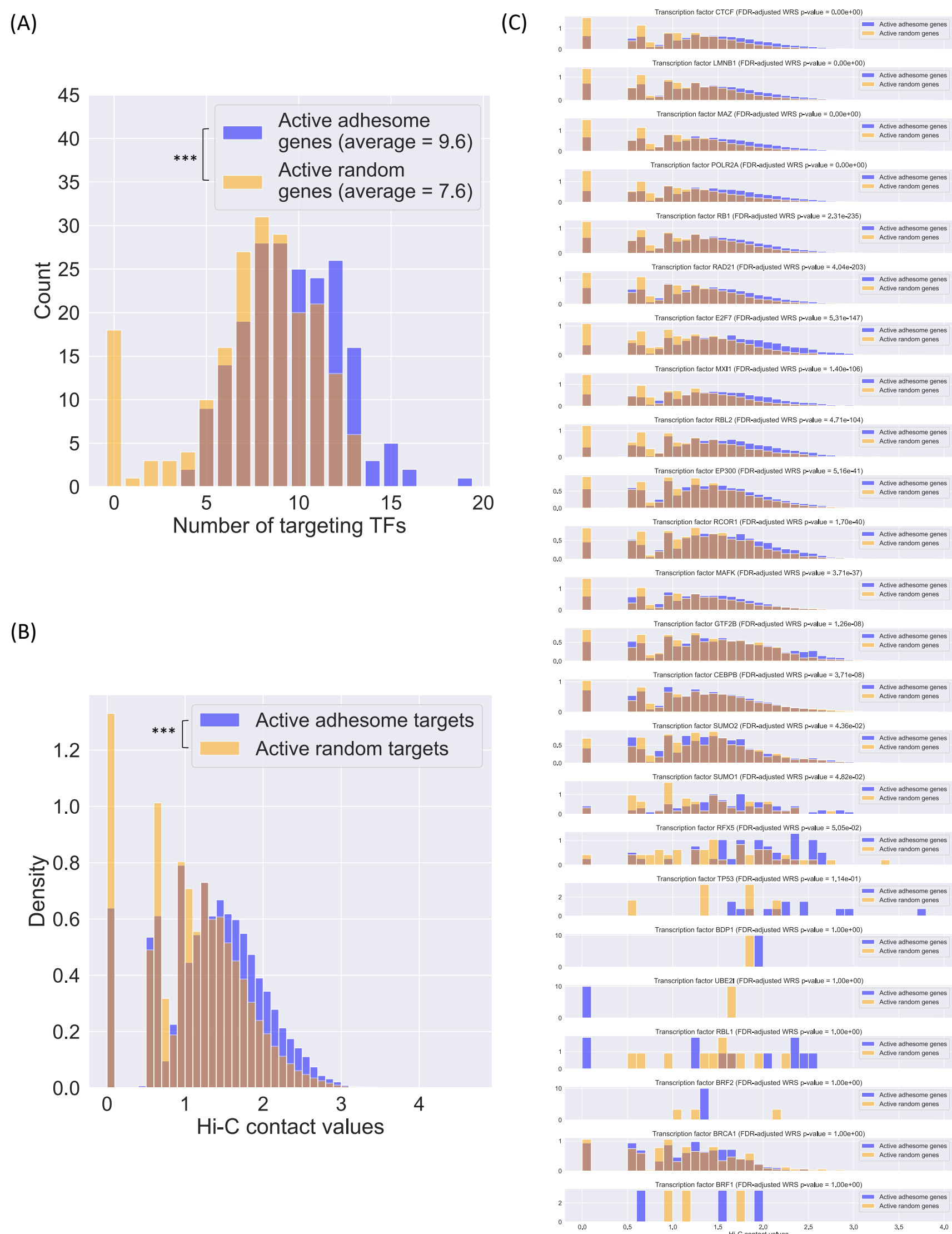


**Fig S19:** Spatial proximity analysis of transcription regulator targets. (A) Histograms of the number of TRs targeting active adhesome genes and the number of TRs targeting a set of random active non-adhesome genes (Wilcoxon Rank-Sum $p$-value $< 2 \times 10^{-10}$). (B) Histograms of interchromosomal Hi-C contacts among active adhesome TR targets and random active non-adhesome non-TR target genes located on the same chromosomes (Wilcoxon Rank-Sum test, $p$-value $< 3 \times 10^{-308}$). (C) Histograms of interchromosomal Hi-C contacts among active adhesome TR targets and among random active non-adhesome non-TR target genes located on the same chromosomes, separately for each TR. The plots are organized in ascending order of Wilcoxon Rank-Sum $p$-value.
