# Supplementary material for: Coupling between adhesome assembly and 3D chromatin organization
Source: PLoS One. 2026 Oct 29;21(10):e0361202. doi: 10.1371/journal.pone.0361202 (PMC13719029; doi:10.1371/journal.pone.0361202)
Supplement: Supplementary file 20 [file pone.0361202.s020.pdf]

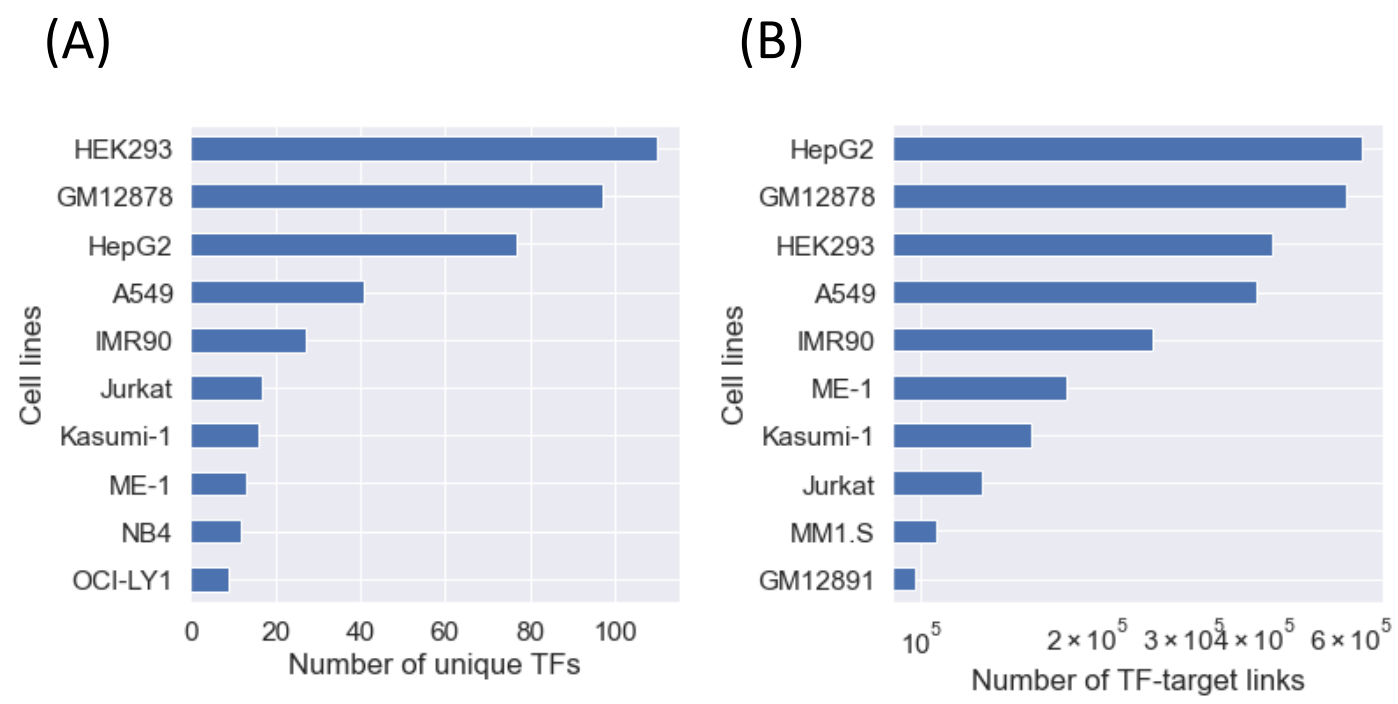


(C)

| Lung fibroblast adhesome TF | Proportion of adhesome genes targeted | | | | |
|---|---|---|---|---|---|
| | Lung fibroblasts | GM12878 | HEK293 | A549 | HepG2 |
| LMNB1 | 1.00 | 0.00 | 0.00 | 0.00 | 0.00 |
| MAZ | 0.94 | 0.43 | 0.00 | 0.00 | 0.80 |
| POLR2A | 0.89 | 0.84 | 0.00 | 0.91 | 0.88 |
| CTCF | 0.86 | 0.86 | 0.99 | 0.91 | 0.91 |
| CEBPB | 0.86 | 0.02 | 0.00 | 0.66 | 0.50 |
| RB1 | 0.77 | 0.00 | 0.00 | 0.00 | 0.00 |
| SPI1 | 0.73 | 0.81 | 0.00 | 0.00 | 0.00 |
| RAD21 | 0.73 | 0.64 | 0.00 | 0.64 | 0.97 |
| CHD1 | 0.55 | 0.00 | 0.00 | 0.00 | 0.00 |
| EP300 | 0.54 | 0.77 | 0.00 | 0.41 | 0.45 |
| MAFK | 0.46 | 0.01 | 0.00 | 0.00 | 0.46 |
| RCOR1 | 0.37 | 0.01 | 0.00 | 0.00 | 0.16 |
| E2F7 | 0.36 | 0.00 | 0.00 | 0.00 | 0.00 |
| RBL2 | 0.35 | 0.00 | 0.00 | 0.00 | 0.00 |
| MXI1 | 0.34 | 0.14 | 0.00 | 0.00 | 0.59 |
| GTF2B | 0.29 | 0.00 | 0.00 | 0.00 | 0.00 |
| SUMO2 | 0.21 | 0.00 | 0.00 | 0.00 | 0.00 |
| BRCA1 | 0.15 | 0.03 | 0.00 | 0.00 | 0.08 |
| SUMO1 | 0.05 | 0.00 | 0.00 | 0.00 | 0.00 |
| RFX5 | 0.04 | 0.03 | 0.00 | 0.00 | 0.21 |
| RBL1 | 0.02 | 0.00 | 0.00 | 0.00 | 0.00 |
| TP53 | 0.02 | 0.00 | 0.00 | 0.00 | 0.00 |
| BRF1 | 0.01 | 0.00 | 0.00 | 0.00 | 0.00 |
| UBE2I | 0.01 | 0.00 | 0.00 | 0.00 | 0.00 |
| BDP1 | 0.01 | 0.00 | 0.00 | 0.00 | 0.00 |
| BRF2 | 0.01 | 0.00 | 0.00 | 0.00 | 0.00 |
| SNAPC2 | <0.01 | 0.00 | 0.00 | 0.00 | 0.00 |
| SNAPC4 | <0.01 | 0.00 | 0.00 | 0.00 | 0.00 |
| PIAS4 | <0.01 | 0.00 | 0.00 | 0.00 | 0.00 |

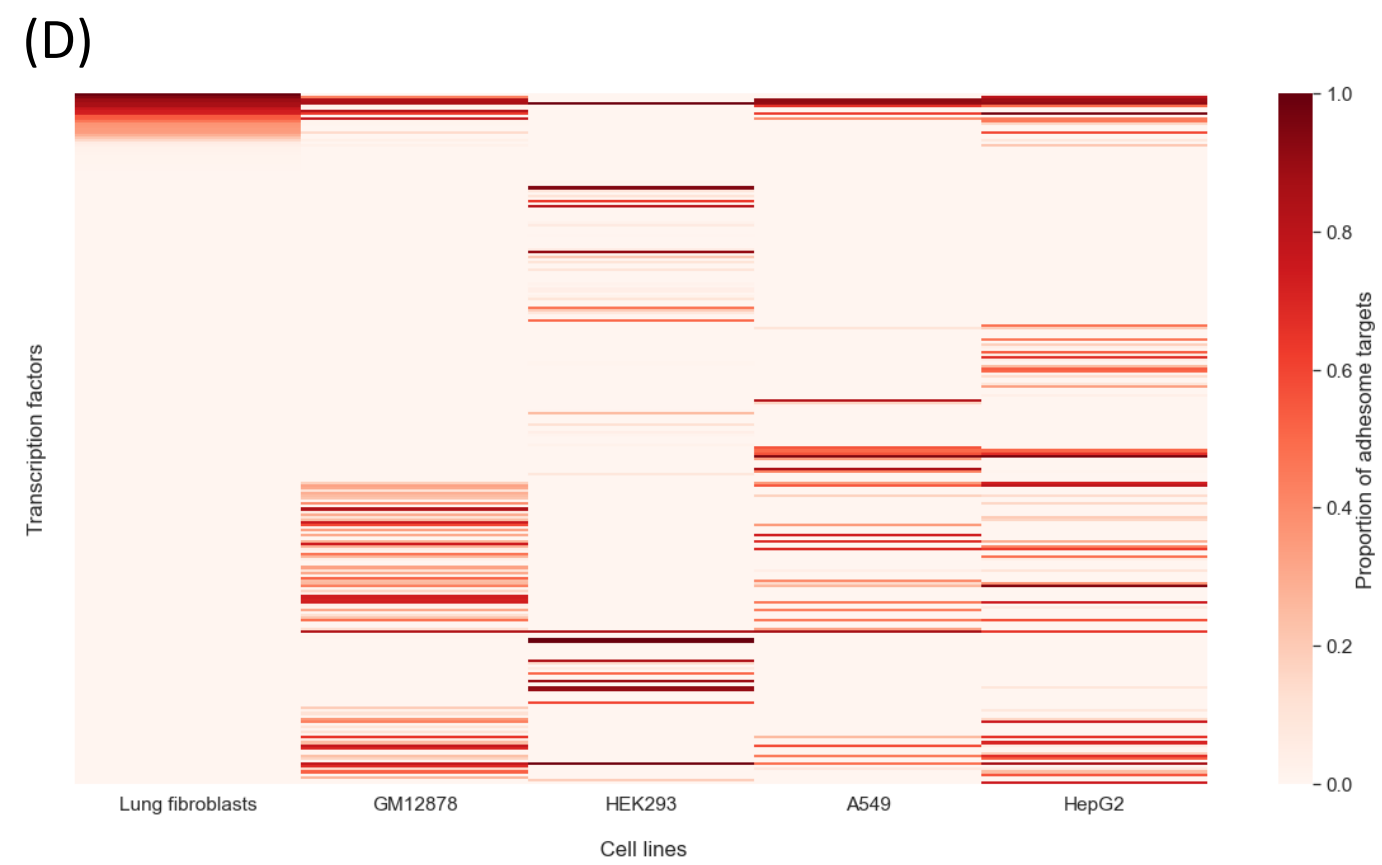


**Fig S20:** The identified active human lung fibroblast TRs target fewer adhesome genes in other cell lines and are quite specific to human lung fibroblasts. (A) Number of unique TRs present in the hTFtarget data set for different adherent and non-adherent cell lines. (B) Number of unique TR-target links present in the hTFtarget data set for different cell lines. The cell lines HepG2, GM12878, HEK293, A549 and normal lung fibroblasts (combination of IMR-90, WI38, AG04450, HPF and NHLF) have the highest amount of data. (C) Table showing the proportion of adhesome genes targeted by selected lung fibroblast TRs in different cell lines, including lung fibroblasts, HepG2, GM12878, HEK293, and A549. Rows shaded in red correspond to the 13 significant TRs identified in lung fibroblasts. (D) Heatmap of the proportion of adhesome genes targeted by TRs in different cell lines compared to the proportion of adhesome genes targeted in normal lung fibroblasts. These include cell lines with adherence properties (A549, HepG2, HEK293) and a non-adherent cell line (GM12878). TRs that target (active and/or inactive) adhesome genes in IMR-90 are in the top 29 rows. The remaining TRs do not target adhesome genes in IMR-90 but target at least one adhesome gene in other cell lines.
