# Supplementary material for: Coupling between adhesome assembly and 3D chromatin organization
Source: PLoS One. 2026 Oct 29;21(10):e0361202. doi: 10.1371/journal.pone.0361202 (PMC13719029; doi:10.1371/journal.pone.0361202)
Supplement: Supplementary file 21 [file pone.0361202.s021.pdf]

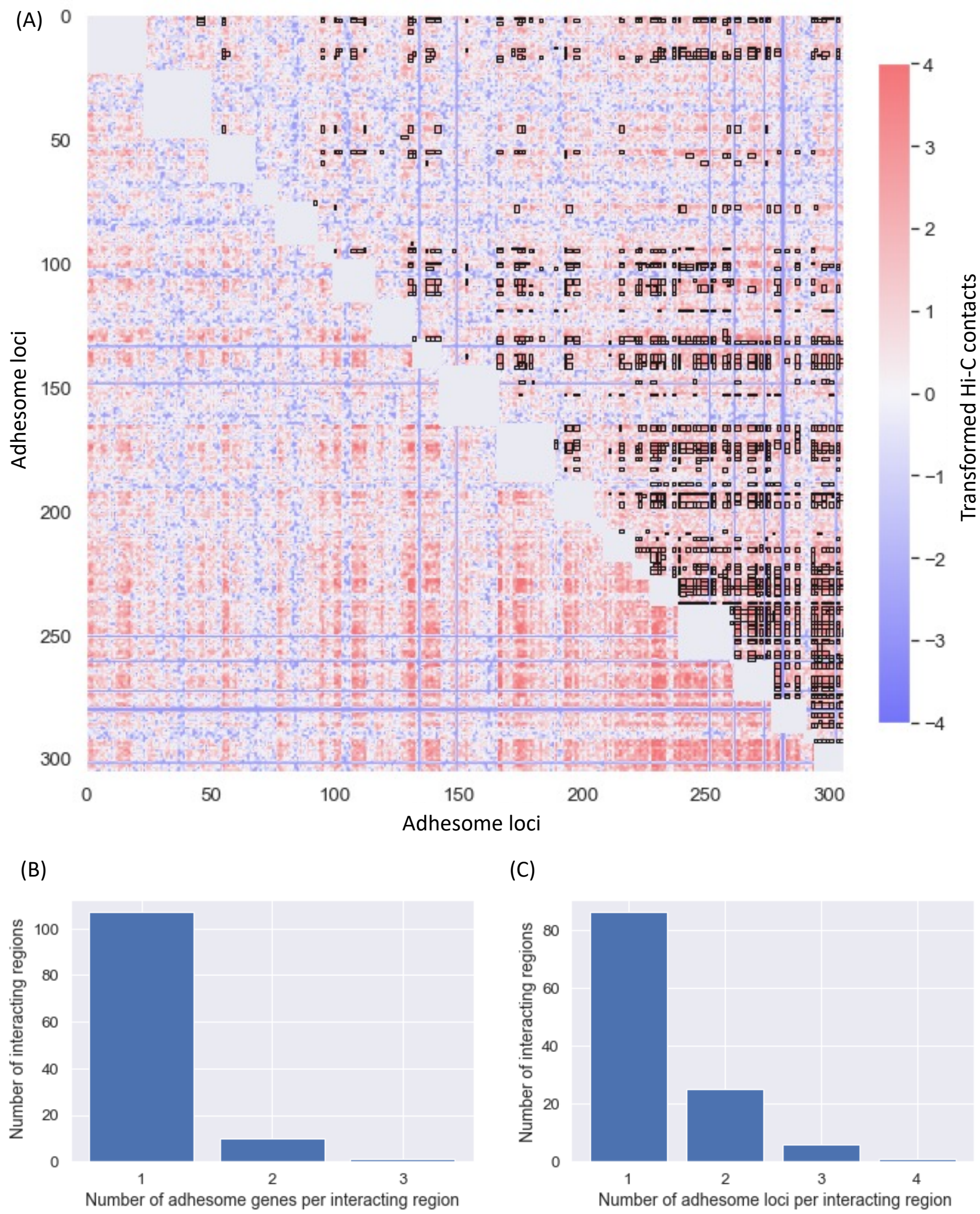

Fig S21: Projection of LAS submatrices onto adhesome loci. (A) Heatmap of transformed Hi-C contact frequencies among active adhesome and adhesome TR loci (frequencies were log-transformed and centerdized using the mean and standard deviation of contact values across the entire genome). Significant submatrices identified by the LAS algorithm and whose average Hi-C value is higher than 2 are shown as black boxes. This heatmap is obtained by combining LAS interacting regions containing adhesome genes from all interchromosomal Hi-C contact matrices (such as, for example, the matrix in main Fig. 4A). (B) Bar plot of the number of adhesome genes per adhesome interacting region. Adhesome LAS interacting regions contain at most 3 adhesome genes. (C) Bar plot of the number of adhesome loci per adhesome interacting region. Adhesome LAS interacting regions contain at most 4 adhesome loci. To relate this to (B), note that an adhesome gene may span multiple 250 kb loci.
