# Supplementary material for: Coupling between adhesome assembly and 3D chromatin organization
Source: PLoS One. 2026 Oct 29;21(10):e0361202. doi: 10.1371/journal.pone.0361202 (PMC13719029; doi:10.1371/journal.pone.0361202)
Supplement: Supplementary file 22 [file pone.0361202.s022.pdf]

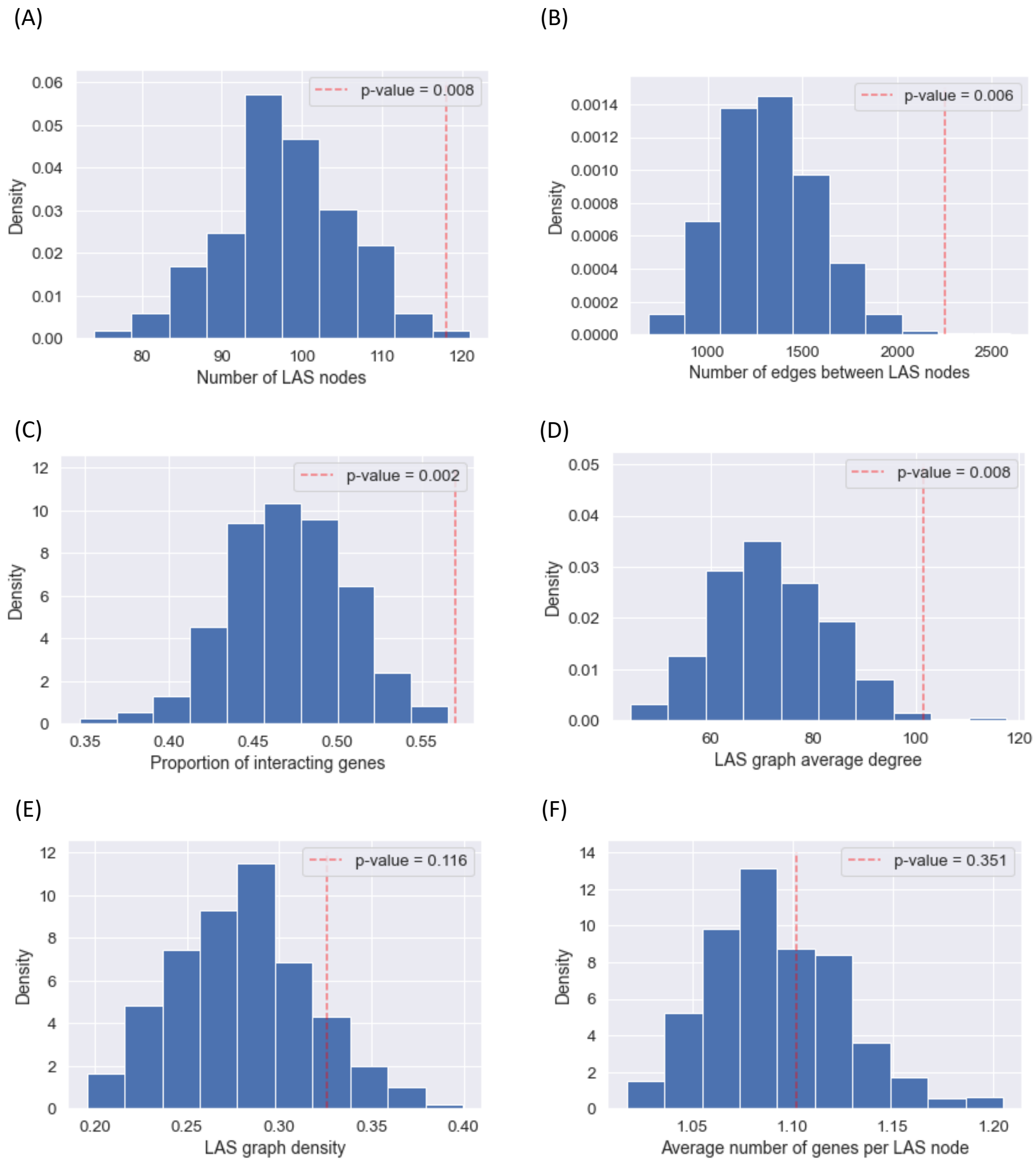

Fig S22: Statistical significance of the LAS adhesome network. (A) Histogram of the number of LAS nodes containing at least one gene from a collection of 500 random gene sets. Each random gene set is sampled uniformly without replacement among all active non-adhesome genes. The number of nodes containing active adhesome and adhesome TR genes is shown with a red dashed vertical line, corresponding to a p-value of 0.008. This shows that a significantly high number of nodes contain adhesome genes under the 1% significance level. (B) Histogram of the number of interactions among LAS nodes containing at least one gene from the collection of 500 random gene sets in (A). The number of interactions among adhesome LAS nodes is shown with a red dashed vertical line, corresponding to a p-value of 0.006. This shows that there is a significantly high number of interactions between adhesome LAS nodes at the 1% significance level. (C) Histogram of the proportion of genes belonging to LAS nodes from the collection of 500 random gene sets in (A). The proportion of adhesome genes belonging to adhesome LAS nodes is shown with a red dashed vertical line, corresponding to a p-value of 0.002. This shows that there is a significantly high proportion of adhesome genes that belong to adhesome LAS nodes at the 1% significance level. (D) Histogram of average degree computed on networks of LAS nodes containing genes from the collection of 500 random gene sets in (A). The average degree of the adhesome LAS network is shown with a red dashed vertical line, corresponding to a p-value of 0.008. This shows that, on average, the degree of adhesome LAS nodes is significantly higher than the degree of LAS nodes containing random genes at the 1% significance level. (E) Histogram of graph density computed on networks of LAS nodes containing genes from the collection of 500 random gene sets in (A). The graph density of the adhesome LAS network is shown with a red dashed vertical line, corresponding to a p-value of 0.116. (F) Histogram of the average number of genes per LAS node from the collection of 500 random gene sets in (A); only LAS nodes containing at least one gene from the random gene set at hand are considered. The average number of adhesome genes per adhesome LAS node is shown with a red dashed vertical line, corresponding to a p-value of 0.351.
