# Supplementary material for: Coupling between adhesome assembly and 3D chromatin organization
Source: PLoS One. 2026 Oct 29;21(10):e0361202. doi: 10.1371/journal.pone.0361202 (PMC13719029; doi:10.1371/journal.pone.0361202)
Supplement: Supplementary file 23 [file pone.0361202.s023.pdf]

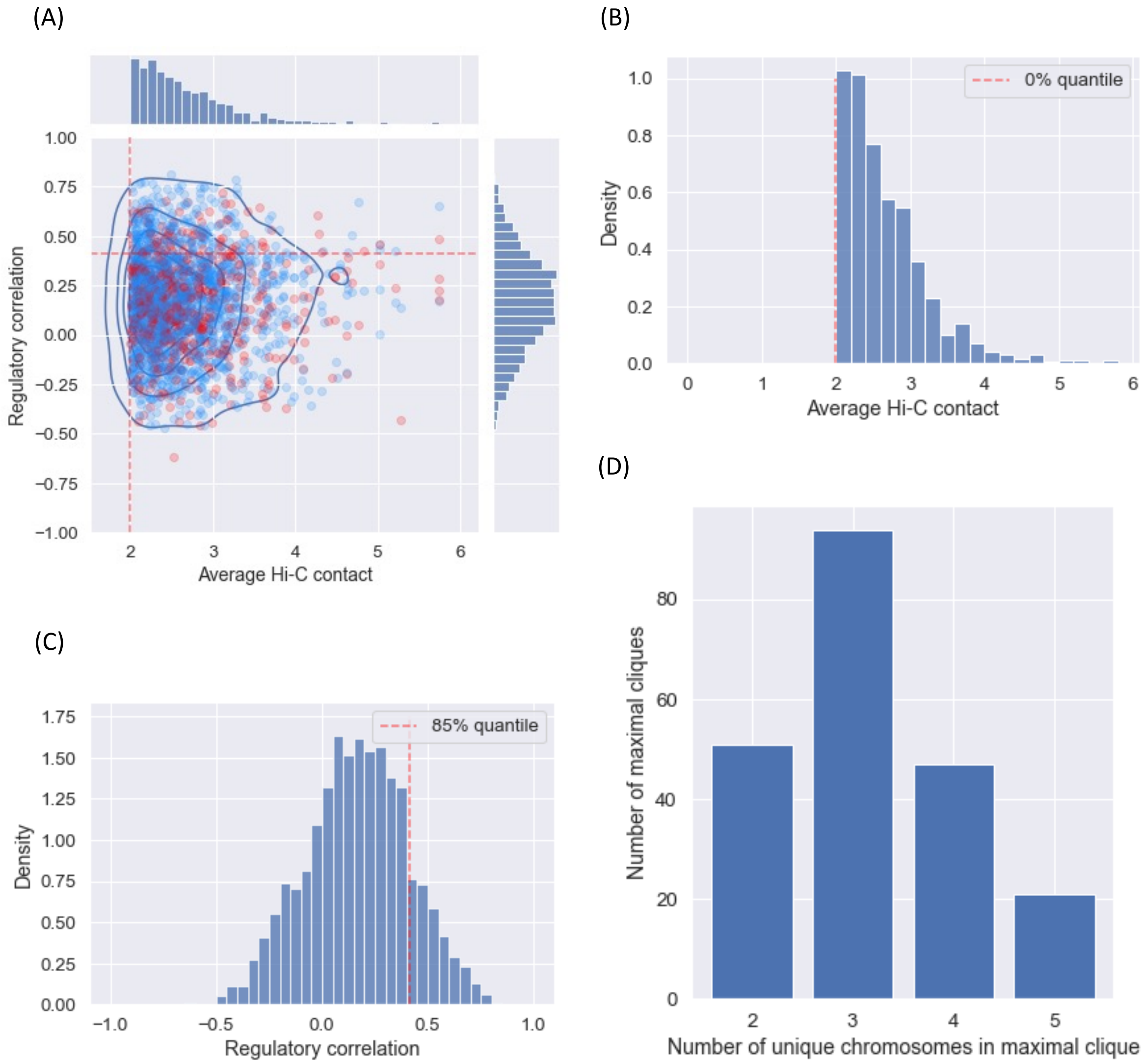

Fig S23: Regulatory correlation of adhesome LAS nodes. (A) Scatterplot and 2D contour plot of pairs of adhesome LAS nodes. The location of a given pair (u, v) on the scatterplot is determined by the average Hi-C contact value (x-axis) and the regulatory correlation (y-axis) associated with the edge u–v of the adhesome LAS network. Pairs that involve at least one TR targeting active adhesome genes are shown in red. The red dashed lines correspond to marginal thresholds on the distribution of average Hi-C contact values (vertical, 0% quantile) and regulatory correlations (horizontal, 85% quantile). (B) Histogram of average transformed Hi-C contact values among all adhesome LAS nodes. The red dashed vertical line corresponds to the minimum value, which is equal to 2 (by design from the LAS thresholding we performed in a previous step, see Supplementary Fig. S7C). (C) Histogram of average Spearman regulatory correlation among all adhesome LAS nodes. The average Spearman regulatory correlation between two adhesome LAS nodes is computed by taking the average of Spearman regulatory correlations between all tuples of adhesome genes in the two nodes, using the 48 regulatory features used throughout this work. The red dashed vertical line corresponds to the 85% quantile, used as a lower regulatory threshold to select interactions among adhesome LAS nodes for the adhesome LAS network of main Fig. 4B. (D) Bar plot of the number of unique chromosomes per maximal clique in the adhesome LAS network obtained after dropping interactions whose average Spearman regulatory correlation is below the threshold in (C). Maximal cliques contain at most 5 unique chromosomes, which is consistent with the hypothesis that no more than 5 chromosomes can physically interact at a given location.
