# Supplementary material for: Coupling between adhesome assembly and 3D chromatin organization
Source: PLoS One. 2026 Oct 29;21(10):e0361202. doi: 10.1371/journal.pone.0361202 (PMC13719029; doi:10.1371/journal.pone.0361202)
Supplement: Supplementary file 24 [file pone.0361202.s024.pdf]

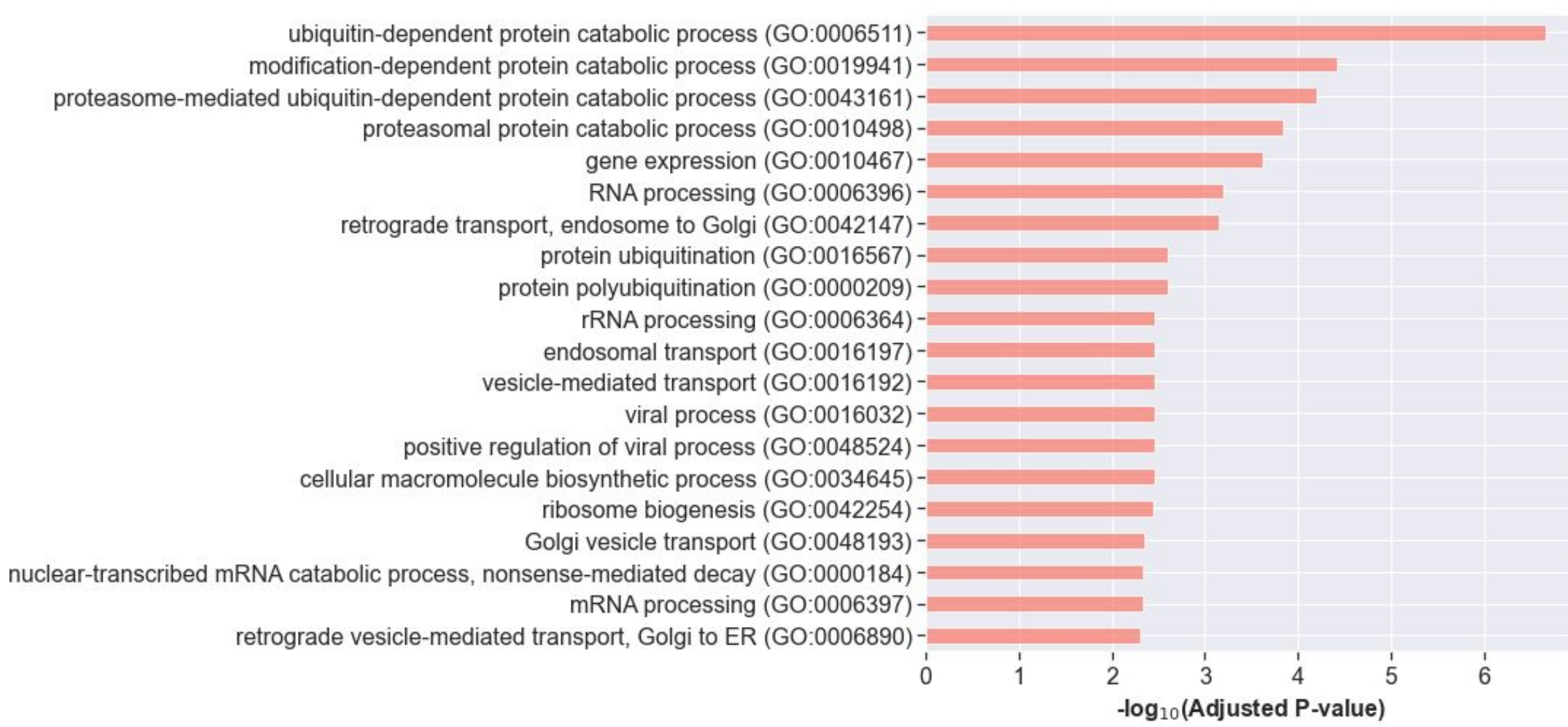

Fig S24: Significant Gene Ontology (GO) terms associated to active non-adhesome genes belonging to genomic regions that interact with adhesome interacting regions. Only genes having a strong positive regulatory correlation (upper decile) with the adhesome genes they interact with (in LAS) are considered. A GO term is reported as significant if its FDR-adjusted p-value is lower than 10^{-2}. Many of the identified GO terms are associated with the ubiquitin-proteasome system.
