# Supplementary material for: Coupling between adhesome assembly and 3D chromatin organization
Source: PLoS One. 2026 Oct 29;21(10):e0361202. doi: 10.1371/journal.pone.0361202 (PMC13719029; doi:10.1371/journal.pone.0361202)
Supplement: Supplementary file 25 [file pone.0361202.s025.pdf]

(A)

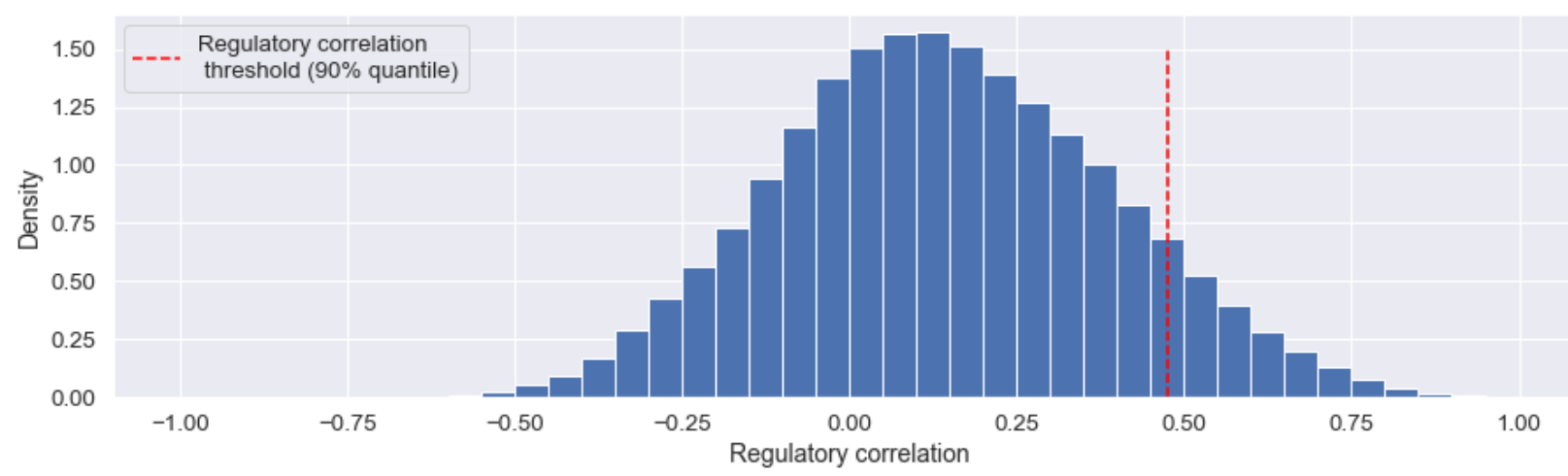

(B)

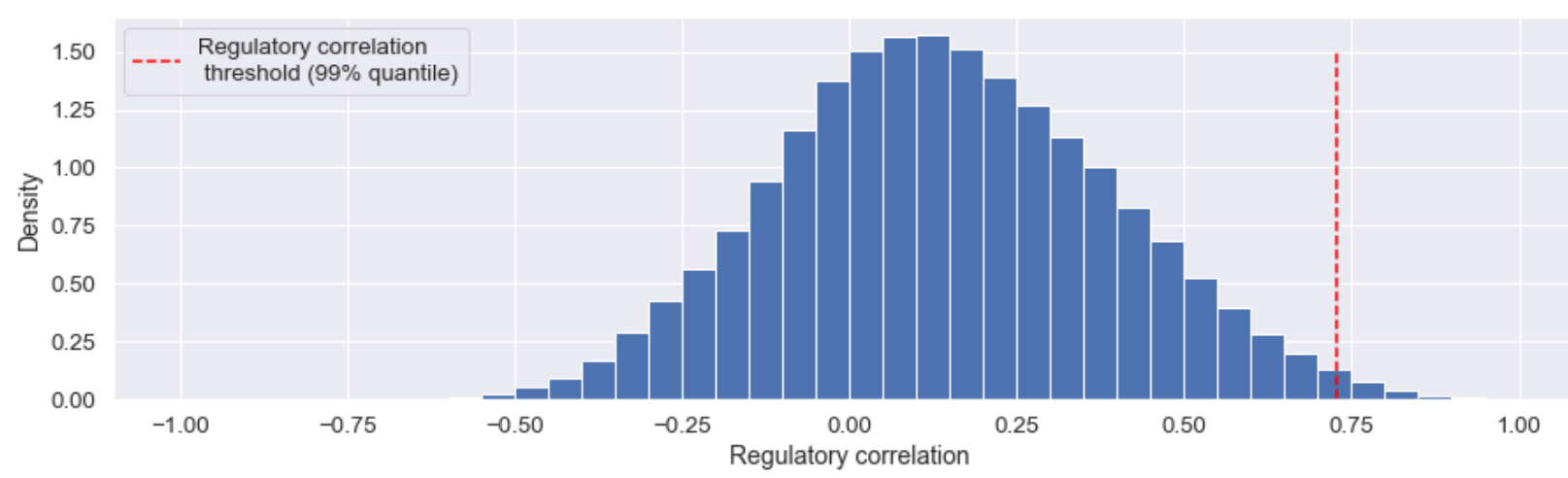

(C)

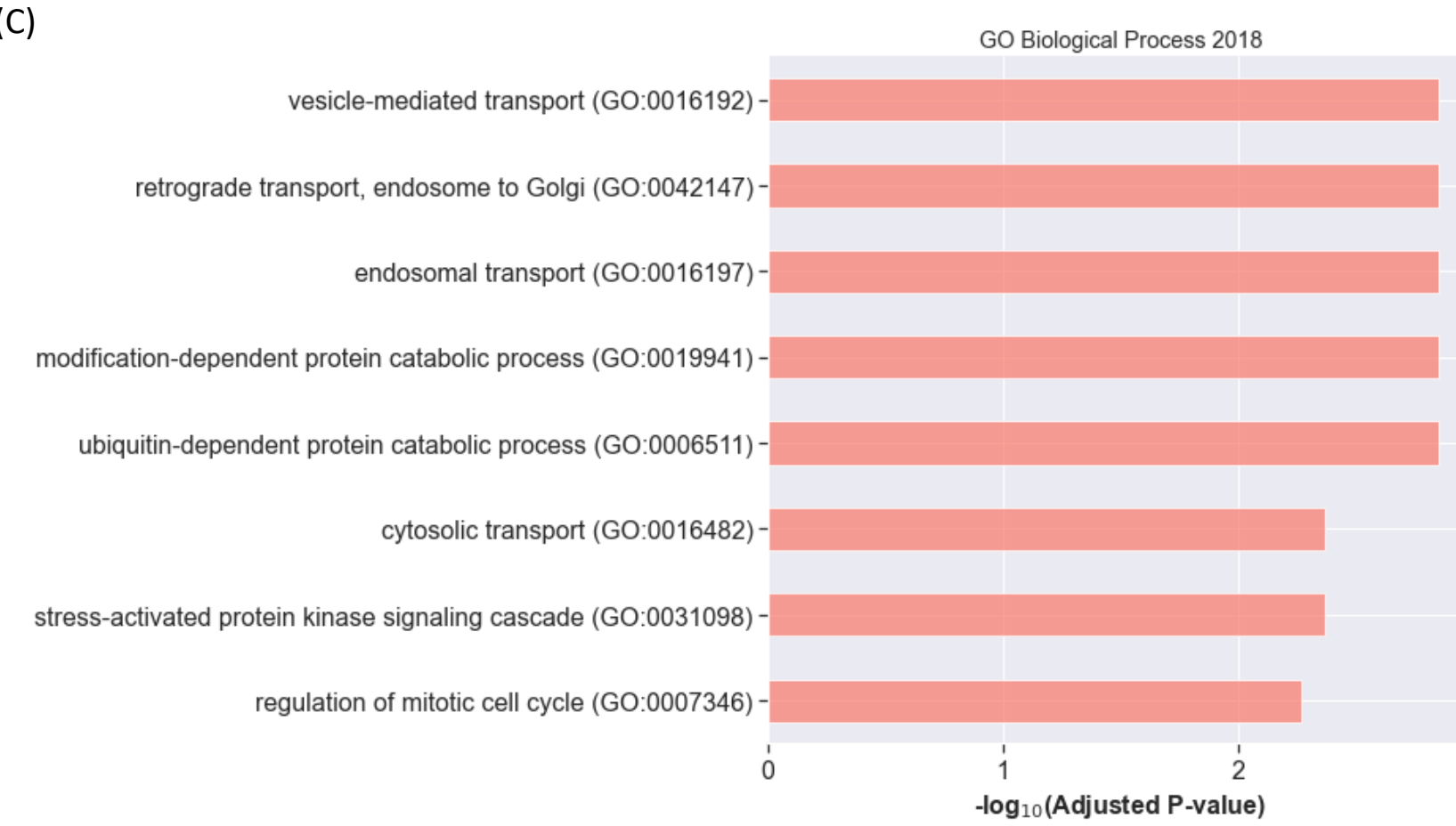

Fig S25: Gene set enrichment analysis of active non-adhesome genes interacting with active adhesome genes. (A) Histogram of Spearman regulatory correlation between (i) active non-adhesome genes in adhesome interacting regions and (ii) the active adhesome genes with which these genes interact interchromosomally. Regulatory correlation is computed from the 48 regulatory features used throughout this work. The red dashed vertical threshold line marks the upper decile of the distribution. (B) Same as A, with the red dashed vertical threshold line marking the upper percentile of the distribution. (C) Significant Gene Ontology (GO) terms associated to active non-adhesome genes belonging to genomic regions that interact with adhesome interacting regions. Only genes having a strong positive regulatory correlation (upper percentile in B) with the active adhesome genes they interact with (in LAS) are considered. A GO term is reported as significant if its FDR-adjusted p-value is lower than 10^{-2}.
