# Supplementary material for: Coupling between adhesome assembly and 3D chromatin organization
Source: PLoS One. 2026 Oct 29;21(10):e0361202. doi: 10.1371/journal.pone.0361202 (PMC13719029; doi:10.1371/journal.pone.0361202)
Supplement: Supplementary file 26 [file pone.0361202.s026.pdf]

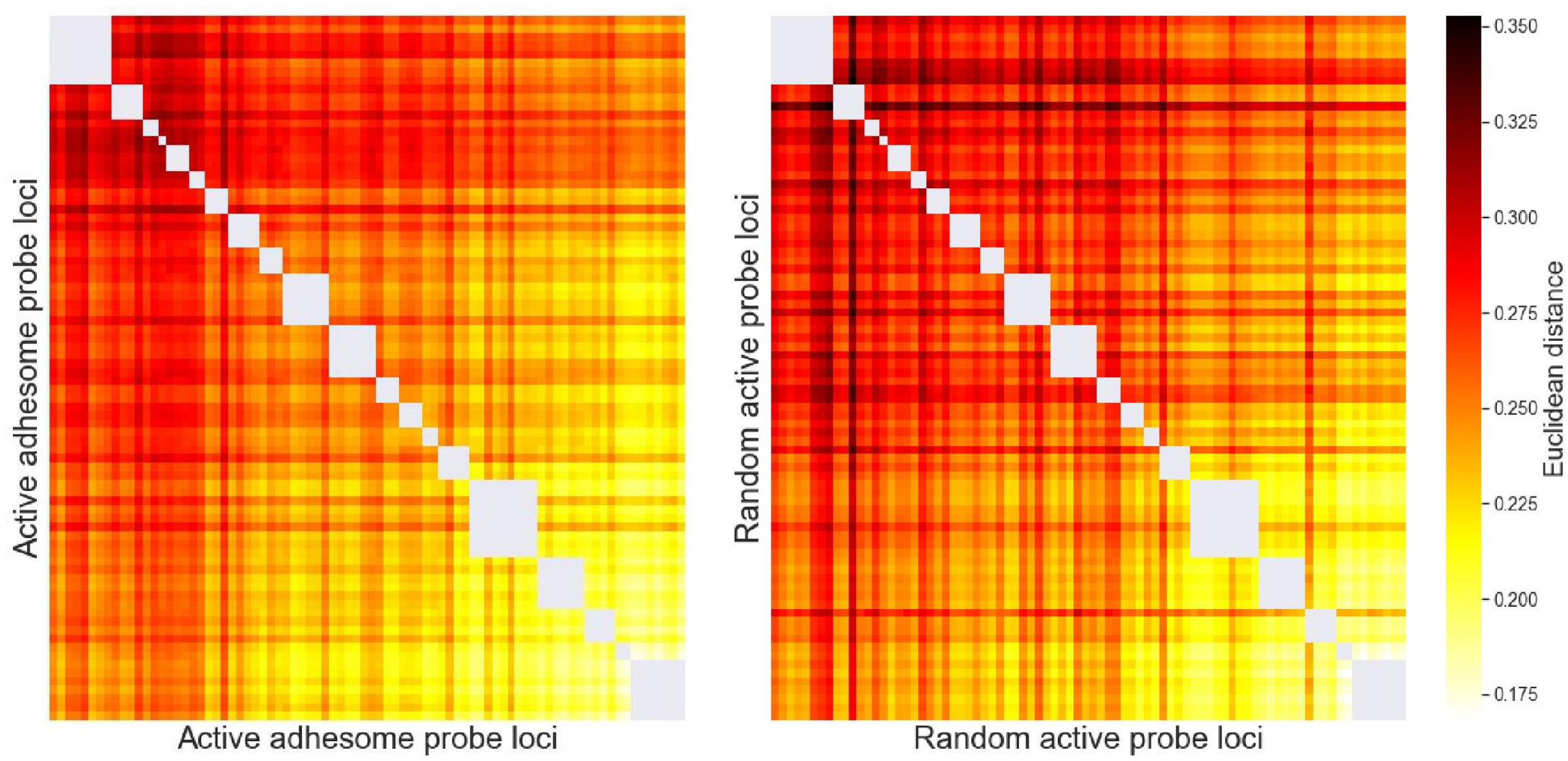

Fig S26: Left: Heatmap of cell-normalized MERFISH Euclidean distance between active adhesome probe loci. Loci are ordered according to chromosome and genomic position. Cell-normalized Euclidean distances between loci i and j are averaged over all cells containing probes i and j. Cell-normalization corresponds to dividing the physical distance between i and j in cell c by the maximum probe-probe distance in that cell. Right: Heatmap of Euclidean distance between selected random non-adhesome loci. The number of random probe loci per chromosome is chosen equal to that of adhesome probe loci, to allow for a fair comparison. Probe loci were mapped within 500 kb of the corresponding genes (Methods).
