# Supplementary material for: Coupling between adhesome assembly and 3D chromatin organization
Source: PLoS One. 2026 Oct 29;21(10):e0361202. doi: 10.1371/journal.pone.0361202 (PMC13719029; doi:10.1371/journal.pone.0361202)
Supplement: Supplementary file 27 [file pone.0361202.s027.pdf]

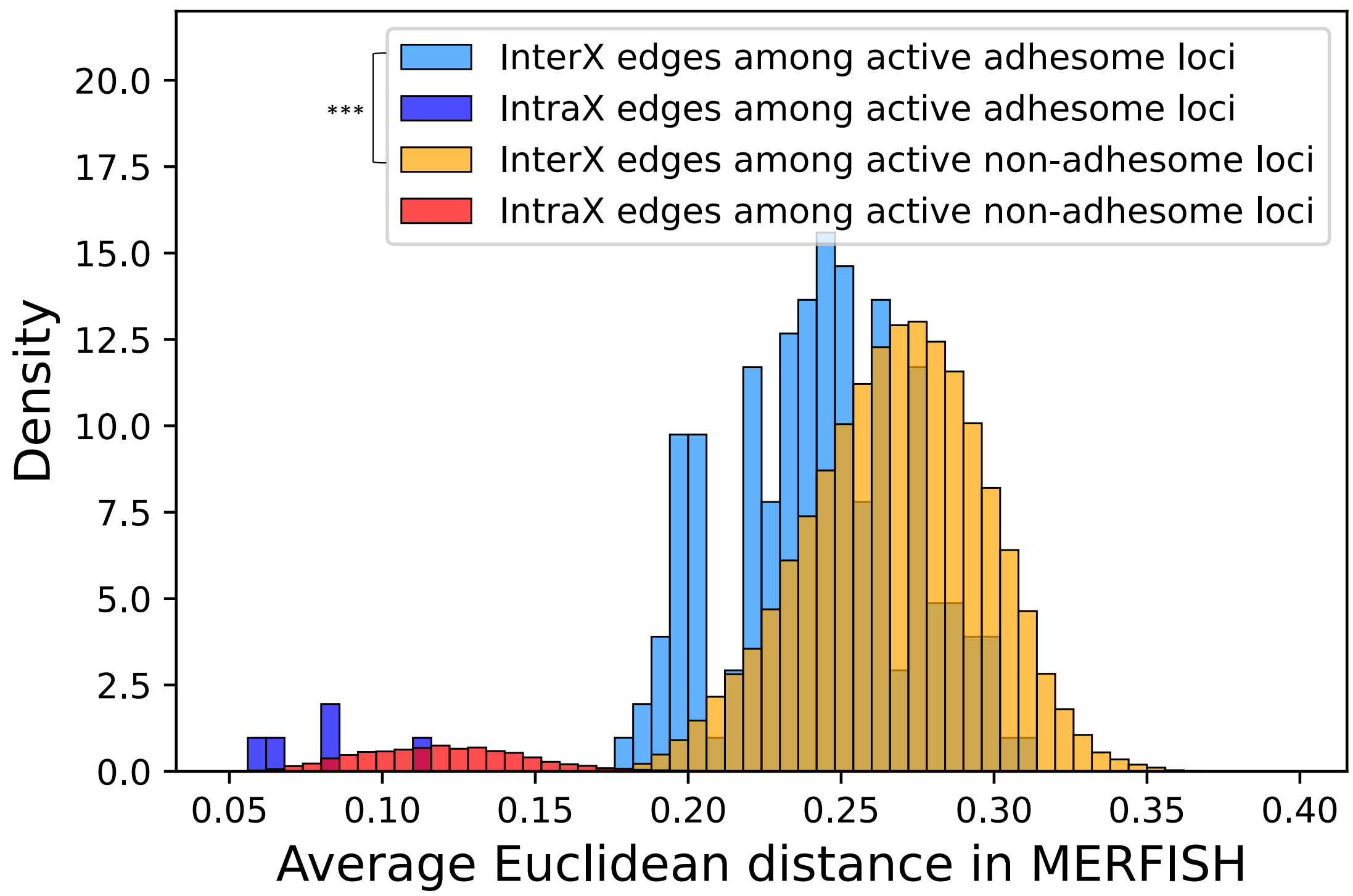

Fig S27: Extended MERFISH analysis. Distribution of average normalized Euclidean distances between active adhesome gene probes and active non-adhesome gene probes (a MERFISH probe is associated with a gene if it overlaps with the gene). The average interchromosomal Euclidean distance between active adhesome probes is smaller than the average interchromosomal Euclidean distance among active non-adhesome probes (Wilcoxon Rank-Sum p-value $< 1 \times 10^{-18}$).
