# Supplementary material for: Coupling between adhesome assembly and 3D chromatin organization
Source: PLoS One. 2026 Oct 29;21(10):e0361202. doi: 10.1371/journal.pone.0361202 (PMC13719029; doi:10.1371/journal.pone.0361202)
Supplement: Supplementary file 28 [file pone.0361202.s028.pdf]

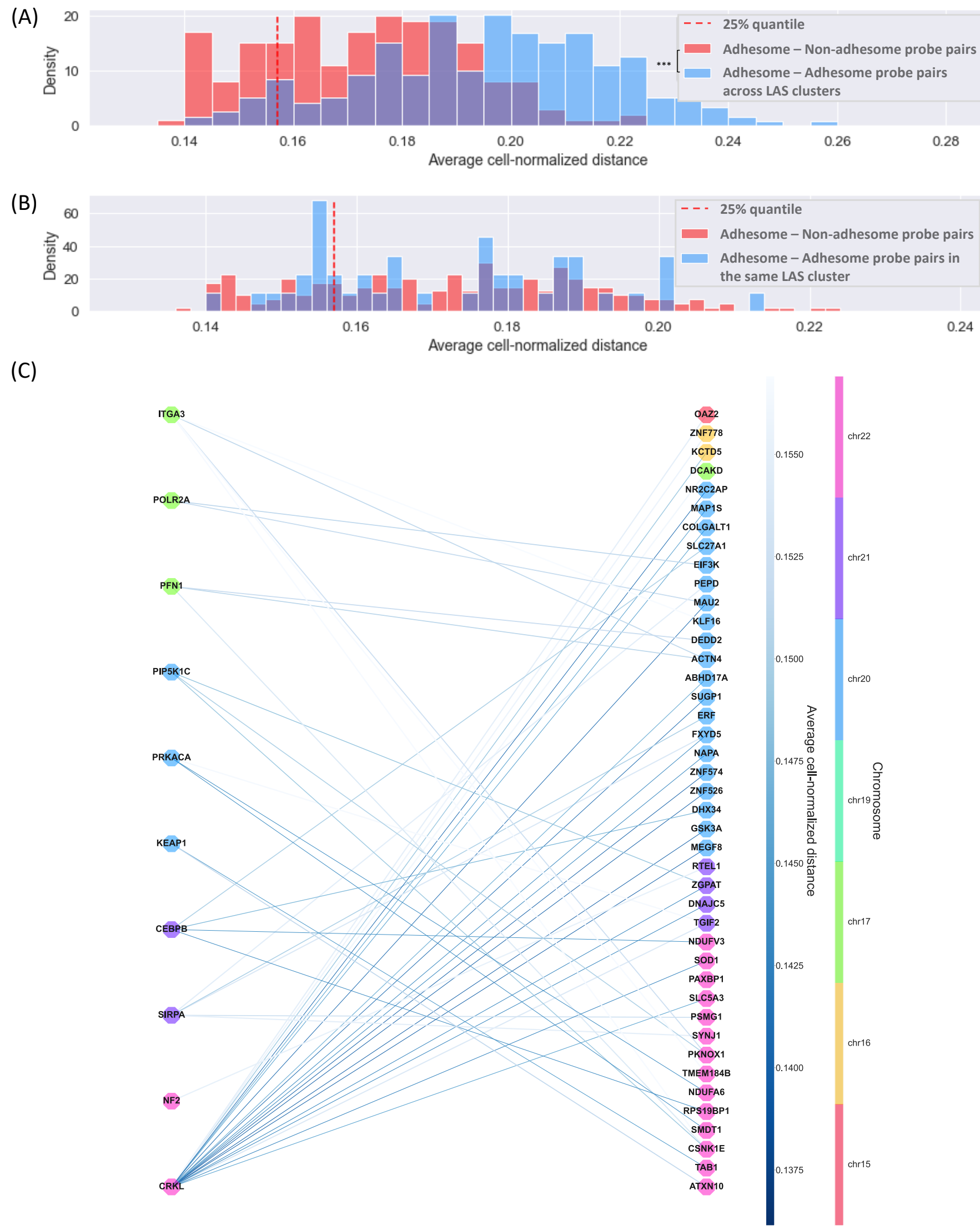

Fig S28: Bipartite network of active non-adhesome genes interacting with active adhesome genes. (A) Red histogram of the average cell-normalized Euclidean distance between 135 active non-adhesome genes (61 MERFISH probes) and the 17 active adhesome genes (16 MERFISH probes) they interact with. MERFISH probes are associated with a gene if they are less than 100 kb upstream or downstream of the gene. Only gene pairs that interact in LAS and have a strong regulatory correlation (upper decile in Supplementary Fig. S25A) are considered. The red vertical dashed line corresponds to an upper threshold (first quartile) on the distance used to select edges for (C). Blue histogram of the average cell-normalized Euclidean distance between 23 active adhesome MERFISH probes across LAS regions, used for comparison with the red histogram (Wilcoxon Rank Sums test, p-value $< 2 \times 10^{-29}$). (B) Red histogram as in (A). Blue histogram of the average cell-normalized Euclidean distance between 13 active adhesome MERFISH probes (only LAS interactions are reported) (Wilcoxon Rank Sums test, p-value=0.75). (C) Bipartite network of active adhesome genes (10 genes, left) and active non-adhesome genes they interact with (42 genes, right). Only genes with an associated MERFISH probe (less than 100 kb upstream or downstream) are considered; more details on how this network is constructed can be found in the main text. Gene colors indicate the corresponding chromosomes. Edge colors and thicknesses reflect the physical distance between genes. Edges with an average cell-normalized distance above the first quartile threshold of (A) are not drawn.
