# Supplementary material for: Coupling between adhesome assembly and 3D chromatin organization
Source: PLoS One. 2026 Oct 29;21(10):e0361202. doi: 10.1371/journal.pone.0361202 (PMC13719029; doi:10.1371/journal.pone.0361202)
Supplement: Supplementary file 29 [file pone.0361202.s029.pdf]

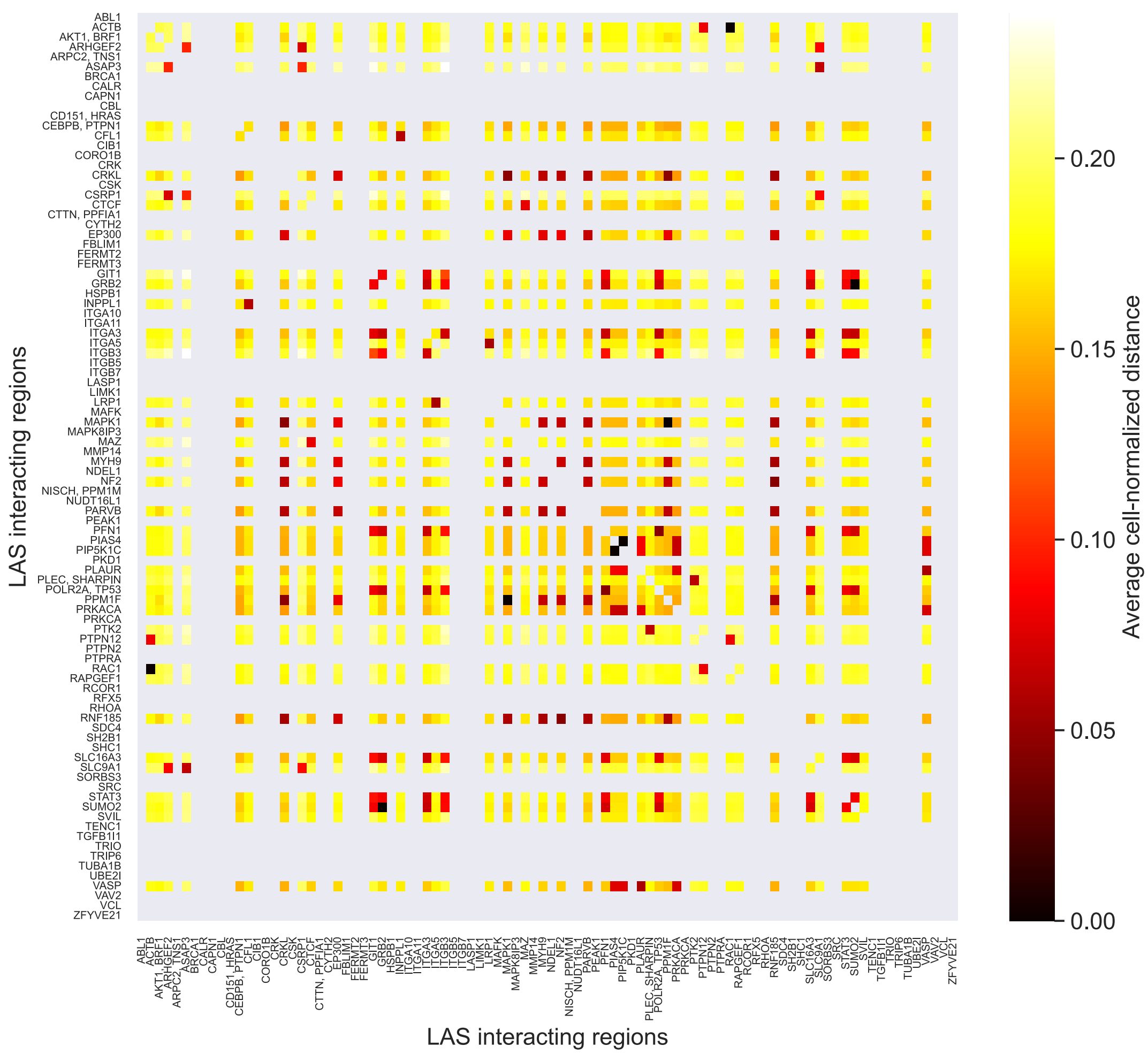

Fig S29: Relationship between Hi-C contact frequency and DNA-MERFISH Euclidean distance between adhesome loci. Heatmap of average cell-normalized DNA-MERFISH Euclidean distance between adhesome LAS nodes. The distance between two nodes is defined by averaging the distances between all DNA-MERFISH probes associated to the first node and all DNA-MERFISH probes associated to the second node. A DNA-MERFISH probe is associated to a given adhesome LAS node if it is located at most 500 kb upstream or downstream of an adhesome gene within that node. Adhesome LAS nodes that do not have an associated DNA-MERFISH probe are represented as grey rows/columns in the heatmap (missing values). Self-distances are also shown in grey. Dark entries (very low distance) typically correspond to pairs of adhesome LAS nodes on the same chromosome.
