# Supplementary material for: Coupling between adhesome assembly and 3D chromatin organization
Source: PLoS One. 2026 Oct 29;21(10):e0361202. doi: 10.1371/journal.pone.0361202 (PMC13719029; doi:10.1371/journal.pone.0361202)
Supplement: Supplementary file 30 [file pone.0361202.s030.pdf]

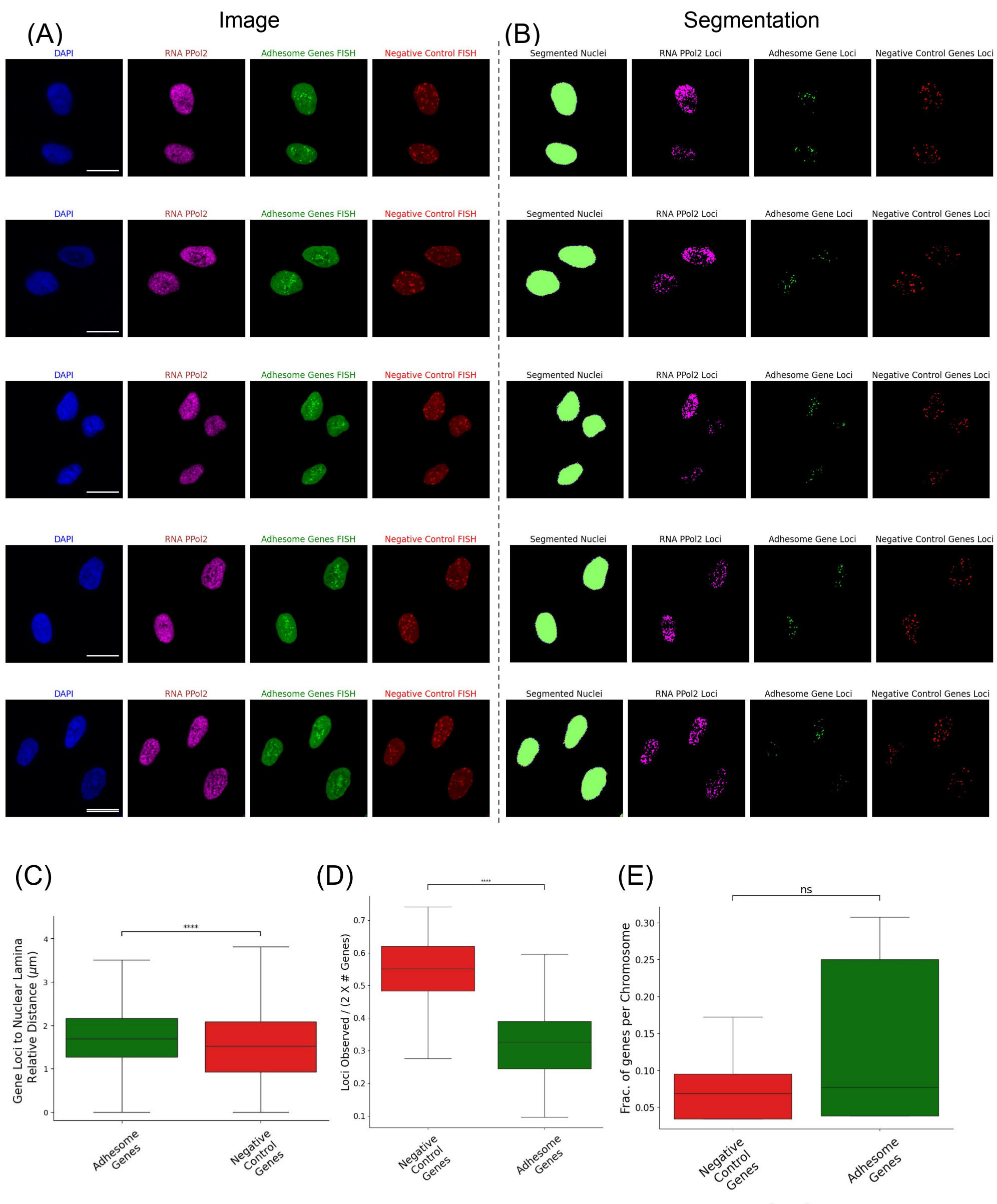

(A)
Image
(B)
Segmentation
DAPI
RNA PPol2
Adhesome Genes FISH
Negative Control FISH
Segmented Nuclei
RNA PPol2 Loci
Adhesome Gene Loci
Negative Control Genes Loci
(C)
Gene Loci to Nuclear Lamina Relative Distance (μm)
Adhesome Genes
Negative Control Genes
****
(D)
Loci Observed / (2 X # Genes)
Negative Control Genes
Adhesome Genes
(E)
Frac. of genes per Chromosome
ns
Negative Control Genes
Adhesome Genes
Gene Groups

(F)

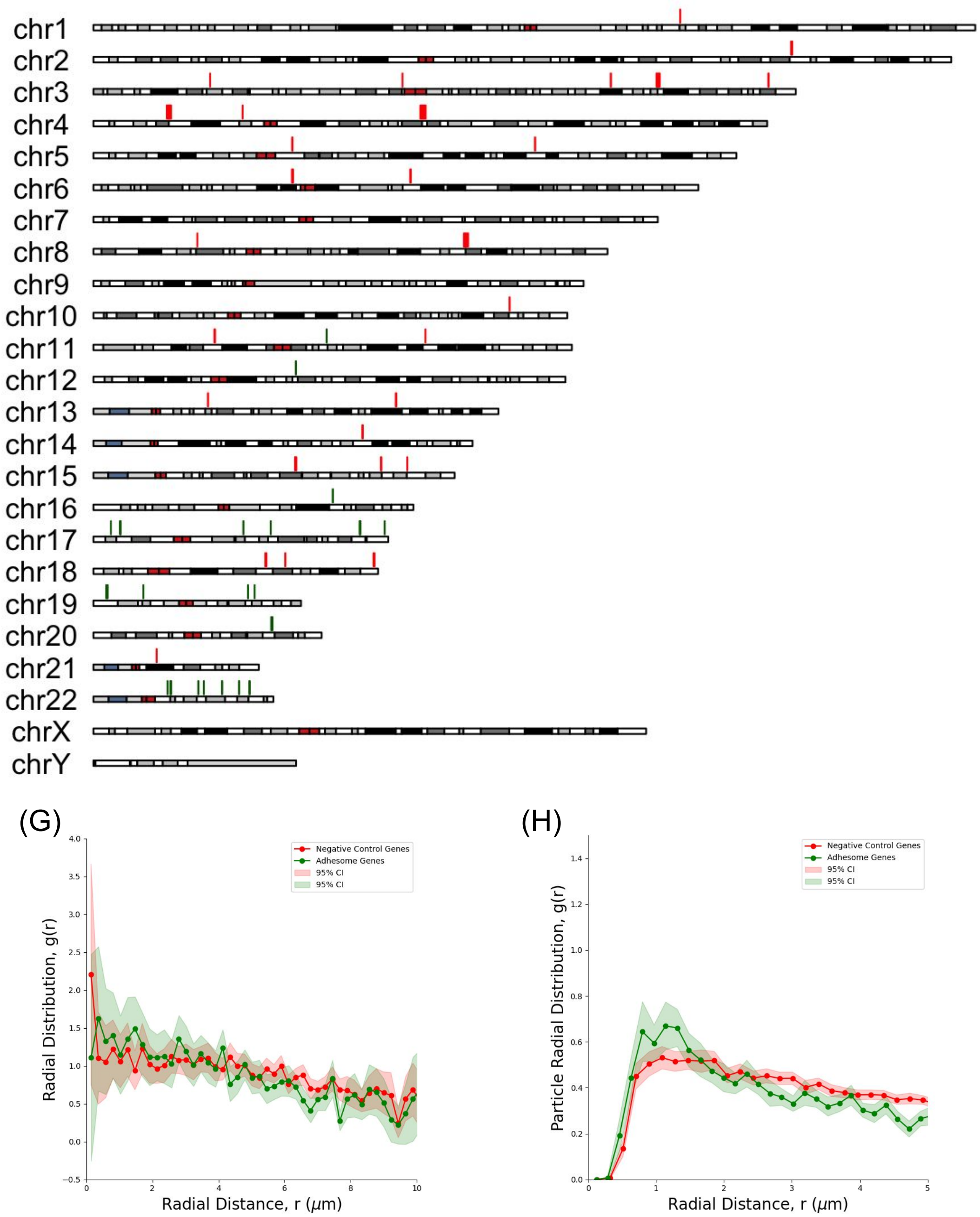

Fig S30: Representative examples of (A) max z projected confocal microscopy images labelled with DAPI, pRNA POL2, and DNA-FISH probes to core adhesome genes and negative control genes, and (B) segmented nuclei, adhesome and negative control gene loci masks. (C) Box plots show the median shortest distance between adhesome gene loci and lamina against negative control gene loci (N (biological replicates) = 2, $\geq$ 100 nuclei per replicate). (D) Box plot shows the distinct loci of the adhesome gene observed in each nucleus against the negative control (N (biological replicates) = 2, $\geq$ 100 nuclei per replicate). (E) Box plot shows the fraction of adhesome genes on each chromosome against negative control genes. (F) The karyogram of adhesome gene loci (green) and negative control gene loci (Red). (G) The radial distribution function represents the density of particles from the nuclear geometric centre. (H) The particle radial distribution function describes how the density of segmented gene loci varies as a function of distance from each locus's centroid. Here, the PRDF of adhesome genes is represented in green, and the PRDF of negative control genes is represented in red. Scale bars: 20 μm.
