# Supplementary material for: Coupling between adhesome assembly and 3D chromatin organization
Source: PLoS One. 2026 Oct 29;21(10):e0361202. doi: 10.1371/journal.pone.0361202 (PMC13719029; doi:10.1371/journal.pone.0361202)
Supplement: Supplementary file 31 [file pone.0361202.s031.pdf]

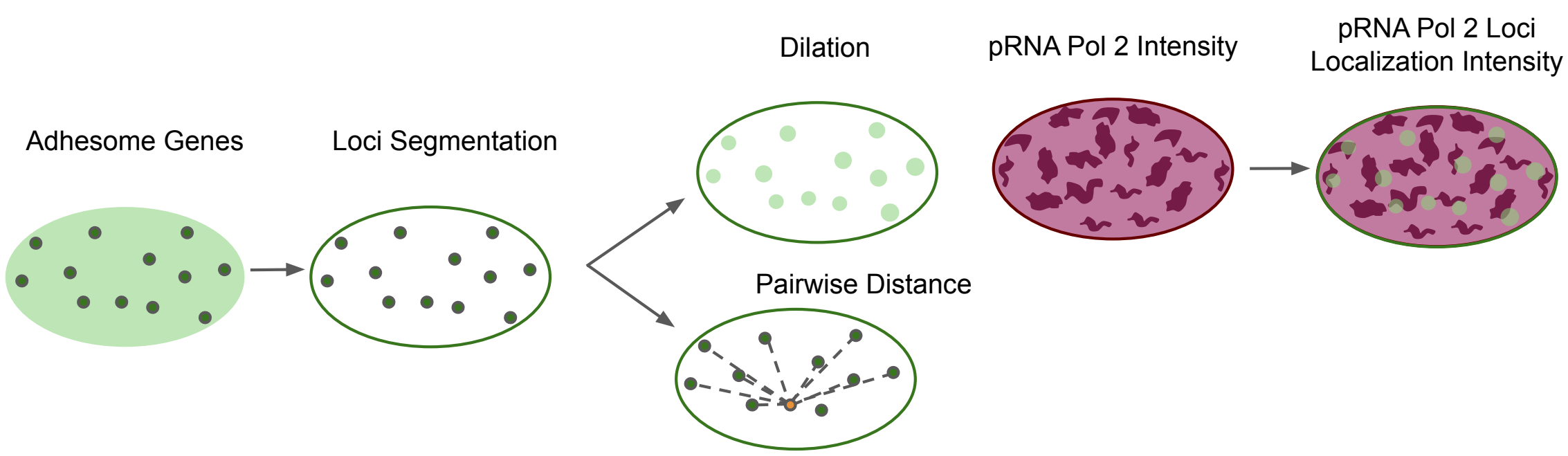

Fig S31: Schematic representation of the segmentation of adhesome loci, corresponding active transcription sites and evaluation of adhesome loci distribution.
