# Supplementary material for: Coupling between adhesome assembly and 3D chromatin organization
Source: PLoS One. 2026 Oct 29;21(10):e0361202. doi: 10.1371/journal.pone.0361202 (PMC13719029; doi:10.1371/journal.pone.0361202)
Supplement: Supplementary file 32 [file pone.0361202.s032.pdf]

Supplementary Results:

Thresholded adhesome network (S16 Fig.)

Next, for visualization purposes we selected pairs of active adhesome genes corresponding to scaled Hi-C contacts above 0.5 and regulatory correlation above 0.6 (Supplementary Fig. S6F), resulting in a network of highly spatially clustered and co-regulated genes (121 genes, 255 edges) revealing important chromosomal intermingling at active adhesome gene loci. The network is displayed in Supplementary Fig. S16, with genes colored and grouped per chromosome and edge thickness representing the strength of the regulatory correlation.

Regulatory network of adhesome transcription regulators (S18B Fig.)

Next, we built a regulatory network of adhesome TRs using the TR-target link data (Supplementary Fig. S18B). TRs identified as significant in Figure 3B-C are represented as red nodes, while the remaining TRs are represented as blue nodes. The network is densely connected (242 directed edges among the 26 active TRs targeting active adhesome genes) and 12 TRs have annotated self-targeting relationships, including POLR2A, CTCF, MAZ, CEBPB and LMNB1. Notably, the active adhesome TR regulatory network is typically larger than the TR regulatory networks of randomly selected gene sets and contains significantly more edges (p-value = 0.028, Supplementary Fig. S17E). These findings describe extensive targeting relationships among adhesome TRs in IMR-90 cells. TRs found to significantly target active adhesome genes are also the most central TRs in this network (XL-minimal hypergeometric test [11] with X=1 and L=20, p-value$\leq 6 \cdot 10^{-4}$). Centrality of a TR was measured via its Katz centrality score, which computes the relative influence of a TR through its targets in the directed network (Supplementary Fig. S17F).

Functional enrichment of interacting non-adhesome genes (S24 Fig.)

The LAS analysis also allows us to identify non-adhesome genes that are co-clustered and co-regulated with adhesome genes. Towards this, we considered the genes in non-adhesome interacting regions that interact with adhesome interacting regions. This gene list was further refined by only keeping active genes that showed a strong positive regulatory correlation (upper decile) with the adhesome genes in the LAS submatrices in which they were found (see Supplementary Fig. S25A). This yielded a list of 2,826 active non-adhesome genes that were spatially co-clustered and co-regulated with active adhesome genes. We performed a gene set enrichment analysis on these genes. The significant Gene Ontology (GO) terms (at the 10^{-2} level after adjustment for False Discovery Rate) are reported in the bar chart of Supplementary Fig. S24. Interestingly, many of these GO terms are related to the ubiquitin-proteasome system, which has previously been shown to regulate focal adhesions [12], as well as terms related to vesicle transport, which plays a role in the regulation of adhesome receptors in the context of 3D cell migration [13]. Repeating this analysis using a more stringent regulatory correlation threshold (top percentile) selected 755 active non-adhesome genes and resulted in enrichment for the same GO terms (see Supplementary Fig. S25B-C).

Adhesion-related genes in the supplementary MERFISH network (S28 Fig.)

Interestingly, various of the identified non-adhesome genes are known to be related to adhesion, including PEPD (involved in collagen metabolism), FXYD5 (involved in down-regulation of E-cadherin) and ACTN4 (actin anchor chain). In addition, COLGALT1 is an enzyme that post-translationally modifies collagen proteins. Of interest are also the interactions between ACTN4 and the adhesome genes ITGA3 (integrin alpha 3) and PFN1 (small actin binding protein). We also highlight that many non-adhesome genes in the graph interact with the adhesome gene CRKL (degree = 22), a gene that plays an important role as a kinase to modulate the focal adhesions [14].

References

[1] Hotelling H. New Light on the Correlation Coefficient and its Transforms. Journal of the Royal Statistical Society Series B (Methodological). 1953;15(2):193-232. Publisher: [Royal Statistical Society, Wiley]. Available from: `https://www.jstor.org/stable/2983768`.

[2] Belyaeva A, Venkatachalapathy S, Nagarajan M, Shivashankar GV, Uhler C. Network analysis identifies chromosome intermingling regions as regulatory hotspots for transcription. Proceedings of the National

Academy of Sciences. 2017 Dec;114(52):13714-9. Publisher: Proceedings of the National Academy of Sciences. Available from: https://www.pnas.org/doi/10.1073/pnas.1708028115. doi:10.1073/pnas.1708028115.

[3] Winograd-Katz SE, Fässler R, Geiger B, Legate KR. The integrin adhesome: from genes and proteins to human disease. Nature Reviews Molecular Cell Biology. 2014 Apr;15(4):273-88. Number: 4 Publisher: Nature Publishing Group. Available from: https://www.nature.com/articles/nrm3769. doi:10.1038/nrm3769.

[4] Zhao Y, Garcia BA. Comprehensive Catalog of Currently Documented Histone Modifications. Cold Spring Harbor Perspectives in Biology. 2015 Sep;7(9):a025064. Available from: https://www.ncbi.nlm.nih.gov/pmc/articles/PMC4563710/. doi:10.1101/cshperspect.a025064.

[5] Kanehisa M, Goto S. KEGG: Kyoto encyclopedia of genes and genomes. Nucleic Acids Research. 2000;28(1):27-30.

[6] Kanehisa M. Toward understanding the origin and evolution of cellular organisms. Protein Science. 2019;28(11):1947-51.

[7] Kanehisa M, Furumichi M, Sato Y, Kawashima M, Ishiguro-Watanabe M. KEGG for taxonomy-based analysis of pathways and genomes. Nucleic Acids Research. 2023;51(D1):D587-92.

[8] Oliveros JC. VENNY. An interactive tool for comparing lists with Venn diagrams. http://bioinfogpcnbcsices/tools/venny/indexhtml. 2007. Available from: https://ci.nii.ac.jp/naid/20001505977/.

[9] Zhang Q, Liu W, Zhang HM, Xie GY, Miao YR, Xia M, et al. hTFtarget: A Comprehensive Database for Regulations of Human Transcription Factors and Their Targets. Genomics, Proteomics & Bioinformatics. 2020 Apr;18(2):120-8. Available from: https://www.sciencedirect.com/science/article/pii/S1672022920300954. doi:10.1016/j.gpb.2019.09.006.

[10] Wagner F. The XL-mHG Test For Enrichment: A Technical Report. arXiv:150707905 [stat]. 2015 Sep. ArXiv: 1507.07905. Available from: http://arxiv.org/abs/1507.07905.

[11] Wagner F. The XL-mHG Test For Enrichment: A Technical Report. arXiv:150707905 [stat]. 2015 Sep. ArXiv: 1507.07905. Available from: http://arxiv.org/abs/1507.07905.

[12] Teckchandani A, Cooper JA. The ubiquitin-proteasome system regulates focal adhesions at the leading edge of migrating cells. elife. 2016;5:e17440. Available from: https://www.ncbi.nlm.nih.gov/pmc/articles/PMC5092051/. doi:10.7554/eLife.17440.

[13] Wilson BJ, Allen JL, Caswell PT. Vesicle trafficking pathways that direct cell migration in 3D matrices and in vivo. Traffic. 2018;19(12):899-909. Available from: https://onlinelibrary.wiley.com/doi/abs/10.1111/tra.12605. doi:10.1111/tra.12605.

[14] Li L, Guris DL, Okura M, Imamoto A. Translocation of CrkL to Focal Adhesions Mediates Integrin-Induced Migration Downstream of Src Family Kinases. Molecular and Cellular Biology. 2003 Apr;23(8):2883-92. Available from: https://www.ncbi.nlm.nih.gov/pmc/articles/PMC152569/. doi:10.1128/MCB.23.8.2883-2892.2003.
