# Supplementary material for: Coupling between adhesome assembly and 3D chromatin organization
Source: PLoS One. 2026 Oct 29;21(10):e0361202. doi: 10.1371/journal.pone.0361202 (PMC13719029; doi:10.1371/journal.pone.0361202)
Supplement: Supplementary file 33 [file pone.0361202.s037.pdf]

Response to the Academic Editor and Reviewer

PONE-D-26-28406

Coupling between adhesome assembly and 3D chromatin organization

Dear Dr. Lausen,

We thank you and the reviewer for the constructive comments. We have clarified the hypothesis, streamlined the Results and Discussion, reduced the number of panels in the main figures, and removed repeated methodological descriptions. Below, we respond to each point. Our responses are shown in red; the editor's and reviewer's comments are shown in black. Clean and marked versions of the revised manuscript and Supporting Information have been prepared.

Academic editor

However, the manuscript needs substantial rewriting. Please clearly state the hypothesize and show the data in a concise and efficient way. Supporting material can be added in supplementary data. Please streamline the draft and follow the guidance of the reviewer carefully.

Response. We have revised the Introduction to state the hypothesis explicitly: genes encoding adhesome components are spatially clustered and have similar regulatory profiles, and loci encoding their transcriptional regulators are spatially proximal to adhesome target genes. The revised Introduction also specifies the comparison between active adhesome genes and active non-adhesome genes in fibroblasts.

To focus the presentation, we moved six secondary or overlapping panels from the main figures to the Supporting Information, reducing the main display from 29 to 23 panels across six figures. We shortened the Results and Discussion and moved supporting network and functional-enrichment interpretations to Supplementary Results. The main text retains the principal Hi-C, regulatory, MERFISH and DNA-FISH evidence. We also revised the Abstract and Discussion to distinguish the observed spatial and regulatory associations from a possible role in cellular signal sensing, which remains to be tested. The specific changes are detailed below.

Reviewer 1

The topic of the manuscript is interesting and a tremendous amount of data is presented. Nevertheless, in its current form the manuscript is overloaded with figures and interpretations that are not important for the main story. The authors need to reduce the amount of figures and data presented so that a clear and concise story emerges.

Response. We thank the reviewer for this suggestion. We have reduced the amount of supporting material in the main figures and shortened the accompanying interpretations. The following panels have been moved to the Supporting Information (main-figure numbers below refer to the submitted version; supplementary numbers refer to the revised version):

- Fig. 2D → S16 Fig.: the thresholded network of contacting and co-regulated adhesome genes.

- Fig. 3B and 3E → S18A–B Fig.: the regulatory embedding and interaction network of adhesome transcription regulators.
- Fig. 4C → S24 Fig.: functional enrichment of non-adhesome genes interacting with adhesome regions.
- Fig. 5A → S26 Fig.: MERFISH distance heatmaps using the 500-kb mapping window.
- Fig. 6B → S31 Fig.: the DNA-FISH segmentation and quantification workflow.

The associated supporting network and enrichment descriptions, including the gene-by-gene interpretation of the supplementary MERFISH network, now appear in Supplementary Results. The main Results retains the key comparisons and controls, the LAS network, the shared Hi-C/MERFISH core, and the DNA-FISH images and quantifications. We also removed repeated interpretations concerning signal sensing from the main text. All 31 supplementary figures are now cited in the main text and numbered in order of first appearance, and their captions and cross-references have been updated.

Furthermore, the description of Materials and Methods could be more concise. There is some redundancy between the methods and the results sections, e.g. on the MERFISH analysis.

Response. We have addressed the redundancy by consolidating methodological descriptions. MERFISH acquisition, filtering, probe mapping, distance normalization, averaging over observed probe pairs and control sampling are described together in Methods, Section 2.5. Results focuses on probe coverage, spatial comparisons and the sensitivity analysis using probes that overlap adhesome genes, without repeating the general acquisition and processing description.

We also removed repeated regulatory-feature construction and Hi-C preprocessing descriptions from Results. Conversely, the gene-enrichment findings have been removed from Methods because they are already presented in Results. This separates the procedures from their findings while retaining the experimental protocols and information needed to reproduce the analyses.

Journal requirements

1–2. PLOS style requirements and LaTeX template.

Please ensure that the manuscript meets PLOS ONE's style requirements, including file naming, and update the submission to use the PLOS LaTeX template.

Response. The revised manuscript uses the PLOS-based LaTeX template and the supplied `plos2025` bibliography style. We have updated the author-name presentation and supporting-figure references. The clean manuscript, marked manuscript and response letter have been prepared as separate files for upload under the requested submission categories.

3. Grant information.

The grant information in the Funding Information and Financial Disclosure sections does not match. Please provide the correct grant numbers when resubmitting.

Response. We have checked the grant identifiers and reconciled the Funding Information and Financial Disclosure sections so that they report consistent grant information.

4. Role of funders.

Please state what role the funders took in the study and include the amended Role of Funder statement in the cover letter.

Response. We have included the following statement in the cover letter: The funders had no role in study design, data collection and analysis, decision to publish, or preparation of the manuscript.

5. Funding text in the manuscript.

Please remove any funding-related text from the manuscript. Funding information will be published from the Financial Disclosure section of the online submission form.

Response. We have removed the funding-related text from the manuscript's Acknowledgements and retained only the non-funding acknowledgements.
