# Supplementary material for: Coupling between adhesome assembly and 3D chromatin organization
Source: PLoS One. 2026 Oct 29;21(10):e0361202. doi: 10.1371/journal.pone.0361202 (PMC13719029; doi:10.1371/journal.pone.0361202)
Supplement: Supplementary file 34 [file pone.0361202.s034.html]

OmicsIntegrator2 Results


# OmicsIntegrator2 Results

Download SVG

LPXNGIT1ARF1ARPC2CTTNINSRPTPN6ACTBTLN1SORBS1CBLINPPL1SRCPTPRHBCAR1ABL1LASP1PALLDPAK1PLCG1GRB2STAT3PTPN2PRKCACAPN1PTK2NUDT16L1TGFB1I1FLNATRIOVAV1TRIP6NF2PXNPIP2ADAM12SH3KBP1PPM1MVASPPRKACAJUBPLD1SHC1PTK2BITGB1GAB1CSKMAPK1PIP5K1CDNM2PTPN1ACTN1CSRP1LIMK1CFL1BCAR3PIK3CARICSFYNKCNH2CEACAM1RHOAROCK1CRKSYKSDC4PTPN11PTPN12p190GAPMAPK8CAV1DDEF1PARVARAPGEF1ITGA5MAPK8IP3ARHGEF6HSPB1ARHGAP26SDCBPPTENNCK2TESITGB3BPLPPSORBS2PTPRFABI1THY1SORBS3RAC1CRKLPIP3TESK1VIL2LYNDDEF2VCLSOS1CD47OSTF1AKT1ILKHRASTIAM1GRB7PDPK1PPP2CAARHGAP24INPP5DSIRPAPARVBLAYNKALPHA1PLAURRASA1GNB2L1PLEC1DAGFBLIM1PTPRAPKD1DMNLIMS1IRS1TSPAN1PPFIA1TRPM7CalciumARHGAP5FHL2KTN1DOCK1CALRIP3HSPA2VIMPFN1KEAP1ZYXNEDD9PTPROILKAPSLC9A1ENGMARCKSP190GAPLRP1ENAHELMO1TNS1RAVER1PABPC1PLEKHC1C20orf42NEXNRHOUITGA1SHARPINITGB6CAPN2ITGB3SYNMGIT2ITGA6CASP8ITGAMCD151EZRARHGEF7ITGB5TUBA1BITGA2CIB1ITGB7VAV3VAV2MSNITGAEITGB8ARHGEF2ITGA7ITGB2RDXHAX1SLC3A2AGAP2ITGA8MYH9GRLF1ARHGEF12NRP1SSH1MMP14ITGB4FERMT2NISCHARHGAP32STARD13ITGALITGA3ITGAXASAP2ITGB1BP1ITGA4SRCIN1PPM1FCORO1BABI2SH2B1ITGADITGAVITGA9ASAP3PDE4DPLECITGA10ANKRD28CYTH2DEF6NDEL1PEAK1LDB3ITGA11ABI3DLC1CORO2ALIMS2PRNPCASS4NRP2FABP3SVILMYOM1PVRRNF5

Fix Nodes

Force

| Nodes |  |
| --- | --- |
| Label nodes by | ID |
| Group nodes by | None |
| Shape nodes by | None |
| Size nodes by | None |
| Color nodes by | None |
| Edges |  |
| Edge opacity |  |
| Color edges by | None |
| Size edges by | None |
| Orient edges by | None |
| Only Show Edges | Show All Edges |

⌘+Click a node to open GeneCards

This page generated by Axial
