# Supplementary material for: Coupling between adhesome assembly and 3D chromatin organization
Source: PLoS One. 2026 Oct 29;21(10):e0361202. doi: 10.1371/journal.pone.0361202 (PMC13719029; doi:10.1371/journal.pone.0361202)
Supplement: Peer review history

# Peer review: Decision Letter 0

**DOI:** 10.1371/journal.pone.0361202.r001

**Authors:** Jörn Lausen

24 Aug 2026

Dear Dr. Shivashankar,

Thank you for submitting your manuscript to PLOS One. After careful consideration, we feel that it has merit but does not fully meet PLOS One’s publication criteria as it currently stands. Therefore, we invite you to submit a revised version of the manuscript that addresses the points raised during the review process.

Please submit your revised manuscript by Oct 23 2026 11:59PM. If you will need more time than this to complete your revisions, please reply to this message or contact the journal office at plosone@plos.org. When you're ready to submit your revision, log on to https://www.editorialmanager.com/pone/ and select the 'Submissions Needing Revision' folder to locate your manuscript file.

- A letter that responds to each point raised by the academic editor and reviewer(s). You should upload this letter as a separate file labeled 'Response to Reviewers'.
- A marked-up copy of your manuscript that highlights changes made to the original version. You should upload this as a separate file labeled 'Revised Manuscript with Track Changes'.
- An unmarked version of your revised paper without tracked changes. You should upload this as a separate file labeled 'Manuscript'.

If you would like to make changes to your financial disclosure, please include your updated statement in your cover letter. Guidelines for resubmitting your figure files are available below the reviewer comments at the end of this letter.

If applicable, we recommend that you deposit your laboratory protocols in protocols.io to enhance the reproducibility of your results. Protocols.io assigns your protocol its own identifier (DOI) so that it can be cited independently in the future. For instructions see: https://journals.plos.org/plosone/s/submission-guidelines#loc-laboratory-protocols. Additionally, PLOS One offers an option for publishing peer-reviewed Lab Protocol articles, which describe protocols hosted on protocols.io. Read more information on sharing protocols at https://plos.org/protocols?utm_medium=editorial-email&utm_source=authorletters&utm_campaign=protocols.

As the corresponding author, your ORCID iD is verified in the submission system and will appear in the published article. PLOS supports the use of ORCID, and we encourage all coauthors to register for an ORCID iD and use it as well. Please encourage your coauthors to verify their ORCID iD within the submission system before final acceptance, as unverified ORCID iDs will not appear in the published article. *Only*  the individual author can complete the verification step; PLOS staff *cannot*  verify ORCID iDs on behalf of authors.

We look forward to receiving your revised manuscript.

Kind regards,

Jörn Lausen, Ph.D. (Dr. rer nat)

Academic Editor

PLOS One

Journal Requirements:

When submitting your revision, we need you to address these additional requirements.

1. Please ensure that your manuscript meets PLOS ONE's style requirements, including those for file naming. The PLOS ONE style templates can be found at https://journals.plos.org/plosone/s/file?id=wjVg/PLOSOne_formatting_sample_main_body.pdf and https://journals.plos.org/plosone/s/file?id=ba62/PLOSOne_formatting_sample_title_authors_affiliations.pdf

2. Please update your submission to use the PLOS LaTeX template. The template and more information on our requirements for LaTeX submissions can be found at http://journals.plos.org/plosone/s/latex.

3. We note that the grant information you provided in the ‘Funding Information’ and ‘Financial Disclosure’ sections do not match.

When you resubmit, please ensure that you provide the correct grant numbers for the awards you received for your study in the ‘Funding Information’ section.

4. Thank you for stating the following financial disclosure:

[This work was supported by the National Center for Complementary and Integrative Health/National 1037

Institutes of Health (1DP2AT012345 to C.U.); the Office of Naval Research (N00014-22-1-2116 to C.U.); the 1038

National Science Foundation (DMS-1651995 to C.U.); the MIT-IBM Watson AI Lab; the MIT J-Clinic for 1039

Machine Learning and Health; the Eric and Wendy Schmidt Center at the Broad Institute; a Simons 1040

Investigator Award (to C.U.); the Swiss National Foundation (310030 208046 to G.V.S.); ETH Z¨rich (to 1041

G.V.S.) and the Paul Scherrer Institut (to G.V.S.).].

Please state what role the funders took in the study.  If the funders had no role, please state: "The funders had no role in study design, data collection and analysis, decision to publish, or preparation of the manuscript."

If this statement is not correct you must amend it as needed.

Please include this amended Role of Funder statement in your cover letter; we will change the online submission form on your behalf.

5. Please remove any funding-related text from the manuscript. Please note that funding information should not appear in any section or other areas of your manuscript. We will publish the funding statement that is present in the Financial Disclosure section of the online submission form.

If the reviewer comments include a recommendation to cite specific previously published works, please review and evaluate these publications to determine whether they are relevant and should be cited. There is no requirement to cite these works unless the editor has indicated otherwise.

Additional Editor Comments:

Dear authors,

your manuscript has been reviewed by an expert in the field and also carefully examined by myself. We see merit in your data. However, the manuscript needs substantial rewriting. Please clearly state the hypothesize and show the data in a concise and efficient way. Supporting material can be added in supplementary data. Please streamline the draft and follow the guidance of the reviewer carefully. Upon resubmission the manuscript will be reevaluated.

[Note: HTML markup is below. Please do not edit.]

Reviewers' comments:

Reviewer's Responses to Questions

**Comments to the Author**

1. Is the manuscript technically sound, and do the data support the conclusions?

Reviewer #1: Yes

**********

2. Has the statistical analysis been performed appropriately and rigorously?

Reviewer #1: Yes

**********

3. Have the authors made all data underlying the findings in their manuscript fully available?

The PLOS Data policy

Reviewer #1: Yes

**********

4. Is the manuscript presented in an intelligible fashion and written in standard English?

Reviewer #1: Yes

**********

Reviewer #1: The topic of the manuscript is interesting and a tremendous amount of data is presented. Nevertheless, in its current form the manuscript is overloaded with figures and interpretations that are not important for the main story. The authors need to reduce the amount of figures and data presented so that a clear and concise story emerges. Furthermore, the description of Materials and Methods could be more concise. There is some redundancy between the methods and the results sections, e.g. on the MERFISH analysis.

**********

what does this mean?). If published, this will include your full peer review and any attached files.

If you choose “no”, your identity will remain anonymous but your review may still be made public.

**Do you want your identity to be public for this peer review?**  For information about this choice, including consent withdrawal, please see our Privacy Policy

Reviewer #1: No

**********

[NOTE: If reviewer comments were submitted as an attachment file, they will be attached to this email and accessible via the submission site. Please log into your account, locate the manuscript record, and check for the action link "View Attachments". If this link does not appear, there are no attachment files.]

To ensure your figures meet our technical requirements, please review our figure guidelines: https://journals.plos.org/plosone/s/figures

You may also use PLOS’s free figure tool, NAAS, to help you prepare publication quality figures: https://journals.plos.org/plosone/s/figures#loc-tools-for-figure-preparation.

NAAS will assess whether your figures meet our technical requirements by comparing each figure against our figure specifications.

# Author comment: Author response to Decision Letter 1

**DOI:** 10.1371/journal.pone.0361202.r002

25 Sep 2026

Response to the Academic Editor and Reviewer

PONE-D-26-28406

Coupling between adhesome assembly and 3D chromatin organization

Dear Dr. Lausen,

We thank you and the reviewer for the constructive comments. We have clarified the hypothesis, streamlined the Results and Discussion, reduced the number of panels in the main figures, and removed repeated methodological descriptions. Below, we respond to each point. Our responses are shown in red; the editor’s and reviewer’s comments are shown in black. Clean and marked versions of the revised manuscript and Supporting Information have been prepared.

Academic editor

- However, the manuscript needs substantial rewriting. Please clearly state the hypothesize and show the data in a concise and efficient way. Supporting material can be added in supplementary data. Please streamline the draft and follow the guidance of the reviewer carefully. Response. We have revised the Introduction to state the hypothesis explicitly: genes encoding adhesome components are spatially clustered and have similar regulatory profiles, and loci encoding their transcriptional regulators are spatially proximal to adhesome target genes. The revised Introduction also specifies the comparison between active adhesome genes and active non-adhesome genes in fibroblasts.

* To focus the presentation, we moved six secondary or overlapping panels from the main figures to the Supporting Information, reducing the main display from 29 to 23 panels across six figures. We shortened the Results and Discussion and moved supporting network and functional-enrichment

interpretations to Supplementary Results. The main text retains the principal Hi-C, regulatory, MERFISH and DNA-FISH evidence. We also revised the Abstract and Discussion to distinguish the observed spatial and regulatory associations from a possible role in cellular signal sensing, which

remains to be tested. The specific changes are detailed below.

Reviewer 1

- The topic of the manuscript is interesting and a tremendous amount of data is presented. Nevertheless, in its current form the manuscript is overloaded with figures and interpretations that are not important for the main story. The authors need to reduce the amount of figures and data presented so that a clear and concise story emerges.

* We thank the reviewer for this suggestion. We have reduced the amount of supporting material in the main figures and shortened the accompanying interpretations. The following panels have been moved to the Supporting Information (main-figure numbers below refer to the submitted version; supplementary numbers refer to the revised version):

• Fig. 2D → S16 Fig.: the thresholded network of contacting and co-regulated adhesome genes.

• Fig. 3B and 3E → S18A–B Fig.: the regulatory embedding and interaction network of adhesome

transcription regulators.

• Fig. 4C → S24 Fig.: functional enrichment of non-adhesome genes interacting with adhesome

regions.

• Fig. 5A → S26 Fig.: MERFISH distance heatmaps using the 500-kb mapping window.

• Fig. 6B → S31 Fig.: the DNA-FISH segmentation and quantification workflow.

The associated supporting network and enrichment descriptions, including the gene-by-gene interpretation of the supplementary MERFISH network, now appear in Supplementary Results. The main Results retains the key comparisons and controls, the LAS network, the shared Hi-C/MERFISH core, and the DNA-FISH images and quantifications. We also removed repeated interpretations concerning signal sensing from the main text. All 31 supplementary figures are now cited in the main text and numbered in order of first appearance, and their captions and

cross-references have been updated.

- Furthermore, the description of Materials and Methods could be more concise. There is some redundancy between the methods and the results sections, e.g. on the MERFISH analysis.

* We have addressed the redundancy by consolidating methodological descriptions. MERFISH acquisition, filtering, probe mapping, distance normalization, averaging over observed probe pairs and control sampling are described together in Methods, Section 2.5. Results focuses on probe coverage, spatial comparisons and the sensitivity analysis using probes that overlap adhesome genes, without repeating the general acquisition and processing description. We also removed repeated regulatory-feature construction and Hi-C preprocessing descriptions from Results. Conversely, the gene-enrichment findings have been removed from Methods because they are already presented in Results. This separates the procedures from their findings while retaining the experimental protocols and information needed to reproduce the analyses.

Journal requirements

- 1–2. PLOS style requirements and LaTeX template. Please ensure that the manuscript meets PLOS ONE’s style requirements, including file naming, and update the submission to use the PLOS LaTeX template.

* The revised manuscript uses the PLOS-based LaTeX template and the supplied plos2025 bibliography style. We have updated the author-name presentation and supporting-figure references. The clean manuscript, marked manuscript and response letter have been prepared as separate files for upload under the requested submission categories.

- 3. Grant information. The grant information in the Funding Information and Financial Disclosure sections does not match. Please provide the correct grant numbers when resubmitting.

* We have checked the grant identifiers and reconciled the Funding Information and Financial Disclosure sections so that they report consistent grant information.

- 4. Role of funders. Please state what role the funders took in the study and include the amended Role of Funder statement in the cover letter.

* We have included the following statement in the cover letter: The funders had no role in study design, data collection and analysis, decision to publish, or preparation of the manuscript.

- 5. Funding text in the manuscript. Please remove any funding-related text from the manuscript. Funding information will be published from the Financial Disclosure section of the online submission form.

* We have removed the funding-related text from the manuscript’s Acknowledgements and retained only the non-funding acknowledgements.

Attachment Submitted filename: Adhesome_PLOSOne_Submission_review_response.pdf

# Editor report: Decision Letter 1

**DOI:** 10.1371/journal.pone.0361202.r003

**Authors:** Jörn Lausen

7 Oct 2026

Coupling between adhesome assembly and 3D chromatin organization

PONE-D-26-28406R1

Dear Dr. Shivashankar,

We’re pleased to inform you that your manuscript has been judged scientifically suitable for publication and will be formally accepted for publication once it meets all outstanding technical requirements.

Within one week, you’ll receive an e-mail detailing the required amendments. When these have been addressed, you’ll receive a formal acceptance letter and your manuscript will be scheduled for publication.

An invoice will be generated when your article is formally accepted. Please note, if your institution has a publishing partnership with PLOS and your article meets the relevant criteria, all or part of your publication costs will be covered. Please make sure your user information is up-to-date by logging into Editorial Manager at Editorial Manager® and clicking the ‘Update My Information' link at the top of the page. For questions related to billing, please contact billing support.

If your institution or institutions have a press office, please notify them about your upcoming paper to help maximize its impact. If they’ll be preparing press materials, please inform our press team as soon as possible -- no later than 48 hours after receiving the formal acceptance. Your manuscript will remain under strict press embargo until 2 pm Eastern Time on the date of publication. For more information, please contact onepress@plos.org.

Kind regards,

Jörn Lausen, Ph.D. (Dr. rer nat)

Academic Editor

PLOS One

Additional Editor Comments (optional):

Reviewers' comments:

# Editor report: Acceptance letter

**DOI:** 10.1371/journal.pone.0361202.r004

**Authors:** Jörn Lausen

PONE-D-26-28406R1

PLOS One

Dear Dr. Shivashankar,

I'm pleased to inform you that your manuscript has been deemed suitable for publication in PLOS One. Congratulations! Your manuscript is now being handed over to our production team.

At this stage, our production department will prepare your paper for publication. This includes ensuring the following:

* All references, tables, and figures are properly cited

* All relevant supporting information is included in the manuscript submission,

* There are no issues that prevent the paper from being properly typeset

You will receive further instructions from the production team, including instructions on how to review your proof when it is ready. Please keep in mind that we are working through a large volume of accepted articles, so please give us a few days to review your paper and let you know the next and final steps.

Lastly, if your institution or institutions have a press office, please let them know about your upcoming paper now to help maximize its impact. If they'll be preparing press materials, please inform our press team within the next 48 hours. Your manuscript will remain under strict press embargo until 2 pm Eastern Time on the date of publication. For more information, please contact onepress@plos.org.

You will receive an invoice from PLOS for your publication fee after your manuscript has reached the completed accept phase. If you receive an email requesting payment before acceptance or for any other service, this may be a phishing scheme. Learn how to identify phishing emails and protect your accounts at https://explore.plos.org/phishing.

If we can help with anything else, please email us at customercare@plos.org.

Thank you for submitting your work to PLOS One and supporting open access.

Kind regards,

PLOS One Editorial Office Staff

on behalf of

Dr. Jörn Lausen

Academic Editor

PLOS One
